# A scoping review of music-based pain treatment mechanism research

**DOI:** 10.1097/PR9.0000000000001467

**Published:** 2026-07-22

**Authors:** Joke Bradt, Adam Hirsh, Mark P. Jensen, Elise Desbarats, Debra S. Burns, Jeffery A. Dusek, Eric L. Garland, Raluca D. Georgescu, Lynn Gumert, Kobe Hanson, Jannatul Khawsar, Tanya Smit, Mathieu Roy

**Affiliations:** a Department of Creative Arts Therapies, Drexel University, Philadelphia, PA, USA; b Department of Psychology, Indiana University Indianapolis, Indianapolis, IN, USA; c Department of Rehabilitation Medicine, University of Washington, Seattle, WA, USA; d Department of Psychology, McGill University, Montréal, Canada; e Alan Edwards Centre for Research on Pain, McGill University, Montréal, Canada; f College of Communication and Fine Arts, The University of Memphis, Memphis, TN, USA; g Susan Samueli Integrative Health Institute and Department of Medicine, University of California- Irvine, Irvine, CA, USA; h Department of Psychiatry, University of California San Diego, La Jolla, CA, USA; i Sanford Institute for Empathy and Compassion, University of California San Diego, La Jolla, CA, USA; j Department of General Psychology, University of Padova, Padova, Italy; k Center for Research Excellence in Supportive Care (CREST), Division of Palliative Medicine, Department of Internal Medicine, The Ohio State University Wexner Medical Center, Columbus, OH, USA

**Keywords:** Music-based interventions, Music-induced hypoalgesia, Mechanisms, Pain

## Abstract

Research supports the efficacy of music-based interventions (MBIs) for pain management, but understanding of their underlying mechanisms is lagging. This scoping review aims to map existing mechanism-focused MBI research, identify knowledge gaps, and highlight the strengths and weaknesses of this body of research. Studies were eligible if they tested a mechanism, moderator, or predictor of the effects of MBIs on clinical or experimental pain and were published in English in a peer-reviewed journal. Following the Joanna Briggs Institute scoping review methodology, we searched MEDLINE, Embase, PsycINFO, PubMed, and Scopus from inception through February 2026. Two reviewers completed screening and data extraction, with discrepancies resolved by a third reviewer. From 663 records screened, 57 studies were included. Most studies employed experimental pain models with healthy volunteers. All but 4 studies examined the mechanisms of music audio recordings; the remaining studies examined active music-making. We identified convergent evidence for several candidate mechanisms, including positive emotional valence, cognitive agency, and sensorimotor synchronization. Evidence from neuroimaging studies point to the impact of music on early stages of pain processing as well as on higher-order cognitive and affective interpretation of pain. Finally, preliminary evidence suggests that music may restore default mode network connectivity involved in self-referential thoughts. Across studies, several methodological limitations constrained inferences. Future research should (1) employ rigorous designs that distinguish between competing mechanistic models and, when feasible, incorporate causal manipulations to test hypothesized mechanisms and (2) examine whether music influences pain processing differently in populations with vs without clinical pain.

## Abstract 2

Supplemental Digital Content is Available in the Text.

This scoping review summarizes the evidence of mechanisms underlying the hypoalgesic effects of music and highlights the strengths and weaknesses of this body of research.

## 1. Introduction

Acute and chronic pain are among the most common and costly health problems facing society.^13,32^ Commonly used treatments such as opioids, nonsteroidal anti-inflammatory agents, and epidural injections bring limited relief to a small percentage of responders, yet prevalent side effects and addictive potential pose a substantial risk of harm.^19,77^ As a result, people are increasingly seeking complementary approaches to pain management.^9^

Research supports music-based interventions (MBIs) as promising approaches for acute and chronic pain management.^11,43,54,83,87^ In clinical care, MBIs range from patient- or provider-initiated music listening (*music medicine*) to music experiences facilitated by a trained music therapist (*music therapy*).^2^ Systematic reviews indicate medium to large effects of MBIs on acute pain (standardized mean differences [SMDs]: 0.65–0.91)^11,43,54,87^ and medium effects on chronic pain (SMD = 0.60).^38^

However, like other mind-body interventions, MBIs are often dismissed as “adjunctive.” As a result, they are rarely recommended despite effect sizes that are comparable to those of more conventional pharmacotherapies such as opioids (SMD = 0.60)^34^ and minimal negative side effects. Insights into their mechanisms of action could help increase clinical credibility and uptake and guide more precise application of MBIs into patient care.

Mechanistic clinical trials are designed to “understand a biological or behavioral process, the pathophysiology of a disease, or the mechanism of action of an intervention.”^66^ Different research designs provide varying levels of mechanistic evidence. The strongest evidence arises from experimental manipulation of a candidate mechanism variable while controlling for confounds (eg, comparing pleasant vs unpleasant music while holding arousal and familiarity constant). However, in practice, full experimental control is rarely possible. In such cases, studies that combine experimental manipulation with measures of the candidate mediator (eg, perceived pleasure) and formal testing of mediation provide stronger evidence than either approach alone. Follow-up work using pharmacological probes (eg, agonists/antagonists) or neuromodulation (eg, temporarily stimulate or inhibit brain activity) can then test causality more definitively.

**Figure 1.**
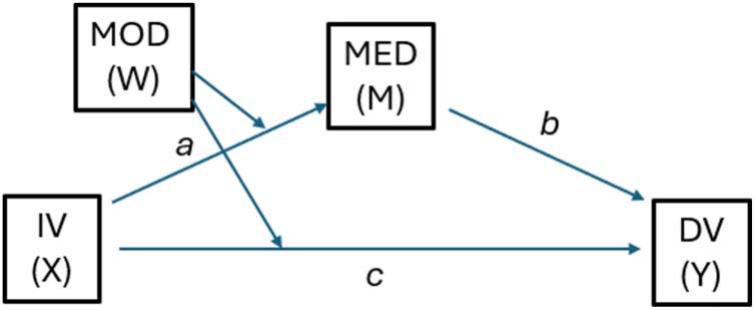
A path diagram for a single mediator model, including a potential moderator. IV = independent variable; DV = dependent variable; MED = mediator; MOD = moderator.

Mediation analyses are commonly used in mechanism research. A typical mediation model (Fig. 1) includes a treatment (X, eg, MBI vs control), a mediator (M, eg, mood), and an outcome variable (Y, eg, pain intensity). Mediation analysis estimates whether the effect of treatment on outcome (*c* path) operates partly through the mediator (*a* and *b* paths).^60,61^ The treatment-to-mediator (*a* path: music→mood) and treatment-to-outcome (*c* path: music→pain) paths are considered causal in randomized designs. However, the mediator-to-outcome (*b* path: mood→pain) path is correlational when the M and Y variables are measured concurrently. Testing for temporal precedence (ie, whether changes in M precede changes in Y) can strengthen mechanistic inference. In short, mediation studies can identify *potential* mechanisms, but additional experimental work is needed to establish causality.

Moderator and prediction studies also contribute to the evidence landscape. Moderators (W) do not provide mechanistic evidence directly. However, they can clarify boundary conditions and suggest mechanistic pathways for further testing. For example, if biological sex is a significant moderator of the effects of MBI vs other pain treatments, sex hormones might be a mechanism variable for MBIs but not the other treatments. Prediction studies examine direct (zero-order) associations, such as whether higher baseline pain sensitivity predicts response across all treatment conditions (ie, music vs other pain treatments). In this case, pain sensitivity would have the potential to be a shared mechanism variable and could then be tested as such in a mechanism trial using mediation or experimental designs. In this way, moderator and predictor studies do not directly test mechanism variables but can generate hypotheses about factors that could be tested in mechanism studies.

Although the literature contains a high-level summary of factors commonly proposed to contribute to music-induced hypoalgesia,^59^ a scoping review of mechanistic research is this area has not yet been conducted. This scoping review therefore seeks to map the existing evidence concerning the mechanisms underlying music-induced hypoalgesia, identify mechanism variables that warrant closer examination, highlight knowledge gaps, and provide a conceptual framework for interpreting mechanistic, moderation, and prediction research.

## 2. Methods

### 2.1. Design

We conducted a scoping review, an approach well-suited for summarizing current evidence and key concepts, identifying knowledge gaps and highlighting the strengths and weaknesses of the existing literature.^71^ We followed the Joanna Briggs Institute updated guidelines for scoping reviews.^71^ A review protocol was developed a priori and approved by the study team, but it was not preregistered.

### 2.2. Study eligibility criteria

Because the primary purpose of the review was to examine mechanistic evidence, rather than conduct a broad survey of research related to MBIs for pain (eg, Refs. 22, 54), we included only studies with designs that would allow for testing of mechanisms, moderators, or predictors. Efficacy and process studies were excluded unless they reported analyses examining mechanisms, moderators, or predictors. The eligibility criteria, organized by the JBI population, concept, context (PCC) elements, are presented in Table 1.

**Table 1:** Inclusion criteria based on the JBI population, concept, and context (PCC) elements.

PCC element	Studies were eligible, if they
Population	(1) Examined either (a) a clinical pain population (eg, naturally occurring pain, medical procedures), or (b) healthy volunteers exposed to experimentally induced pain
Concept	(2) Tested a mechanism or mediator, moderator, or predictor of the effects of music or an MBI on clinical or experimental pain. Studies without explicit mechanistic aims were included if secondary outcomes (ie, additional outcomes that provide supportive information about MBIs' effect on the primary outcome^63^) were described in mechanistic terms (3) Included at least 1 music-only or MBI condition (4) Included at least 1 nonmusic or non-MBI condition or another music or MBI comparator (5) Included at least 1 pain outcome (eg, pain intensity, pain unpleasantness, pain interference, pain threshold, pain tolerance)
Context	(6) Took place in a laboratory setting, clinical setting, community setting, or home environment. There were no restrictions with respect to environment or geographical location
Type of evidence sources	(7) Employed either a parallel-arm or cross-over/repeated measures design. Random allocation was noted when reported, but was not an inclusion criterion. We also included observational studies with repeated measures (eg, ecological momentary assessment of the impact of daily music listening). We excluded efficacy-focused trials, review articles, case reports, and animal studies. We did not include gray literature (eg, conference abstract) (8) Were published in English in a peer-reviewed journal

### 2.3. Search strategy

We searched 5 electronic databases (Medline, Embase, PsycInfo, PubMed, and Scopus) from inception through February 20, 2026. We reviewed the reference lists of included studies to identify additional studies, but we did not review gray literature. The full search strategy is detailed in the supplemental digital content (see materials, http://links.lww.com/PR9/A422).

### 2.4. Screening and data extraction

We used Covidence^86^ for study screening and data extraction. After removing duplicate records, titles and abstracts were independently screened by 2 team members (J.B., M.R.). Two team members independently extracted the data from studies that passed the initial screen using a customized form (see supplemental digital content, materials, http://links.lww.com/PR9/A422), with consensus decisions made by J.B. Disagreements were resolved through team discussion.

We did not conduct a formal quality appraisal. Scoping reviews typically do not include a risk of bias assessment of the included studies, as their focus is on mapping the breadth of evidence rather than evaluating the methodological rigor or risk of bias.^82^ However, we extracted and summarized key methodological features of the included studies (Table 2).

**Table 2:** Characteristics of included studies.

Author (y)	Pain model/noxious stimulus type	Study popula-tion	Design/allocation	N	Music condition(s)	Control(s)	Mechanism variables	Moderator variables	Predictor variables	Pain outcomes	Type of analyses
Abrahan 2026^1^	Exp/Heat	HV	Cross-over/R	28	Music listening: 1. Pleasant-activating (n = 28) 2. Unpleasant-activating (n = 28) 3. Neutral-nonactivating (n = 28)	Silence with pain (n = 28)	Valence (SAM, facial EMG), arousal (SAM, SCR)			PI (VAS)	Experimental manipulation, prediction
Anglin 2021^3^	Clin	Urological procedures	Parallel/R	91	Music listening (n = 53)	Silence (n = 38)		“Gender”*		PI (NRS)	Moderation
Arican 2025^5^	Exp/Cold P	HV	Parallel/R	123	Music listening: 1. Passive music listening (n = 31) 2. Music-and-attention-to- music (MAM) (n = 30) 3. Music-and-attention-to-pain (MAP) (n = 31)	Silence (n = 31)	Distraction			PT	Experimental manipulation
Basiński 2021^10^	Exp/cold P	HV	Cross-over/R	76	Music listening: 1. Arousal music (n = 76) 2. Valence music (n = 76) 3. Depth music (n = 76)	White noise (n = 76)			Music preference (Likert)	PI (NRS), PTh, PT, pain controllability (NRS)	Prediction
Becker 2025^12^	Exp/Electr S	HV	Cross-over/R	84	Music listening: 1. Self-chosen music (n = 84) 2. Researcher-chosen music (n = 84)	Podcast (n = 84)	Valence (SAM), anxiety (STAI-6), HRV	Socio-cultural background (Personal cultural capital; parental cultural capital; education level)		PT, PTh, PI (NRS), PU (NRS)	*a* path, moderation
Bradt 2024^15^	Clin	Advanced cancer	Parallel/R	92	Group music therapy (n = 45)	Social attention control group (n = 47)	Anxiety (PROMIS), emotional support (PROMIS), pain-related self-efficacy (PROMIS), mood (PANAS)	Outcome expectancy (CEQ), music reward (BMRQ), adult playfulness (SMAP), baseline pain interference (PROMIS)		PI (PROMIS), pain interference (PROMIS), perception of change (PGIC)	True mediation, moderated mediation
Chai 2020^16^	Exp/cold P	HV	Cross-over/R	60	Music listening (n = 60)	Silence (n = 60)			Pain catastrophizing (PCS), anxiety (PROMIS), depression (PROMIS)	PI (NRS), PTh, PT, temporal summation of pain, conditioned pain modulation, painful after sensation	Prediction
Choi 2018^17^	Exp/Cold P	HV	Cross-over/R	50	Music listening (n = 50)	1. News listening (n = 50) 2. Silence (n = 50)		Anxiety sensitivity (ASI-16), pain anxiety (PASS-20)		PI (NRS), PTh, PT, PU (NRS)	Moderation
Choi 2022^18^	Exp/Cold P	HV	Cross-over/R	49	Music listening (n = 49)	1. News listening (n = 49) 2. Silence (n = 49)		Anxiety sensitivity (ASI-16, ASI-R), state anxiety (STAI-S), trait anxiety (STAI-T), pain anxiety (PASS-20)		PI (VAS), PT, PU (VAS)	Moderation
Çift 2020^20^	Clin	Extracorporeal shock wave lithotripsy	Parallel/R	150	Music listening: 1. Turkish art music (n = 30) 2. Western art music (n = 30) 3. Patient-preferred music (n = 30)	1. Silence with headphones (n = 30) 2. Silence without headphones (n = 30)	State anxiety (STAI-T)			PI (VAS), willingness to repeat the procedure (Likert), satisfaction (Likert)	*a* path
Colebaugh 2023^21^	Exp/Cold P	HV	Cross-over/R	70	Music listening: 1. Relaxing music (n = 70) 2. Participants' favorite music (n = 70)	White noise (n = 70)	Situational pain catastrophizing (SPCS)			PTh, PT, temporal summation of pain, conditioned pain modulation	*a* and *b* path
Deng 2022^24^	Clin	Breast cancer	Parallel/R	160	1. Music listening (n = 40) 2. Music listening + aromatherapy (n = 40)	1. TAU (n = 40) 2. Aromatherapy (n = 40)	IL-6, HMGB-1 (ELISA) and anxiety (VAS)			PI (VAS)	*a* and *b* path
Dobek 2014^25^	Exp/Heat	HV	Cross-over/R	12	Music listening (n = 12)	Silence (n = 12)	Neural activity of brain, brain stem, and spinal cord (fMRI)			PI (NRS)	*a* path
Du 2022^27^	Clin	Chronic pain	Parallel/R	33	Music listening (n = 17)	TAU (n = 16)	Oxyhemoglobin, deoxyhemoglobin concentration (fNIRS)			PI (VAS and HRV measured by ECG)	*a* path
Dunbar 2021^28^	Exp/Roman chair	HV	Cross-over/unclear	Exp 1: N = 33 Exp 2: N = 109	Exp 1: 1. Music listening without movement (n = 19) 2. Music listening with head nods (n = 14) Exp 2: 1. Music listening with head nods (n = 35) 2. Music listening with foot taps (n = 39)	Exp 1: None Exp 2: Nature sounds with head nods (n = 35)			Exp 1: Music liking, perceived musicality, music familiarity, music enjoyment (NRS for all), music listening frequency	PTh	Experimental manipulation, prediction
Ernberg 2020^29^	Exp/Chem S	HV	Cross-over/R	20	Music listening: 1. Black metal music (n = 20) 2. Classical music (n = 20)	Silence (n = 20)	Anxiety (VAS)			PI (VAS), pain quality (MPQ), pain area/location(pain drawing), pain duration (VAS)	*a* and *b* path
Evers 2024^30^	Exp/Mech P, Heat, Cold S	HV	Cross-over/NR	20	Music listening: 1. Pleasant music (n = 20) 2. Unpleasant music (n = 20)	1. Baseline (n = 20) 2. Silence (n = 20)	Valence (Eindrucks differential)			PTh, cold detection threshold, warm detection threshold, heat PTh, mechanical detection threshold, mechanical PTh, windup ratio, vibration detection threshold, pain pressure threshold (QST measures)	Experimental manipulation
Finlay 2016^33^	Exp/Cold P	HV	Cross-over/R	41	Music listening: 1. Happy (n = 41) 2. Sad (n = 41) 3. Relaxing (n = 41)	Silence (n = 41)	Happy-sad, arousal, distraction, anxiety (NRS for all)			PI, PT, perceived control over pain (NRS for all)	Experimental manipulation
Garcia 2016^35^	Exp/Cold P	HV	Cross-over/NR	30	Music listening: 1. Motivating music (n = 30) 2. Relaxing music (n = 30)	Silence (n = 30)	Mood (POMS), HR, BP		Arousal (HR, BP)	PI, PU (VAS for all)	*a* path
Garza- Villarreal 2012^36^	Exp/Heat	HV	Cross-over/NR	48	Music listening: 1. Mozart (n = 48) 2. Mozart (n = 48)	1. Rain (n = 48) 2. Water (n = 48) 3. PASAT (n = 48) 4. Pink Noise (n = 48)	Distraction, valence, arousal (Likert scale for all)			PI, PU (VAS for all)	*a* and *b* path
Garza-Villarreal 2015^37^	Clin	Fibromyalgia	Cross-over/R	20	Music listening (n = 20)	Pink noise (n = 20)	Neural correlates (fMRI)			PI, PU (VRS for both)	*a* and *b* path
Goldfine 2025^39^	Clin	Acute musculo-skeletal back pain	Parallel/R	40	Self-selected music listening (n = 21)	Noise cancelling headphones (n = 19)	Postintervention anxiety (PROMIS)			PI (NRS)	Mediation
Hekmat 1993^42^	Exp/Cold P	HV	Parallel/R	80	Music listening: 1. Preferred music (n = 20) 2. Nonpreferred music (n = 20)	1. Silence, experimenter present (n = 20) 2. Silence, experimenter absent (n = 20)	Music preference (NRS)			PI (NRS), PT	Experimental manipulation
Howlin 2021^45^	Exp/Cold P	HV	Cross-over/R	49	6 conditions combining 2 types of music (relaxing music vs motivating music) with 3 levels of choice (no choice vs perceived choice from 2 songs vs perceived choice from 4 songs) (n = 49)		Cognitive agency†		Intramusical features†, age, musicality (GMSI), music enjoyment (NRS)	PI (NRS) PT, PU (NRS)	Experimental manipulation, prediction
Howlin 2022^46^	Clin	Acute pain	Parallel/R	286	Music listening: 1. No choice/low complexity (n = 73) 2. No choice/high complexity (n = 70) 3. Perceived choice/low complexity (n = 67) 4. Perceived choice/high complexity (n = 76)		Cognitive agency†			PI (NRS), PU (GEMS)	Experimental manipulation
Hsieh 2014^47^	Exp/Heat	HV	Parallel/R	36	Music listening (n = 12)	1. Frequency-filtered noise (n = 12) 2. Silence (n = 12)		Expectations for pain relief (NRS)		PI, PU (VAS for all)	Moderation
Johnson 2020^48^	Exp/Cold P	HV	Parallel/R	63	Music listening: 1. Music with relaxation suggestions (n = 21) 2. Music alone (n = 21)	Silence (n = 21)	Anxiety (state and trait) (STICSA), mood (PANAS), relaxation (VAS), perceived pain control (VAS), distraction, response expectancy (VAS), absorption (VAS)	Hypnotizability (EHS)		PI (VAS), PTh, PT, PU (VAS)	*b* path, moderation
Kavak Akelma 2020^50^	Clin	Hernia surgery	Parallel/R	117	Music listening (n = 58)	TAU (n = 59)	HR, SBP, DBP, MAP, state anxiety (STAI-1)			PI (NRS)	*a* path
Kenntner-Mabiala 2007^51^	Exp/Heat	HV	Cross-over/R	38	6 music listening conditions combining 3 music tempi [slow (n = 38) vs medium (n = 38) vs fast (n = 38)] with 2 modes (major [n = 38] vs minor [n = 38])		Valence, arousal, happiness, sadness (NRS for all) HR (EKG), RR (Capnometer), end-tidal PC02 (PetCO2)	Sex assigned at birth		PI, PU (NRS)	*a* path, moderation
Knox 2011^52^	Exp/Cold P	HV	Secondary analysis	72	N/A	N/A			Musical parameters (tempo, rhythm, timbre, dynamics)	PI (VAS), PT	N/A
Lad 2022^53^	Exp/Mech P	HV	Cross-over/R	34	Music listening: 1. Music alone (n = 34) 2. Music with tactile vibrations (n = 34)	Tactile vibrations only (n = 34)		Music preference		PI, PTh	Moderation
LiKamWa 2022^55^	Exp/Cold S	HV	Cross-over/R	40	1. Singing (n = 40) 2. Music listening (n = 40)	Silence (n = 40)	Distraction, positive/negative affect (PANAS)		Music training, frequency, singing proficiency (NRS)	PI (NRS), PTh, PT, pain modulation	*a* path, prediction
Linnemann 2015^56^	Clin	Adults, fibromyalgia	Observational	30	Music listening (n = 30)		Salivary cortisol, salivary alpha-amylase		Happy-sad (VAS), arousal (VAS), frequency of music listening (VAS)	PI (VAS), perceived control over pain (Likert scale)	*a* path, prediction
Lu 2019^58^	Exp/Heat	HV	Cross-over/NR	30	Music listening (n = 30)	1. White noise (n = 30) 2. Silence (n = 30)	Alpha oscillations (EEG)			PI (NRS), PU (NRS)	*a* path
Lu 2023^57^	Exp/Heat	HV	Cross-over/NR	28	Music listening: 1. Liked music (n = 28) 2. Disliked music (n = 28)	Silence (n = 28)	Right PreCG/PoCG, left Putamen, left cerebellum (fMRI)			PI (NRS), PU (NRS)	True mediation
Maidhof 2025^62^	Exp/Cold P	HV	Cross-over/R	61	Music listening: 1. Participant-selected music (n = 61) 2. Researcher-selected music (n = 61)	Sound of lapping water (n = 61)	Stress (VAS), music-induced emotions (Likert)	Sex assigned at birth, music listening style (empathizer vs systematizer)		PI (VAS), PT	Mediation, Moderation
Mitchell 2006^64^	Exp/Cold P	HV	Cross-over/NR	54	Music listening: 1. Preferred music (n = 54) 2. Relaxing music (n = 54)	White noise (n = 54)		“Gender”		PI (VAS), PT, PU (MPQ)	Moderation
Mitchell 2008^65^	Exp/Cold P	HV	Cross-over/NR	80	Music listening (n = 80)	1. Art viewing (n = 80) 2. Silence (n = 80)	State anxiety (STAI), perceived control over pain (VAS), distraction (VAS)		Music listening frequency (Likert scale), knowledge of lyrics, music-evoked feelings	PI (VAS), PT	*a* path, prediction
Ortega 2019^67^	Clin	Adults, nasal bone fracture reduction	Parallel/R	36	Music listening (n = 17)	Silence (n = 19)	HR, SBP, DBP, state anxiety (STAI)			PI (VAS), pain memory (VAS)	*a* path
Pan 2025^68^ Experi-ment 1	Exp/Heat	HV	Parallel/R	80	1. Drumming only (n = 20) 2. Music listening only (n = 20) 3. Drumming and listening (n = 20)	Silence (n = 20)		Active music engagement		PI (NRS), PU (NRS)	Experimental manipulation
Pan 2025^68^ Experiment 2	Exp/Heat	HV	Parallel/R	66	1. Drumming, in-phase synchrony (n = 22) 2. Drumming, antiphase synchrony (n = 22) 3. Drumming, asynchrony (n = 22)		Sensorimotor synchronization			PI (NRS), PU (NRS)	Experimental manipulation
Pan 2025^68^ Experiment 3	Exp/Heat	HV	Parallel/R	78	1. Drumming, in-phase synchrony (n = 26) 2. Drumming, antiphase synchrony (n = 26) 3. Drumming, asynchrony (n = 26)		Neural mechanisms (EEG)			PI (NRS), PU (NRS)	Experimental manipulation
Pando-Naude 2019^69^	Clin	Adults, fibromyalgia	Cross-over/R	40	Music listening (n = 40)	Pink noise (n = 40)	Resting-state functional connectivity: angular gyrus, posterior cingulate cortex, precuneus, amygdala, middle frontal gyrus (fMRI)	Age		PI (NRS), PU (NRS)	*a* and *b* path, moderation
Perlini 1996^70^	Exp/Mech P	HV	Parallel/R	90	Music listening: 1. Choice, most-preferred (n = 15) 2. Choice, least-preferred (n = 15) 3. No choice, preferred (n = 15) 4. No choice, least-preferred (n = 15)	Silence: 1. Choice (n = 15) 2. No choice (n = 15)		Music choice, music preference		PI (NRS)	Moderation
Roy 2008^74^	Exp/Heat	HV	Cross-over/R	18	Music listening: 1. Pleasant music (n = 18) 2. Unpleasant music (n = 18)	Silence (n = 18)	Valence (NRS), arousal (NRS), mood (POMS)			PI (NRS), PU (NRS)	*a* and *b* path
Roy 2012^73^	Exp/Electr S	HV	Cross-over/R	30	Music listening: 1. Pleasant-stimulating music (n = 30) 2. Pleasant-relaxing music (n = 30) 3. Unpleasant-stimulating music (n = 30)	Silence (n = 30)	Descending pain modulation pathways (spinal nociceptive activity) (RIII reflex), ANS activity (SCR), valence (SAM)			PI (VAS), PU (VAS)	Experimental manipulation
Seminowicz 2019^78^	Exp/Heat	HV	Cross-over/R	21	Forced awakenings before music listening: 1. Rewarding music (n = 21) 2. Neutral music (n = 21)		Nucleus accumbens activation, nucleus accumbens connectivity with anterior midcingulate cortex (fMRI), emotional responses to music (GEMS-9)			PI (NRS), PU (NRS)	*a* path
Shim 2017^79^	Clin	Adults, uro-dynamic study	Parallel/R	148	Music listening (n = 74)	Silence (n = 74)		“Gender”		PI (VAS)	Moderation
Silvestrini 2011^80^	Exp/Cold P, Electr S	HV	Cross-over/R	20	Music listening: 1. Pleasant music (n = 20) 2. Unpleasant music (n = 20)	1. Silence (n = 20) 2. Auditory attention task (n = 20)	Emotions, attention (DES), distraction (DES), NFR	“Gender”		PI (NRS), PTh, PT, PU (NRS)	*a* path, moderation
Tollabzadeh 2023^81^	Clin	Adults, cancer	Parallel/R	54	Music listening (n = 25)	Counseling session (n = 29)	Salivary cortisol, anxiety (BAI), stress (PSS)			PI (MPQ), PU (MPQ)	*a* path
Van der Valk Bouman 2024^84^	Exp/Cold P	HV	Parallel/R	548	Music listening: 1. Urban (n = 117) 2. Electronic (n = 121) 3. Classical (n = 109) 4. Rock (n = 98) 5. Pop (n = 103)				Music genre (overall genre preferences and genre against pain)	PI (NRS), PT, PU (NRS)	Experimental manipulation
Vander Valk Bouman 2026^85^	Exp/Electr S	HV	Parallel/R	80	Music listening: 1. One min (n = 19) 2. Five min (n = 20) 3. Twenty min (n = 21)	No music (n = 20)	HRV, anxiety (STAI-6), valence (SAM), arousal (SAM),	Duration of music		PI (NRS), PU (NRS), PT, PTh	Experimental manipulation, mediation
Weinstein 2016^89^	Exp/Mech P	HV	Cross-over/NR	213 (n = 107 with PTh data)	Singing: 1. Small choir (20–80 participants) 2. Large choir (232 participants)		Positive affect (PANAS), negative affect (PANAS), feelings of inclusion (IOS), social connectedness (Likert scale)			PTh	*a* path
Werner 2023^90^	Exp/Mech P	HV	Cross-over/R	59	Music listening: 1. With tapping (n = 59) 2. Without tapping (n = 59)	Silence: 1. With tapping (n = 59) 2. Without tapping (n = 59)	Attention (task engagement), valence (NRS)	Task condition (active vs passive), music condition	Music preference, familiarity with music	PI (NRS), PU (NRS)	Experimental manipulation moderation, prediction
Wilson 2024^91^	Exp/Mech P, heat, cold P	Adults, fibromyalgia (FM)	Cross-over/R	109 (n = 39 FM; n = 70 HC)	Music listening: 1. Favorite music (n = 109) 2. Meditative music (n = 109)	White noise (n = 109)			Negative affect (PANAS), positive affect (PANAS), anxiety (PROMIS), depression (PROMIS), sleep disturbance (PROMIS), pain catastrophizing (PCS)	PTh, PT, temporal summation of pain, conditioned pain modulation (cold pressure task)	Prediction
Wright 2010^92^	Exp/Cold P	HV	Cross-over/R	23	Music listening: 1. Adventure video + classical (n = 75) 2. Adventure video + heavy metal (n = 75) 3. Romantic video + classical (n = 75) 4. Romantic video + heavy metal (n = 75) 5. Classical music (n = 75) 6. Heavy metal music (n = 75)	1. Adventure video (n = 75) 2. Romantic video (n = 75) 3. Silence (n = 75)	Mood (POMS), workload (NASA-TLX)	Age, sex assigned at birth		PI (NRS), PTh, PT	Experimenntal manipulation, moderation
Yi 2025^93^	Exp/Heat S	HV	Cross-over/R	60	Listening to preferred music at: 1. Spontaneous production rate (SPR) (n = 60) 2. SPR+15% (n = 60) 3. SPR-15% (n = 60)	Silence (n = 60)	Biological rhythm entrainment			PI (VAS)	Experimental manipulation
Zhang 2023^95^	Exp/Mech P	Adults, myofascial pain syndrome	Cross-over/NR	15	Music listening (n = 15)	Silence (n = 15)	Neural activity in BA6, BA9, BA10 and BA46 (fNIRS)			PI (VAS)	*a* path
Zhao 2009^96^	Exp/Heat	HV	Cross-over/R	20	Music listening: 1. Happy melody (n = 20) 2. Sad melody (n = 20)	1. Lecture (n = 20) 2. Silence (n = 20)	Happy-sad (MARS)			PI (NRS), PT, PU (SF-MPQ)	Experimental

* Unclear if gender identity or sex assigned at birth.

† Experimentally manipulated.

ACC = anterior cingulate cortex; ANS = autonomic nervous system; ASI-16 = Anxiety Sensitivity Index; ASI-R = Anxiety Sensitivity Index Revised; BAI = Beck Anxiety Inventory; BA6 = Brodmann Area 6; BA9 = Brodmann Area 9; BA10 = Brodmann Area 10; BA46 = Brodmann Area 46; BMRQ = Barcelona Music Reward Questionnaire; BP = blood pressure; CEQ = Credibility/Expectancy Questionnaire; Clin = clinical pain; DBP = diastolic blood pressure; DES = Differential Emotional Scale; EEG = electroencephalogram; EKG = electrocardiogram; EHS = Elkins Hypnotizability Scale; ELISA = enzyme-linked immunosorbent assay; Exp = experimentally induced pain; fMRI = functional magnetic resonance imaging; fNIRS = functional near-infrared spectroscopy; rs-fMRI = resting-state functional magnetic resonance imaging; GEMS = Geneva Emotional Musical Scale; GMSI = Goldsmith's Musical Sophistication Index; HC = healthy controls; HMGB-1 = High Mobility Group Box 1; HR = heart rate; HRV = heart rate variability; HV = healthy volunteers; IL-6 = interleukin-6; IOS = Inclusion of Other in Self Scale; MAP = mean arterial pressure; MARS = Multiple Affective Rating Scale; MPQ = McGill Pain Questionnaire; NASA-TLX = NASA-Task Load Index; NFR = nociceptive flexion response; NR = nonrandomized; NRS = numeric rating scale; PANAS = Positive and Negative Affect Schedule; PASAT = Paced Auditory Serial Addition Test; PASS-20 = Pain Anxiety Symptom Scale Shorty Form 20; PCS = Pain Catastrophizing Scale; PGIC = Patient Global Impression of Change Score; PI = pain intensity; POMS = Profile of Mood States; PT = pain tolerance; PTh = Pain threshold; PROMIS = Patient-Reported Outcomes Measurement Information System; PU = pain unpleasantness; QST = quantitative sensory testing; R = randomized; Right PreCG/PoCG = right precentral gyrus and postcentral gyrus; RIII reflex = RIII nociceptive flexion reflex; RR = respiratory rate; SAM = Self-Assessment Manikin; SBP = systolic blood pressure; SCR = skin conductance response; SF-MPQ = Short Form—McGill Pain Questionnaire; SMAP = Short Measure of Adult Playfulness; SPCS = Situational Pain Catastrophizing Scale; SPR = spontaneous production rate; STAI = Spielberger State-Trait Anxiety Inventory Form; 1 = state; 2 = trait; STAI-S = State-Trait Anxiety Inventory-State; STAI-T = State-Trait Anxiety Inventory-Trait; STICSA = State-Trait Inventory for Cognitive and Somatic Anxiety; TAU = treatment as usual; VAS = Visual Analog Scale; VRS = Verbal Rating Scale.

## 3. Results

### 3.1. Characteristics of included studies

**Figure 2.**
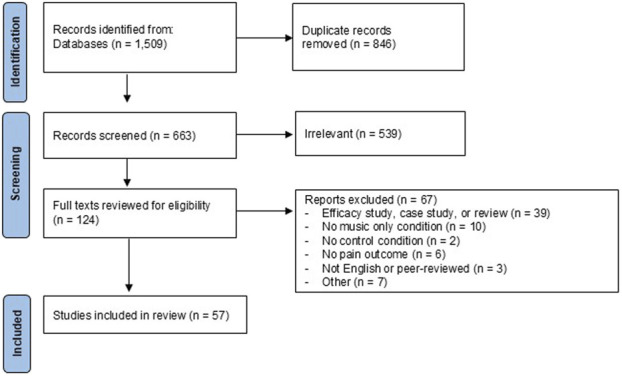
Flow diagram.

The search yielded 663 unique records. Following screening, 57 studies were included in the review (Fig. 2). Of these, 34 (60%) explicitly aimed to investigate mechanisms, moderators, or predictors. The majority (74%) employed experimental pain models. No studies involved pediatric populations. The studies were conducted across 20 countries. Sample sizes ranged from 12 to 200 participants. Nearly all studies (93%) focused on listening to prerecorded music; 4 examined active music-making, one of which tested an MBI delivered by a music therapist. Table 2 summarizes the characteristics of the included studies, Table 3 details the MBIs in clinical studies, and Table 4 details the music stimuli in experimental studies.

**Table 3:** Characteristics of music-based interventions in clinical trials.

Author (y)	Music condition(s)	Descriptions	Music selected by:	Interventionist/person who offered the music	Session format	Number/length of sessions
Anglin 2021^3^	Music listening	Participants' self-selected song on cellular device	Participant	N/A	Individual	1/duration of procedure
Bradt 2024^15^	Group music therapy	MT protocol that included active and receptive music experiences	N/A	Music therapist	Individual	6/45–60 min
Çift 2020^20^	Music listening	1. Turkish art music 2. Western classical music 3. Patient-preferred music (no further music details)	1 and 2. Researcher 3. Participant (limited set of options)	Research staff	Individual	1/duration of procedure
Deng 2022^24^	Music listening	Participants' selected music from a limited set of 40 songs across 4 genres (classical, light, retro, popular)	Participant (limited set of options)	Research staff	Individual	1/30 min
Du 2022^27^	Music listening	Relaxation music (8–150 Hz, 50–70 dB)	Researcher	Research staff	Individual	7/30 min
Goldfine 2025^39^	Music listening	Participant-preferred music on Spotify	Participant	N/A	Individual	1/10 min
Kavak Akelma 2020^50^	Music listening	Participants' preferred music (own music selection)	Participant	Research staff	Individual	1/15 min
Linnemann 2015^56^	Music listening	Participants' preferred music (eg, radio, playlists, etc.)	Participant	NA	NA	14 days/monitored daily music listening
Ortega 2019^67^	Music listening	Slow-paced music (60–80 BPM) (QR code to Spotify list provided)	Researcher	Research staff	Individual	1/from 10 min before through 10 min after procedure
Shim 2017^79^	Music listening	Participants' preferred music (no further details)	Participant	NA	Individual	1/duration of procedure
Tollabzadeh 2023^81^	Music listening	Participants' preferred music (from list of classical, pop, Iranian, meditation music)	Participant (limited set of options)	Research staff	Individual	16 (8 wk)/20 min

**Table 4:** Characteristics of music stimuli in experimental pain studies.

Author (y)	Music condition(s)	Descriptions	Music selected by:	Length of music excerpt(s)	No. of music trials/music stimulus exposure
Abrahan 2026^1^	Music listening 1. Pleasant-high arousal 2. Unpleasant-high arousal 3. Neutral—low arousal	27 musical excerpts (9 pleasant-activating; 9 unpleasant-activation; 9 neutral-nonactivating) were selected from 2 prior studies	Researcher	6–14 s (looped to 14–19 s during the experiment)	27 trials (18 with pain + 9 without pain)
Arican 2025^5^	Music listening	1. Music-only 2. Same Music + attention to music 3. Same Music + attention to pain Mozart's Divertimento in E-Flat Major, K. 563, II. Adagio and III. Menuetto: Allegretto—Trio pieces	Researcher	Music was started before participant entered the room and played throughout the experiment	1
Basiński 2021^10^	Music listening: 1. Arousal music 2. Valence music 3. Depth music	Unfamiliar music selected for attributes of high arousal, valence, or depth (list of music pieces and audio files provided in publication)	Researcher	Duration of hand submersion (cold pressor test)	3 blocks of 8 stimulus trials each
Becker 2025^12^	Music listening: 1. Self-chosen music 2. Researcher-chosen music	1. Participant-selected 10 tracks from their own music selection aimed at reducing pain 2. Classical music playlist selected by researchers (list is provided in the publication)	Participant Researcher	20 min	2
Chai 2020^16^	Music listening	Unwind music app (participants selected 1 track from 5 instrumental tracks)	Participant	10 min	1 QST session
Choi 2018^17^	Music listening	Traditional Korean folk song	Researcher	Unclear/likely for the duration of hand submersion during cold pressure task	1
Choi 2022^18^	Music listening	Traditional Korean folk song	Researcher	Unclear/likely for the duration of hand submersion during cold pressure task	1
Colebaugh 2023^21^	Music listening: 1. Relaxing music 2. Favorite music	1. Unwind app, relaxation tracks (limited set of options) 2. Participants' favorite music	1. Participant 2. Participant	Approx 20 min	2 QST sessions
Dobek 2014^25^	Music listening	Participants' favorite music	Participant	215 s	Spinal cord: 4 Brain: 3
Dunbar 2021^28^	Experiment 1: 1. Music listening without movement 2. Music listening with head nods Experiment 2: 1. Music listening with head nods 2. Music listening with foot taps	Upbeat contemporary dance music (no further details)	Researcher	13 min	2
Ernberg 2020^29^	Music listening: 1. Black metal music 2. Classical music	1. Extreme subgenre of heavy metal music (Hvite Krists Dod by Satyricon) 2. Pleasant and relaxing classical music (Spiegel im Spiegel by Arvo Pärt)	Researcher	From time participant stepped into room until pain subsided	2
Evers 2024^30^	Music listening: 1. Pleasant music 2. Unpleasant music	1. Classical orchestral music (Johannes Brahms, 3rd Symphony, op. 90, 3rd movement) 2. Contemporary classical music (Krzysztof Penderecki, Threnos, 1960/6)	Researcher	5 min	2 QST sessions
Finlay 2016^33^	Music listening: 1. Happy 2. Sad 3. Relaxing	Participants' favorite music based on perceived valence	Participant	Duration of cold pressor test	3
Garcia 2016^35^	Music listening: 1. Motivating music 2. Relaxing music	Participants' favorite music based on perceived valence	Participant	Duration of cold pressor test	2
Garza-Villarreal 2012^36^	Music listening: 1. Mozart 1 2. Mozart 2	Pleasant, unfamiliar, low-arousal Mozart string compositions (String Quartet No. 1 in G major, K. 80/73 f (Adagio); Divertimento in E-flat major, K. 563 (Adagio))	Researcher	5 min	2 blocks of 8 stimuli each
Hekmat 1993^42^	Music listening: 1. Preferred music 2. Nonpreferred music	Based on participant ratings of instrumental selections in 4 genres (classical, jazz, rock, country)	Participant (limited set of options)	Duration of cold pressor test	2
Howlin 2021^45^	6 conditions combining 2 types of music (relaxing music vs motivating music) with 3 levels of choice (no choice vs perceived choice from 2 songs vs perceived choice from 4 songs)	Unfamiliar instrumental music: Relaxing music: low tempo (60–80 bpm), low levels of arousal and gentle rhythms Motivating music: high tempo (100–110 bpm), high levels of arousal and danceable rhythms A table with all music pieces is included in the publication	Researcher	Duration of cold pressor test	6
Hsieh 2014^47^	Music listening	Participants' favorite music with no associated visual memories (eg, music video)	Participant	4 min	3
Johnson 2020^48^	Music listening: 1. Music with relaxation suggestions 2. Music alone	Excerpt of relaxing classical music (Fantasia on a Theme of Thomas Tallis by Ralph Vaughn-Williams)	Researcher	Intervention began 10 min before immersion 1. 3 min relaxation suggestions + 12 min music 2. 15 min music	1
Kenntner-Mabiala 2007^51^	6 music listening conditions combining 3 music tempi (slow vs medium vs fast) with 2 modes (major vs minor)	Classical piano sonata music digitally modified for tempo (slow, medium, fast) and mode (major/minor) (4 sonata movements digitally manipulated into 24 excerpts varying in tempo[46, 60, 95 BPM] and mode[major/minor])	Researcher	80 s	24
Lad 2022^53^	Music listening: 1. Music alone 2. Music with tactile vibrations	Participants selected a liked song from list 1 and a disliked song from list 2 (lists based on arousal and valence)	Participant (limited set of options)	Duration of force pain induction	2 blocks of 6 stimulus trials each
LiKamWa 2022^55^	1. Singing 2. Music listening	Participants' preferred music (from a list of 16 songs across a range of genres)	Participant (limited set of options)	20 s prior and through duration of hand submersion (cold pressor test)	3 QST sessions
Lu 2019^58^	Music listening	Participants' favorite music	Participant	2 min prior and through duration of laser stimulus trials	16
Lu 2023^57^	Music listening: 1. Liked Music 2. Disliked Music	1. Participants' preferred music 2. Participants' most disliked music Participants were paired so their most liked music was partner's most disliked music	Participant (limited set of options)	5 min prior and through duration of laser stimulus trials	20
Maidhof 2025^62^	Music listening 1. Participant-selected music 2. Researcher-selected music	1. Participant-preferred relaxing song (from own music collection) 2. Researcher-selected relaxing song (Carnelian from New World Music's An introduction to Music to Relax, inspire and Uplift You, Vol. 3)	Participant, Researcher	Duration of hand submersion (cold pressor test)	2
Mitchell 2006^64^	Music listening: 1. Preferred usic 2. Relaxing music	1. Participants’ preferred music 2. Relaxing music (Carnelian from New World Music's An Introduction to Music to Relax, Inspire and Uplift You, Vol. 3)	1. Participant 2. Researcher	Duration of hand submersion (cold pressor test)	3
Mitchell 2008^65^	Music listening	Participants' preferred music (own music collection)	Participant	Duration of hand submersion (cold pressor test)	3
Pan 2025^68^ Experiment 1	Music listening 1. Listening only 2. Drumming only 3. Listening + drumming	2 percussion excerpts (90 and 120 BPM) Drumming-only condition used visual metronome cues (muted video) without auditory stimuli	Researcher	2.5 min/percussion excerpt	2
Pan 2025^68^ Experiment 2	Music listening 1. In-phase synchrony 2. Antiphase synchrony 3. Asynchrony	In all 3 groups participants listened to percussion excerpts (90 and 120 BPM) 1. Audio + visual aligned; synchronized drumming 2. Audio + visual 180° shifted; antiphase drumming 3. Audio + visual mismatched tempo; asynchrony drumming	Researcher	2.5 min	2
Pan 2025^68^ Experiment 3	Music listening 1. In-phase synchrony 2. Antiphase synchrony 3. Asynchrony	In all 3 groups participants listened to percussion excerpts (90 and 120 BPM) 1. Audio + visual aligned; synchronized drumming 2. Audio + visual 180° shifted; antiphase drumming 3. Audio + visual mismatched tempo; asynchrony drumming	Researcher	2.5 min	2
Perlini 1996^70^	Music listening: 1. Choice, most-preferred 2. Choice, least-preferred 3. No choice, preferred 4. No choice, least-preferred	Participants' preferred and least preferred music from 6 Billboard top hits (R&B, adult contemporary, classical, country, alternative, dance/rap)	Participant (limited set of options)	1 min	3
Roy 2008^74^	Music listening: 1. Pleasant music 2. Unpleasant music	1. Music with high valence/medium arousal (Love and Happiness [Ernst Ranglin], William Tell Overture [Rossini], French Cancan [Canissimo]) 2. Music with low valence/medium arousal. (Pendulum Music [Sonic Youth/Steve Reich], The Threshold of Deafening Silence [Paul Dolden], Fascicles [The Thirteen Ghosts])	Researcher	15 min blocks created by combining 5-min audio excerpts	8
Roy 2012^73^	Music listening: 1. Pleasant-stimulating music 2. Pleasant-relaxing music 3. Unpleasant-stimulating music	1. Classical music with high valence/high arousal: William Tell Overture (Rossini); Hungarian Dances No. 5 (Brahms); Russian Dance (The Nutcracker, Tchaikovsky); French Cancan; Love and Happiness (Ernest Ranglin); Trumpet Concerto, 3rd movement (Haydn) 2. Classical music with high valence/low arousal: Surfin’ (Ernest Ranglin); Histoires sans paroles (Harmonium); The Blue Danube (Johann Strauss); Twenty Eight Parallel (Vangelis); Symphony No. 6 “Pastorale” (Beethoven); Novio (Moby) 3. Contemporary classical music with low valence/high arousal: Demon Sanctuary (John Zorn); Pendulum Music (Steve Reich/Sonic Youth); Fascicles (Derek Bailey and Thurston Moore); Below the Walls of Jericho (Paul Dolden); Caught in an Octagon of Unaccustomed Light (Paul Dolden)	Researcher	1 min	26
Seminowicz 2019^78^	Forced awakenings before music listening: 1. Rewarding music 2. Neutral music	1. Participants' preferred rewarding music (high valence) 2. Billboard top hits with neutral valence	Participant	51 s	3 runs of 10 trials each
Silvestrini 2011^80^	Music listening: 1. Pleasant music 2. Unpleasant music	1. Classical music with high valence (Bach, Brandenburg Concertos nos. 2 and 3, 1st and 3rd movements; Mozart, Eine Kleine Nachtmusik, Allegro and Rondo; or Bizet, Symphony in C Major, 4th Movement) 2. Contemporary classical music with low valence (Penderecki, Symphony no. 1, Dynamis II; Gyorgy Ligeti, Concerto for Cello and Orchestra; Pierre Boulez, Notations II for Orchestra)	Participant (limited set of options)	40 min	30–40 electrical stimulations for nociceptive flexion response; 1 cold pressor test
Van der Valk Bouman 2024^84^	Music listening: 1. Urban 2. Electronic 3. Classical 4. Rock 5. Pop	1. Hip Hop, Rap, R&B 2. Dancehall, Techno, House, Electronica 3. Classical symphony 4. Punk rock, hard rock, Indie rock, modern rock 5. Pop, Indie pop, alternative pop	Researcher	7 min blocks created by combining 45-s audio excerpts	1
Van der Valk Bouman 2026^85^	Music listening: 1. 1 min 2. 5 min 3. 20 min	Participants preferred music (Spotify)	Participants	1. 1 min 2. 5 min 3. 20 min	1
Weinstein 2016^89^	Singing: 1. Small choir (20–80 participants) 2. Large choir (232 participants)	Rehearsing popular songs	N/A	90 min	1
Werner 2023^90^	Music listening: 1. with tapping 2. without tapping	Instrumental music with neutral valence at approximately 60 dB sound pressure. All music pieces are listed in the publication	Researcher	30 s	20
Wilson 2024^91^	Music listening: 1. Favorite music 2. Meditative music	1. Participants' favorite music 2. Unwind app	1. Participant 2. Researcher	Length of QST session, approx. 20 min	2 QST
Wright 2010^92^	Music listening: 1. Adventure video + Classical 2. Adventure video + Heavy Metal 3. Romantic video + Classical 4. Romantic video + Heavy Metal 5. Classical music 6. Heavy Metal music	1, 3, 5: Stabat Mater Movement XII, Quando Corpus-Amen by Giovanni Pergoles 2, 4, 6: “Drag the Waters” by Panter	Researcher	5 min	6
Yi 2025^93^	Music listening: 1. SPR (spontaneous production rate) 2. SPR+15% 3. SPR-15%	Participants chose a preferred genre from a limited list (popular, classical, dance, international). Excerpts were presented at 3 tempi relative to the participant's SPR (SPR, +15%, −15%)	Participants (from limited set)	194 s–252 s	9
Zhang 2023^95^	Music listening	Soothing synthetic music with frequencies of 8–150 Hz and an intensity range of 50–70 dB	Researcher	5:30 min	3
Zhao 2009^96^	Music listening: 1. Happy melody condition 2. Sad melody condition	Chinese violin concerto ‘Butterfly Lovers’: 1. Fast-tempo (156 bpm), major-mode excerpt 2. Slow-tempo (44 bpm) minor-mode excerpt	Researcher	5 min	2

fMRI = functional magnetic resonance imaging; QST = quantitative sensory testing; CPT = cold pressor task.

### 3.2. Presentation of the results

Results are organized by candidate mechanism type—physiological, psychological, neural, social, music-specific, and demographic—and by study design: (1) experimental manipulation of candidate variables, (2) mediation studies, (3) moderation studies, and (4) prediction studies. For mediation studies, we grouped studies that used partial mediation analyses (*a* path only, *b* path only, *a* and *b* paths but no true mediation analysis) vs those that conducted a true mediation analysis (a*b path) (Fig. 1).

Information related to study characteristics (Tables 2–4) and statistical findings (supplemental digital content, materials, http://links.lww.com/PR9/A422) are included for all studies. However, only studies deemed sufficiently powered are discussed in detail in the results section. Underpowered studies are referenced but not discussed. The interpretation of the findings is based on adequately powered studies to avoid basing conclusions on studies with potentially spurious findings. To determine whether a study was sufficiently powered, we reviewed sample size calculations when reported. When absent, we used G*Power^31^ to estimate the minimum required sample size needed to detect a significant effect, assuming a medium effect size and accounting for the study design and planned analyses. Exceptions were made for (1) studies comparing pleasant vs unpleasant music, where larger effect sizes are typically expected (thus requiring smaller sample sizes) and (2) brain imaging studies, which typically involve smaller samples due to cost. Although this approach has limitations, it provided a transparent and consistent framework for selecting studies to review.

### 3.3. Physiological candidate variables

Eleven studies assessed physiological variables. Four were not sufficiently statistically powered.^14,48,85,92^

#### 3.3.1. Mediation studies

##### 3.3.1.1. Partial mediation (*a* path)

Kenntner-Mabiala^51^ (N = 38 healthy volunteers) investigated whether physiological arousal induced by different music tempi (slow, moderate, and fast) affected pain perception. The fastest tempo produced the largest increase in respiratory rate (*P* = 0.011) but not heart rate (*P* = 0.097) and led to the highest pain ratings (*P* < 0.016), but only in female participants. Kavak Akelma^50^ (N = 117 adults with hernia) compared the effects of favorite music listening for 15 minutes before surgery to standard care on surgical pain. Music listening reduced heart rate, systolic blood pressure, and diastolic blood pressure more than standard care (*P*'s = 0.001) but had no significant effect on pain intensity ratings. Becker^12^ examined the effects of participant-selected vs researcher-selected music vs a podcast on heart rate variability and pain perception of 84 healthy volunteers during electric pain stimulation. Heart rate variability analysis revealed more parasympathetic activity with the researcher-selected music and a higher heart rate with the participant-selected music than with the control intervention (*P*'s < 0.001). Participant-selected music improved pain intensity, unpleasantness, tolerance, and threshold more than the podcast (*P*'s < 0.05), whereas researcher-selected music only improved pain threshold (*P* = 0.018). The effects of participant- and researcher-selected music on the pain outcomes did not differ except for pain intensity, which was reduced most with participant-selected music (*P* = 0.002).

In an observational study, Linnemann^56^ (N = 30 women with fibromyalgia) hypothesized that the hypothalamic-pituitary-adrenal axis and autonomic nervous system activity would mediate the pain-reducing effects of music. Using ecological momentary assessment and saliva collection 4 times per day for 14 days, they examined the effects of music listening in daily life on pain and stress. Music listening was not associated with changes in salivary cortisol (*P* = 0.488) or salivary alpha-amylase (*P* = 0.938). Music listening increased perceived control over pain (*P* < 0.001) but did not affect pain intensity (*P* = 0.32).

Two studies^73,80^ investigated the effects of music on the nociceptive flexion reflex, a muscle withdrawal reflex that acts as a measure of nociceptive transmission in the spinal cord. Silvestrini^80^ (N = 20 healthy volunteers) investigated the pain stimulus intensity required to trigger a nociceptive flexion reflex during pleasant music (Western classical music) vs unpleasant music (dissonant excerpts of contemporary music) vs silence vs auditory attention task (a pitch discrimination task). Participants experienced each condition for 2 minutes before the cold pressor tasks. No significant effects were found, possibly due to a lack of power (despite comparing pleasant with unpleasant music), although they showed a trend towards a higher threshold for pleasant music compared with other conditions. Roy^73^ (N = 30 healthy volunteers) exposed participants to transcutaneous electrical stimulations while listening to music excerpts with varying valence and arousal levels (ie, pleasant, stimulating vs pleasant, relaxing vs unpleasant, stimulating vs silence). An effect was found for pleasant vs unpleasant music on nociceptive flexion reflex amplitude (*P* = 0.038).

##### 3.3.1.2. Partial mediation (*a* path and *b* path)

Deng^24^ (N = 160 women undergoing breast cancer surgery) reported that listening to participant-preferred music reduced pain intensity and inflammatory biomarkers (interleukin-6 and Plasma High Mobility Group Box 1) more so than SC (*P* = 0.001). Reductions in these cytokines were significantly correlated (*P* < 0.01) with reductions in pain intensity.

### 3.4. Psychological candidate variables

Twenty-six studies studied psychological candidate mechanism variables. Nine studies were not sufficiently powered.^5,35,39,47,48,62,67,70,81^

#### 3.4.1. Experimental manipulation

Three studies experimentally manipulated psychological variables. Two studies by Howlin et al manipulated cognitive agency through perceived choice in music.^45,46^ In the first of these^45^ (N = 51 healthy adults), participants with greater music choice reported more pain tolerance and lower pain intensity during a cold pressor task than did those with less music choice (*P's* < 0.05). Importantly, pain reduction was amplified among participants who reported more enjoyment. Similarly, in a second study^46^ (N = 286 adults with acute pain) that used a 2 × 2 design to examine music choice (yes vs no) and music complexity (high vs low), perceived music choice resulted in lower pain intensity ratings than did no choice (*P* < 0.05). However, in both studies, choice did not significantly affect pain unpleasantness. Neither the main effect of music complexity nor the Choice × Complexity interaction significantly predicted pain intensity.

Werner^90^ (N = 59 healthy adults) manipulated sensorimotor synchronization through tapping/no tapping alongside music or silence, examining its effects on pain intensity during an experimental pressure pain task. The analgesic effect of music tapping was significantly greater than all other conditions (*P*'s < 0.001).

#### 3.4.2. Mediation studies

##### 3.4.2.1. Full mediation

Bradt^15^ (N = 92 adults with advanced cancer) was the only one of the 24 identified studies to test true mediation. Participants were randomized to 6 individual music therapy or social attention control sessions. Mood, general anxiety, and pain-related self‐efficacy were examined as potential mediators of the effect of music therapy on pain intensity and pain interference. The results indicated that self-efficacy was a significant mediator (intensity: *a* × *b* = 0.79, 95% CI 0.01–1.82; interference: *a* × *b* = 1.16, 95% CI 0.02–2.51). Temporal precedence of mediators tested strengthened the findings about self-efficacy as a potential mediator.

##### 3.4.2.2. Partial mediation (*a* path)

###### 3.4.2.2.1. Anxiety

Six studies examined anxiety as a potential mediator. Two were not sufficiently powered.^67,81^

Mitchell^65^ (N = 80 healthy adults) used a within-subjects design to compare the effects of self-selected, preferred music vs art viewing vs silence on pain intensity, pain tolerance, and state anxiety during a cold pressor task. Participants reported lower state anxiety during the preferred music condition than during both the art viewing and silence conditions (*P*'s < 0.001). Preferred music resulted in the greatest improvements in pain tolerance (*P* < 0.001) and pain intensity (*P* < 0.05).

Çift^20^ (N = 150) conducted an RCT on the effects of Turkish art music vs Western classical music vs patient-preferred music vs two silence conditions (with and without headphones) on pain intensity, anxiety, and satisfaction during extracorporeal shock wave lithotripsy. Participants in the 3 music groups (all delivered through headphones) reported lower anxiety and pain intensity than participants in the silence conditions (*P's* < 0.05). The patient-preferred music condition reduced anxiety more than the 2 other music conditions (*P*'s < 0.05).

Kavak Akelma,^50^ as discussed before, assessed state anxiety 1-hr before and 4 to 6 hours following hernia repair surgery. The music group reported greater pre-post reduction in anxiety (*P* = 0.001) than the standard care group, although the groups did not significantly differ in pain intensity.

Becker,^12^ previously discussed, reported greater anxiety-reducing effects of listening to participant-selected (*P* < 0.001) and researcher-selected (*P* = 0.028) music than a podcast, but no significant differences between the 2 music conditions.

###### 3.4.2.2.2. Positive/negative affect

Five studies examined mood/emotions as a potential mediator. Two were not sufficiently powered.^35,62^

Likamwa^55^ (N = 40) compared singing vs music listening vs silence on pain (intensity, threshold, tolerance) and affect (positive and negative) during a cold pressor task. Participants selected a preferred song from a researcher-provided list to be used for both the singing and music listening conditions. The 3 groups did not differ significantly on positive or negative affect following the cold pressor task. The singing condition was more effective in improving pain intensity, pain tolerance, and pain threshold than listening to music or silence.

Weinstein^89^ compared small group choir singing (n = 20–80 members; n = 45 completed pain testing) vs large group choir singing (n = 232 members; n = 62 completed pain testing) on pain threshold and affect (positive and negative). Pain was induced by pressure algometry before and after a 90-minute choir rehearsal. Participants in both groups experienced significantly increased positive affect (*P* < 0.001) and decreased negative affect (*P* < 0.001) after singing. The increase in positive (but not negative) affect was greater among the small group choir participants (*P* = 0.002), but this was not robust to additional statistical adjustments. Pain threshold showed a main effect of time. However, there was no significant main effect of group or Group × Time interaction.

Silvestrini,^80^ previously discussed, found that participants reported significantly more positive emotions (joy and happiness; *P*'s < 0.001) during a pleasant music condition than during unpleasant music, silence, and auditory attention task conditions. During the unpleasant music condition, participants reported significantly more negative emotions (anger, anxiety, and disgust; *P*'s < 0.05) than during the pleasant music or silence conditions. Both the pleasant music (*P*'s < 0.02) and auditory attention task (*P*'s < 0.05) conditions resulted in greater improvements in all pain outcomes than the unpleasant music and silence conditions.

###### 3.4.2.2.3. Distraction

Three studies, all previously discussed, examined distraction as a potential mediator. One additional study was not sufficiently powered.^5^ In the Likamwa study,^55^ most participants (83%) reported that singing was most distracting from pain, with a smaller number (18%) reporting music listening as most distracting; none reported that silence was most distracting. Singing was also found to be most effective in improving pain outcomes. There were no significant differences between music listening and silence for any of the pain outcomes.

Mitchell^65^ found that preferred music was significantly more distracting than art viewing or silence (*P's* < 0.001) and that art viewing was more distracting than silence (*P* < 0.05). Preferred music also resulted in greater improvements in pain outcomes than the other conditions (*P*'s < 0.05).

Participants in the Silvestrini study^80^ reported that, compared with silence, they were able to concentrate more intently on pleasant music, unpleasant music, and auditory attention task (*P*'s < 0.02). They also reported more attention to auditory attention task than pleasant music (*P* < 0.05), but no significant differences in ability to concentrate were found between the 2 music conditions. Both pleasant music and auditory attention task resulted in significantly better pain outcomes than unpleasant music or silence (*P*'s < 0.05).

###### 3.4.2.2.4. Perceived control over pain

The previously discussed Mitchell study^65^ found that participants reported more perceived control over pain in the preferred music condition than in the art viewing condition (*P* < 0.001) and silence condition (*P* < 0.001).

##### 3.4.2.3. Partial mediation (*a* path and *b* path)

Three studies, all with healthy adults, examined both *a* and *b* paths, but did not conduct full mediation analyses.

Colebaugh^21^ (N = 70) compared the effect of listening to favorite music vs listening to a music app delivering relaxing music vs white noise on catastrophizing as a potential mediator of treatment effects on quantitative sensory testing (QST)-based pain outcomes. Participants were asked to select 1 of 5 possible tracks of AI-generated relaxing music. Across all analyses, only the *a* path was statistically significant, such that the favorite music condition resulted in lower catastrophizing than the other 2 conditions (*P* < 0.05). The *b* paths (catastrophizing→pain outcomes) were examined with separate correlation analyses for each condition, but none were statistically significant, and the authors did not test whether the magnitude of the correlations differed between the conditions.

Finlay^33^ (N = 41) compared participant-provided happy and sad music to participant-provided relaxing music or a no music control condition on cold pressor pain intensity and tolerance. Both happy and relaxing music increased pain tolerance to a greater extent than sad music. Relaxing music resulted in lower anxiety than sad music and no music (*P*'s < 0.01). Happy and relaxing music also facilitated greater pain distraction and perceived pain control than sad music (*P*'s < 0.02). The *b* paths were examined with separate correlation analyses for each condition. For anxiety, only the happy and sad music resulted in significant correlations for pain intensity (*P*'s < 0.01) whereas, for pain tolerance, only happy music (*P* < 0.01) and no music (*P* < 0.05) were significant. For distraction, *b* paths were significant for all music conditions for pain tolerance (*P* < 0.01) but not intensity. For perceived pain control, all conditions had significant correlations for both pain outcomes (*P*'s < 0.01 and 0.05). However, the authors did not statistically test whether the magnitude of these correlations differed significantly between the conditions.

Ernberg^29^ (N = 20 women) compared the effects of classical music listening vs black metal music listening vs no music on pain intensity, pain duration, and pain quality, as well as on anxiety. Participants completed 3 sessions during which hypertonic saline was injected into the masseter muscle. The conditions did not significantly differ on ratings of anxiety. The *b* paths (only anxiety→peak pain intensity was tested) were examined with separate correlations for each experimental condition. All of these correlations were significant (*P's* < 0.02), but the authors did not test whether the magnitudes statistically differed between conditions.

##### 3.4.2.4. Moderated mediation

Bradt,^15^ previously discussed, tested for moderated mediation effects. Baseline measures of music reward, adult playfulness, treatment outcome expectancy, and baseline pain intensity and interference were examined as moderators of the extent to which pain self-efficacy mediated the effects of music therapy on pain intensity and pain interference. Only baseline pain interference emerged as a significant moderator, suggesting that self-efficacy played a larger role as a mediator of the effects of music therapy on both outcomes for those who reported more pain interference at baseline.

#### 3.4.3. Moderation studies

Four studies examined whether music's effects on pain vary as a function of baseline measures of psychological variables. Two were not sufficiently powered.^47,48^

Choi^17^ (N = 50 healthy adults) compared the effects of researcher-selected traditional Korean folk music vs news listening vs no sound on pain threshold, intensity, and unpleasantness in response to a cold pressor test. Moderator effects were not examined by testing the Moderator × Treatment Condition interaction effect. Instead, these investigators examined responses to music as a function of different levels of anxiety. They reported a significant effect for experimental condition among those endorsing lower but not higher baseline levels of anxiety sensitivity, with those endorsing lower levels evidencing larger reductions in pain during the music condition than the no sound control condition (*P*'s ≤ 0.002 for all 3 outcomes) and news condition (*P*'s range 0.004–0.030 for all 3 outcomes). For pain-related anxiety, only those with lower levels of anxiety experienced reduced pain intensity when listening to music compared with the other conditions. For the other pain outcomes, results for lower and higher levels of pain-related anxiety were similar.

Choi^18^ used the same approach as Choi^17^ to examine the possible moderating effects of the 4 anxiety measures used in their study. Participants endorsing lower levels of anxiety sensitivity showed larger between-condition effects on pain (favoring the music condition). However, the moderating effects of anxiety did not appear to be as strong for pain tolerance and pain unpleasantness. Only for pain intensity was the group effect significant for the lower anxiety participants (*P* = 0.032) but not for the higher anxiety participants.

#### 3.4.4. Prediction studies

Three studies tested psychological variables as predictors of music-induced pain relief. One was not sufficiently powered.^70^

Chai^16^ (N = 60 healthy adults) evaluated the effects of music, selected by participants from a limited number of tracks in a music app, on QST measures. Correlation analyses examined the associations between baseline pain-related catastrophizing, anxiety, and depression, and pain outcomes, but only the findings for catastrophizing were reported. Participants with higher baseline catastrophizing experienced less music-induced analgesia on 1 of the 3 pain outcomes (pain tolerance; *P* = 0.035).

The study by Wilson^91^ (n = 70 healthy adults; n = 39 adults with fibromyalgia) examined the effects of 2 music conditions (favorite music, “meditative” music) vs white noise, on 7 QST measures of pain sensitivity. Predictor analyses were conducted for these 2 groups separately for 6 potential predictor variables (negative affect, positive affect, depression, anxiety, sleep disturbance, and pain catastrophizing). A total of 28 analyses (ie, for 2 music conditions, 2 sample subgroups, 7 outcomes) were conducted for each of the 6 predictors, which translates to 168 analyses, substantially increasing the likelihood of false positives. Five percent of the associations tested were statistically significant at the 0.05 level.

### 3.5. Neural candidate variables

Nine studies employed neural data acquisition methods (fMRI, EEG, and fNIRS).

#### 3.5.1. Mediation studies

##### 3.5.1.1. Full mediation

Lu^57^ (N = 28 healthy participants) used painful laser stimuli delivered to the hand while participants listened to either their own preferred music or disliked music submitted by another participant. Compared with nonpreferred music, preferred music was associated with lower neural responses to nociceptive stimuli in a distributed set of regions including the right Pre- and Post-Central Gyri (PreCG/PoCG), Anterior Cingulate Cortex, Mid-Cingulate Cortex, bilateral putamen, left middle frontal gyrus, right precuneus, bilateral lingual gyrus, and left cerebellum (*a* path). Between-subject differences in activity for preferred vs nonpreferred music in the right PreCG/PoCG, left putamen, and left cerebellum correlated significantly with between-subject differences in pain ratings (*b* path). Finally, between-subject differences in activity for preferred vs nonpreferred music in the right PreCG/PoCG and left cerebellum significantly mediated the effects of preferred music on pain (*a* × *b* path).

Pan^68^ (N = 80, N = 66, N = 78 healthy volunteers) conducted 3 between-subjects experiments examining the effects of music listening, drumming, and perceived sensorimotor synchronization on pain ratings and laser-evoked EEG responses. In experiment 1, drumming along to percussion excerpts produced greater pain reduction than listening to the excerpts alone (*P* < 0.001). Drumming without accompanying excerpts was not superior to silence (*P* = 0.66). In experiment 2, participants drummed in either in-phase synchrony, antiphase synchrony, or asynchrony to visual drumming cues tied to the timing of a concurrent auditory beat. In-phase and antiphase synchrony led to greater reductions in pain intensity compared with the asynchronous condition (*P* < 0.001), while only the in-phase group showed significant reductions in pain unpleasantness (*P* = 0.002). Average between-subject changes in laser-evoked N2-amplitudes before and after in-phase drumming (experiment 3) partially mediated changes in pain intensity ratings in comparison to asynchronous drumming (*a* × *b* path).

##### 3.5.1.2. Partial mediation (*a* path only)

Dobek^25^ (N = 12 healthy adults) examined the impact of self-selected preferred music vs silence on brain and spinal cord fMRI activity in response to heat pain. Increased neural activity in response to heat during music relative to silence (*a* path) was observed across a broad network of brain regions. In the brainstem and spinal cord, music increased pain-related activity in the periaqueductal gray, dorsolateral pontine tegmentum, and left ventral horn of the spinal cord, while reducing it in the right dorsal horn and rostral ventromedial medulla. However, no *b* paths were examined, and regions showing reduced pain-related activity during music, which could reflect music's effects on pain-processing areas, were not assessed.

Lu^58^ (N = 30 healthy adults) employed the same design as their study^57^ described above, using EEG with painful laser stimuli and comparing preferred music, white noise, and silence. Music reduced pain unpleasantness compared with both controls and was rated as more relaxing, pleasant, and liked than white noise. P2 amplitudes were reduced in the music condition compared with silence, but there were no differences between music and white noise. During the pain anticipatory period (−207 to 0 milliseconds), prestimulus EEG oscillations in the alpha range, maximally recorded at central electrodes, were lowest during the music condition compared with silence and noise.

Seminowicz^78^ (N = 21 healthy adults) investigated whether self-selected preferred music vs neutral music modulated the nucleus accumbens (NAc) response to thermal pain. There was no significant difference in pain-related NAc activity between preferred and neutral music. However, there was decreased connectivity between the NAc and anterior midcingulate cortex and increased NAc-orbitofrontal cortex connectivity at pain onset during preferred vs neutral music.

Zhang^95^ (N = 15 individuals with chronic pain) measured pressure-evoked pain responses in the prefrontal cortex with fNIRS during soothing music and silence. Music reduced subjective pain ratings (*P* < 0.05) and the magnitude of pain-evoked oxyhemoglobin responses in dorsolateral prefrontal cortices (BA9/BA46) (*P* = 0.037 and 0.049, respectively), anterior prefrontal cortices (BA10) (*P* = 0.047) and premotor/supplementary motor areas (BA6) (*P* = 0.025) more than silence.

Finally, Du^27^ (N = 37 individuals with chronic pain) used fNIRS to evaluate standard care vs standard care plus 30 minutes of daily music listening. Music reduced pain ratings, heart rate, and pain-evoked hemodynamic responses in prefrontal regions and strengthened functional connectivity between bilateral dorsolateral prefrontal cortices (BA9/46) and frontopolar areas (BA10) (*a* paths).

##### 3.5.1.3. Partial mediation (*a* path and *b* path)

Two studies examined *a* and *b* paths without formally testing the *a* × *b* mediation term. Garza-Villareal^37^ (N = 22 individuals with fibromyalgia) assessed the impact of music listening on brain activity by recording resting-state fMRI (rs-fMRI) before and after listening to self-selected preferred music vs pink noise. Music increased local spontaneous brain activity in the left angular gyrus as measured by the functional amplitude of low-frequency fluctuations (*P* = 0.008; *a* path). In addition, changes in pain scores were negatively correlated with within-subject activity in the left angular gyrus between conditions (*b* path, *P* = 0.03) and positively correlated with increased functional connectivity at the group level between the left angular gyrus and the right precentral gyrus (*b* path, *P* = 0.02).

In a subsequent study^69^ (n = 20 adults with fibromyalgia, n = 20 healthy controls), the same team compared pre/post effects of music on activity in brain networks that are associated with fibromyalgia. At baseline, pain matrix–precuneus connectivity was generally higher in the fibromyalgia group than controls. After music, connectivity decreased in the fibromyalgia group but increased in controls. Subsequent analyses of pre/post effects of music in the fibromyalgia group found that the participants showing the greatest reductions in pain intensity ratings also showed the greatest reductions in connectivity between the pain matrix and the precuneus (*b* path).

### 3.6. Social candidate variables

Three studies examined social candidate variables. One was not sufficiently powered.^12^

#### 3.6.1. Mediation studies

##### 3.6.1.1. Full mediation (*a* × *b* coefficient)

Bradt,^15^ previously discussed, examined emotional support as a potential mediator of the effects of music therapy vs social attention control on pain outcomes. Emotional support did not emerge as a significant mediator.

##### 3.6.1.2. Partial mediation (*a* path)

Weinstein,^89^ previously discussed, examined feelings of inclusion and social connectedness as potential mediators of choir singing in smaller vs larger ensembles on pain threshold. Both groups reported significant increases in inclusion (*P's* < 0.01) and connectedness (*P's <* 0.001) time, with larger increases in social connectedness reported by the larger choir participants. However, there were no significant between-group differences in pain threshold, and the effects of changes in inclusion or connectedness on pain threshold were not directly tested.

### 3.7. Music-specific candidate variables

A total of 26 studies examined music-specific variables. However, 9 of these were not sufficiently powered.^29,30,70,78,80,84,85,92,96^

#### 3.7.1. Experimental manipulation

##### 3.7.1.1. Emotional valence

Six studies experimentally manipulated emotional valence,^49^ measured as felt pleasantness to unpleasantness, but 3 were not sufficiently powered.^30,78,96^ Roy,^73^ described previously, found that pleasant, relaxing music reduced pain ratings, whereas unpleasant music increased pain ratings during transcutaneous electrical stimulation (*P* < 0.001). Abrahan^1^ (N = 28 healthy adults) compared the effects of pleasant-high arousal vs unpleasant-high arousal vs neutral-low arousal music on thermal pain intensity. Pain intensity was the lowest when listening to pleasant compared with unpleasant music (*P* < 0.05) and showed a negative correlation with valence but no correlation with arousal, suggesting that the hypoalgesic effects were driven by valence, not arousal. Likewise, Silvestrini,^80^ described previously, found that, compared with silence, pleasant music increased pain intensity and sensory and affective pain thresholds (*P* < 0.005), whereas unpleasant music did not.

##### 3.7.1.2. Perceived emotions

Two studies (one not sufficiently powered^96^) compared the pain-reducing effects of happy vs sad music. In Finlay,^33^ described previously, only relaxing music (but not sad or happy music) decreased pain intensity more than no music (*P* = 0.042). There were no significant differences between the 3 music conditions. However, all music conditions improved pain tolerance, with no statistically significant differences in comparative effectiveness (*P*'s < 0.004).

##### 3.7.1.3. Music genre

Four studies, with 3 not sufficiently powered,^29,84,92^ examined music genre as a variable. Çift,^20^ previously described, compared the effects of Turkish art music vs Western art music vs patient-preferred music in individuals receiving shock wave lithotripsy. There were no significant differences in pain intensity between the music conditions (*P*'s < 0.05). However, the study design did not permit disentangling the effects of preference vs genre.

##### 3.7.1.4. Tempo

Yi^93^ (N = 63 healthy adults) manipulated biological rhythm entrainment by making the tempo of a preferred music excerpt faster, slower, or the same as the participants' spontaneous production rates, which is proposed to reflect the action of an endogenous neural oscillator. Thermal pain ratings were significantly reduced by music presented at the individual's spontaneous production rates compared with tempos 15% faster and 15% slower. The faster and slower tempos were not significantly different from each other.

#### 3.7.2. Mediation studies

##### 3.7.2.1. Partial mediation (*a* path)

###### 3.7.2.1.1. Emotional valence

In Becker,^12^ previously discussed, participants reported greater emotional valence when listening to self-selected (*P* < 0.001) but not researcher-selected music vs a podcast.

###### 3.7.2.1.2. Arousal

Becker^12^ did not find significant differences between the conditions for arousal.

##### 3.7.2.2. Partial mediation (*a* path and *b* path)

###### 3.7.2.2.1. Emotional valence

Two studies tested the *a* path (music→valence) and the *b* path (valence→pain). In Garza-Villarreal,^36^ previously described, increased ratings of pleasantness were associated with lower pain intensity (*P* = 0.006), but there was no significant correlation between valence and pain unpleasantness.

Roy^74^ (N = 18 healthy adults) compared the effects of listening to pleasant music vs unpleasant music vs a silent control condition on thermal stimulation pain. Participants rated pain intensity and unpleasantness as well as emotional valence and arousal for each condition. Compared with the silence condition, only pleasant music reduced pain intensity and pain unpleasantness. Within the pleasant music conditions, pain decreased significantly with increases in self-reports of music pleasantness.

###### 3.7.2.2.2. Arousal

Garza-Villarreal^36^ also examined the role of arousal and found that 2 Mozart pieces produced the lowest arousal ratings. Low arousal levels were, in turn, significantly correlated with low pain intensity (*P* < 0.001) and pain unpleasantness (*P* < 0.005). In addition, Roy^74^ identified a significant within-subject *b* path where changes in arousal were correlated with changes in pain ratings (*P* < 0.05).

##### 3.7.2.3. Moderated mediation

Bradt^15^ examined whether self-reported music reward moderated the mediating effect of pain-related self-efficacy in the music therapy trial described earlier. Music reward was not a significant moderator.

#### 3.7.3. Moderation

##### 3.7.3.1. Music preference

Two studies (one insufficiently powered^70^) examined music preference (liked vs disliked music) as a potential moderator. Lad^53^ (N = 34 healthy adults) examined whether music preference moderated the effects of an auditory only vs tactile only vs auditory-tactile stimulation condition on pain intensity during force pain induction. The mean force at moderate pain increased more while participants listened to their liked than their disliked song (*P* < 0.001). However, this effect was only present in the auditory-tactile condition.

#### 3.7.4. Prediction

##### 3.7.4.1. Arousal

Linnemann,^56^ as described previously, found that arousal did not predict changes in pain intensity (*P* = 0.223).

##### 3.7.4.2. Music preference and enjoyment

Four studies provide evidence that music preference influences analgesic response. Basiński^10^ (N = 76 healthy adults) tested whether preferred music had a stronger effect than less-preferred music on pain intensity, threshold, and controllability. Participants listened to unfamiliar music excerpts or white noise control during a cold pressor test, rating how much they liked the music after each trial. Greater music preference was associated with lower average (*P* = 0.002) and maximal (*P* = 0.001) pain intensity differences between the music and control conditions. Pain controllability and pain threshold were not significantly impacted by music preference.

Similarly, Werner,^90^ discussed previously, found that greater music preference was associated with lower intensity of experimentally induced pain (*P* < 0.001).

Findings related to the importance of music enjoyment in the pain-reducing effects of music were mixed. In Howlin,^45^ discussed previously, greater enjoyment of the music was associated with lower pain intensity and unpleasantness and greater pain tolerance (*P'*s < 0.001). By contrast, Dunbar^28^ (N = 41 healthy adults) found that enjoyment was not associated with changes in pain tolerance (*P* = 0.941). In their study, participants were asked to nod their head or sit still while listening to a dance music compilation. Before and after music listening, pain tolerance was measured by the duration of a chair-sit exercise, and participants rated how much they enjoyed the music.

##### 3.7.4.3. Music familiarity

Two studies reported that participants' familiarity with music selected by the experimenter did not predict music's effect on experimentally induced pain.^28,90^

##### 3.7.4.4. Perceived musicality, musical training, and musical proficiency

Howlin^45^ and Dunbar^28^ found that perceived musicality was not a predictor of the effects of music listening (*P* = NS and *P* = 0.168, respectively). However, LiKamWa^55^ found that greater perceived proficiency (*P* = 0.015) and comfort with singing (*P* < 0.01) were associated with larger pain-reducing effects of singing. LiKamWa^55^ found that musical training was associated with increased time to reach pain tolerance (*P* = 0.05), whereas Howlin^45^ did not find a training effect.

##### 3.7.4.5. Frequency of music behaviors

Dunbar^28^ found that neither frequency of music listening (*P* = 0.454) nor active engagement with music (*P* = 0.178) was associated with changes in pain tolerance in an experimental pain study. Linnemann,^56^ in their study with individuals with fibromyalgia, found that greater daily music listening was associated with greater perceived control over their pain (*P* = 0.041), but not with changes in pain intensity. Mitchell^65^ found that the more participants listened at home to a piece of music they brought in for use during a pain experiment, the more effective the music was in reducing anxiety (*P* < 0.05), the target mediator in their study.

##### 3.7.4.6. Music-evoked feelings

Mitchell^65^ also asked participants to report the feelings evoked by their self-selected piece of music. Music perceived as uplifting, thoughtful, and cheerful was associated with the greatest level of perceived control (*P* < 0.05). Music judged as cheerful was also associated with lower pain intensity than noncheerful music (*P* < 0.05). Linnemann^56^ reported that music listening increased perceived control over pain (*P* = 0.041), especially when the music was perceived as happy. Perceived emotions (happy-sad) were not associated with changes in pain intensity (*P* = 0.743).

### 3.8. Demographic candidate variables

Ten studies assessed potential moderation or prediction effects of demographic variables. However, 4 were underpowered.^3,4,62,92^

#### 3.8.1. Moderation studies

Two studies provided evidence that sex assigned at birth may moderate the effectiveness of MBIs for pain. Kenntner-Mabiala,^51^ as discussed previously, found a significant sex × tempo interaction for arousal (*P* = 0.034), pain intensity (*P* = 0.05), and pain unpleasantness (*P* < 0.05) and that arousal was significantly affected in both sexes, but the effect was stronger for female individuals. Pain intensity and unpleasantness were significantly reduced by slower tempos, but only in female individuals. These findings suggest that sex moderated the impact of music tempo on pain-related outcomes, with female individuals being more responsive to tempo variations.

Similarly, Mitchell^64^ (N = 54 healthy adults) compared the effects of listening to participant-selected music vs relaxing music vs white noise and found that preferred music reduced pain intensity (*P* < 0.01) and the affective component of pain (*P* < 0.05) more effectively in female than in male individuals. No sex differences were observed for the sensory dimension of pain, pain tolerance, or perceived control. By contrast to the aforementioned findings for pain intensity, Shim^79^ (n = 74 female and n = 74 male urology patients) found no difference between male and female participants in the effects of listening to preferred music vs silence on pain intensity.

Pando-Naude^69^ reported no significant moderation effect of age on music-induced resting-state functional connectivity alterations.

#### 3.8.2. Prediction

Two studies tested age as a predictor.^45,46^ Neither found evidence that age predicted the effects of music on pain-related variables.

## 4. Discussion

This scoping review summarized the evidence from 57 studies examining the mechanisms, moderators, and/or predictors of MBIs for pain. Several studies experimentally manipulated candidate variables (eg, emotional valence) through carefully controlled conditions that isolated the candidate mechanism variable of interest. However, most studies tested only the *a* path (music→mediator) without evaluating the *b* path (mediator→pain) or the full indirect (*a* × *b*) pathway. Without these additional tests, it remains unclear whether music's effect on these mediators reflects pain-related mechanisms or independent processes. Even so, several promising candidate mechanisms emerged across studies, including positive emotional valence, cognitive agency, and sensorimotor synchronization. Neuroimaging findings point to the impact of music on early stages of pain processing as well as on higher-order cognitive and affective interpretation of pain. Finally, preliminary evidence suggests the possibility that music may restore default mode network connectivity involved in self-referential thoughts in chronic pain. The findings have important implications for informing future research on the mechanisms of MBIs for pain management.

### 4.1. Positive valence and its relationship with preference, relaxation, and distraction

#### 4.1.1. Valence

Positive emotional valence (pleasantness) consistently emerged as a potential determinant of music's hypoalgesic effects.^1,36,73,74,80^ Furthermore, music enjoyment was often a stronger predictor of hypoalgesia than specific musical features or specific perceived emotions elicited by music.^20,45^ For example, whether music was perceived as happy or sad was not associated with the effects of MBIs on pain intensity,^33^ suggesting that affective tone, rather than categorical emotion, may be the critical factor. Partial mediation findings also suggest that positive affect may link music listening to pain reduction,^80,89^ but full mediation testing remains rare.

Importantly, music may not be unique in its ability to evoke positive affective states that attenuate pain. For instance, Garza-Villareal^36^ found that pleasurable nature sounds and music produced equivalent hypoalgesic effects when both were rated as equally pleasant. Thus, musical pleasure may represent a particularly engaging but not exclusive route to inducing positive affect for hypoalgesia. Future research should clarify which individuals are most responsive to music-evoked pleasure and whether interventions can enhance this responsiveness among less reactive listeners.

#### 4.1.2. Preference

Music preference is closely related to, but conceptually distinct from, emotional valence.^10,20,33,53,65,90^ Preference relates to the implicit value a listener assigns to a piece based on factors such as context, mood state, and biographical meaning, whereas emotional valence refers to the felt emotional quality of the listener's experience. Preferred music is typically pleasant, but it also engages self-relevant processes tied to identity, autobiographical memory, and personal meaning. These associations may enhance attentional engagement or self-referential processing beyond mere enjoyment. Future research should test whether this self-relevance provides incremental hypoalgesic benefits beyond positive affect alone, potentially revealing a mechanism through which personalization may strengthen therapeutic impact.

#### 4.1.3. Relaxation

**Figure 3.**
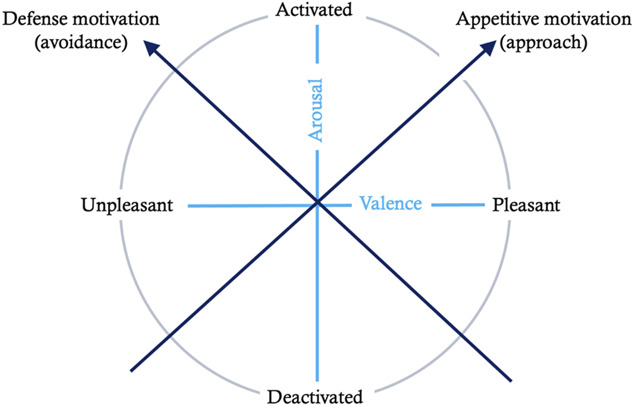
Two-dimensional affective space of emotional experience, adapted from Russell.^75^ The horizontal axis represents valence (pleasantness-unpleasantness), and the vertical axis represents arousal (degree of physiological activation). Overlaid diagonal axes (rotated 45°) reflect appetitive (approach) and aversive (avoidance) motivational dimensions, adapted from Bradley,^14^ which capture the extent to which emotional stimuli engage approach and defensive motivational systems.

Several studies found that music reduced physiological arousal^36,51^ and anxiety,^20,33,50,65^ consistent with a relaxation-mediated pathway. Within Russell's circumplex model of affect^75^ (Fig. 3), relaxation represents 1 quadrant of positive affect: low arousal + high valence. Future research should test whether low-arousal pleasant music produces stronger hypoalgesia than high-arousal pleasant music. From a motivational perspective, music may reduce pain by dampening avoidance systems (through relaxation), activating approach systems (through reward and pleasure), or both.^14,88^ Disentangling these processes as well as their underlying neural substrates is key for understanding how music modifies emotional-motivational systems to modulate pain.

#### 4.1.4. Distraction

Although distraction is often cited as a potential mechanism, current evidence suggests that attentional capture alone is insufficient for hypoalgesia. Unpleasant music or neutral sounds, while engaging attention, typically fail to reduce pain. Moreover, some studies found that music had beneficial effects on pain even when it was presented before, rather than during, pain induction.^36,69,73,74^ These results suggest that the analgesic effects were not attributable to distraction. Thus, music does not appear to reduce pain merely through the automatic capture of attention by an auditory stimulus. However, it remains possible that listeners sustain attention toward the music in a more deliberate and controlled manner. This interpretation aligns with neurobiological models of emotion emphasizing dopaminergic activation of the approach system (Fig. 3) (eg, Ref. 76) and with theories of flow and absorption.^23^ Future research should distinguish automatic attention capture driven by the mere presence of an auditory stimulus from a more sustained state of absorption associated with music listening.

### 4.2. Cognitive agency

Cognitive agency, operationalized as perceived control over music selection in laboratory studies, was consistently associated with improved pain outcomes.^45,46^ These experimental findings parallel clinical evidence that music therapy's benefits on pain intensity and interference are mediated by pain-related self-efficacy.^15^ Together, these results support psychological empowerment, through choice, control, or self-efficacy, as a plausible mechanism of music-induced hypoalgesia. The Cognitive Vitality Model^44^ posits that agency-related mechanisms can be harnessed in MBIs to enhance cognitive engagement and motivation. However, disentangling agency from related constructs remains a challenge. For example, when participants select music from limited experimenter-provided options, observed benefits may partly reflect enjoyment or preference rather than agency per se. Future studies should independently manipulate these constructs to determine their distinct and interactive contributions.

### 4.3. Sensorimotor synchronization

Sensorimotor synchronization—the coordination of movement with rhythmic elements of music—appears to amplify music's hypoalgesic effects. Tapping/drumming along with music produces greater reductions in pain intensity than passive listening or tapping/drumming without music,^68,90^ suggesting synergistic motor-affective mechanisms. Motor engagement itself is known to have hypoalgesic effects^8,72^ and stimulation of the motor cortex is a well-established treatment for chronic pain.^7^ Even passive listening activates motor cortical regions,^40^ consistent with automatic entrainment.^26^ Thus, sensorimotor synchronization may contribute to music-induced analgesia even without tapping/drumming, with tapping/drumming amplifying these effects. Supporting this possibility, neuroimaging findings show that music reduces noxious-evoked activity in motor areas such as the precentral gyrus.^57^ Rhythmic engagement of the motor system may reduce responsiveness to nociceptive input while also enhancing pleasure and agency. These findings point to a broader, embodied mechanism through which rhythmic movement and affective engagement may jointly contribute to music-induced analgesia.

### 4.4. Neural findings

Neural data acquisition studies included in our review varied widely in functional brain measures (EEG, fNIRS, fMRI) and pain models (experimentally induced and chronic pain), complicating identification of specific mechanisms. Findings from experimental pain models are generally more interpretable because the physiology of evoked pain is better characterized. Evidence of reduced pain-evoked responses during music listening in somatomotor regions that receive direct nociceptive input from the spinal cord and thalamus suggests that music may influence early stages of pain processing.^27,95^ These findings align with earlier results obtained using spinal imaging^25^ and nociceptive flexion reflex paradigms.^73,80^ In addition, changes in prefrontal activity during music exposure suggest that music may also modulate higher-order cognitive and affective appraisals of pain.^27,69,78^

In chronic pain populations, 2 studies^37,69^ suggest that self-selected music may help restore normal functional connectivity among regions of the Default Mode Network (DMN) (eg, precuneus, medial prefrontal cortex) that are typically active during self-referential thought. In chronic pain, DMN connectivity is often disrupted, possibly because persistent pain fosters intrusive, self-focused rumination. Following music exposure, DMN connectivity in individuals with fibromyalgia more closely resembled that of healthy controls,^69^ potentially reflecting a shift in neural processing resources from self-referential rumination toward extrinsic processing of the music, thereby reducing the subjective experience of pain.

Although current research has identified music-evoked changes in neural activity during pain, it has not yet delineated how music-induced psychological states (eg, pleasure, relaxation) influence this neural activity. Typically, these hypothesized mediators are measured through self-report or experimental manipulation, and their neural correlates are inferred indirectly from changes in pain-related neural activity. For example, if music is hypothesized to reduce pain through reward circuitry, activation of reward-related regions should be explicitly examined to verify the hypothesized mechanisms. Future studies should therefore first isolate the neural effects of music within brain regions linked to promising psychological mediators identified in this review (eg, positive valence and the reward system,^94^ self-referential thought and the DMN^69^) and then examine how this neural activation influences pain-related neural responses, with 1 avenue consisting of whole-brain mediation techniques.^6^

### 4.5. Limitations of current evidence and recommendations for future research

Across studies, several recurring methodological limitations constrained mechanistic inferences. Many studies were underpowered, increasing risks of false positives and false negatives; such findings were excluded from interpretation in this review. Another pervasive issue was the mismatch between stated aims and analytical approaches. Mediation was often inferred from simple correlations or *a* path analyses, while moderation was typically tested through subgroup comparisons rather than by formal testing of interaction terms. Confusion between mediation, moderation, and prediction was also common. Inconsistent terminology (eg, using *valence* to indicate emotional valence or perceived emotions), lack of definitions or operationalizations (eg, “relaxing music” without specification of the type of music used), and variability in music types and control conditions further obscured mechanistic conclusions.

Future research should employ designs that distinguish between competing mechanistic models and directly test hypothesized pathways (eg, multiple mediator models). Incorporating physiological and neural measures within these frameworks will help bridge psychological and biological levels of analysis. When feasible, causal manipulations (eg, pharmacological manipulation with agonists/antagonists, brain stimulation) should be incorporated to test proposed mediators.

Studies should extend beyond immediate pain responses to examine sustained effects of MBIs, clarifying which mechanisms contribute to enduring clinical benefits. Most studies included in this review were conducted with healthy volunteers using experimentally induced pain. Although this approach is mechanistically informative, pain processing differs in individuals with chronic pain (eg, due to central sensitization).^41^ Future research should examine whether music influences pain processing differently in chronic pain population.

Finally, most studies focused on music listening. Four studies used active music-making, only one of which included an MBI delivered by a music therapist. Additional mechanism studies of music therapy and active music engagement are much needed.

## 5. Conclusion

MBIs show clear potential for pain relief, yet mechanistic understanding remains constrained by inconsistent conceptualization and design. This scoping review identified convergent evidence for several candidate mechanisms—most notably positive valence, cognitive agency, and sensorimotor synchronization—while highlighting the need for rigorous, theory-driven studies to test their causal roles. By clarifying distinctions between mediation, moderation, and prediction, and by promoting methodologically sound approaches, this review provides a conceptual foundation for future mechanistic research in music and pain. A clearer understanding of these processes will support more targeted and effective applications of MBIs in pain management.

## Disclosures

One study included in this review was by the lead author (J.B.) and three studies by the senior author (M.R.). The remaining authors have no conflict of interest to declare.

## Supplemental digital content

Supplemental digital content associated with this article can be found online at http://links.lww.com/PR9/A422.
