# Supplementary material for: A scoping review of music-based pain treatment mechanism research
Source: Pain Rep. 2026 Jul 22;11(4):e1467. doi: 10.1097/PR9.0000000000001467 (PMC13395504; doi:10.1097/PR9.0000000000001467)
Supplement: Supplementary file 1 [file painreports-11-e1467-s001.pdf]

Table of Contents

Table of Contents 1
Search Strategies 3
- Medline 3
- Embase 4
- PsycInfo 4
- Pubmed 5
- SCOPUS 5

Covidence Data Extraction Template 7
Physiological candidate variables 13
- Mediation studies 13

Psychological candidate variables 20
- Experimental manipulation studies 20
- Mediation studies 22
- Moderated mediation studies 36
- Moderation studies 37
- Prediction studies 39

Brain imaging candidate variables 40
- Mediation studies 40

Social candidate variables 44

Mediation studies 44
Moderation studies 44
Music-specific candidate variables 45
Experimental manipulation studies 45
Mediation studies 53
Moderation studies 56
Prediction studies 56
Demographic candidate variables 59
Moderation studies 59
Prediction studies 60

Search Strategies

Medline

Search #1

1	music/
2	music therapy/
3	(music$ or pitch or melod$ or tempo or timbre or tonalit$).ab,ti.
4	(singing or song$).ab,ti.
5	or/1-4
6	mechanis$.ab,ti.
7	mediat$.ab,ti.
8	(moderator$ or moderates or moderating).ab,ti.
9	(regression$ or predict$).ab,ti.
10	modulat$.ab,ti.
11	(control or random$ or experiment$ or condition$ or compared or comparison or cross-over).ab.
12	(pain or nocicept$ or noxious or analges$ or hypoalges$).ab,ti.
13	coping mechanism$.ab,ti.
14	protocol.ti.
15	or/6-10
16	5 and 11 and 12 and 15
17	16 not 13
18	17 not 14
19	limit 18 to (english language and humans)

Search #2

1.	music/
2.	music therapy/
3.	(music$ or pitch or melod$ or tempo or timbre or tonalit$).ab,ti.
4.	(singing or song$).ab,ti.
5.	or/1-4
6.	(pain or nocicept$ or noxious or analges$ or hypoalges$).ab,ti.
7.	(control or random$ or experiment$ or condition$ or compared or comparison or cross-over).ab.
8.	5 and 6 and 7
9.	(Neuro-imaging or neuroimaging or magnetic resonance imaging or MRI or positron emission tomography or PET or electroencephalogram or EEG or neurophysiolog$ or connectivity)).ab,ti.

10.	(reward or limbic or mesolimbic or dopamin$ or valence or pleasur$ or pleasant$ or unpleasant$).ab,ti.
11.	(((preferred or preference or chill$) adj3 music) or peak experience or arousal).ab.
12.	9 or 10 or 11
13.	5 and 8 and 12
14.	limit 13 to (english language and humans)
15.	protocol.ti.
16.	14 not 15

Embase

Search #1

#4 #3 AND ('clinical trial'/de OR 'controlled clinical trial'/de OR 'controlled study'/de OR 'experimental study'/de OR 'human'/de OR 'human experiment'/de OR 'major clinical study'/de OR 'prospective study'/de OR 'randomized controlled trial'/de) AND ('article'/it OR 'review'/it) AND [english]/lim

#3 #1 NOT #2

#2 coping:ab,ti AND mechamism*:ab,ti OR protocol:ab,ti

#1 (music*:ab,ti OR pitch:ab,ti OR melod*:ab,ti OR tempo:ab,ti OR timbre:ab,ti OR tonalit*:ab,ti OR singing:ab,ti OR song*:ab,ti) AND (mechanis*:ab,ti OR mediat*:ab,ti OR moderator*:ab,ti OR moderates:ab,ti OR moderating:ab,ti OR regression*:ab,ti OR predict*:ab,ti OR modulat*:ab,ti) AND (control:ab,ti OR random*:ab,ti OR experiment*:ab,ti OR condition*:ab,ti OR compared:ab,ti OR comparison:ab,ti OR 'cross over':ab,ti) AND (pain:ab,ti OR nocicept*:ab,ti OR noxious:ab,ti OR analges*:ab,ti OR hypoalges*:ab,ti)

Search #2

#5 #3 AND #4

#4 (neuro-imaging:ab,ti OR neurostimulating:ab,ti OR ‘magnetic resonance imaging’:ab,ti OR mri:ab,ti OR ‘positron emission tomography’:ab,ti OR electroencephalogram:ab,ti OR eeg:ab,ti OR neurophysiolog*:ab,ti OR connectivity:ab,ti OR reward:ab,ti OR limbic:ab,ti OR mesolimbic:ab,ti OR dopamin*:ab,ti OR valence:ab,ti OR pleasur*:ab,ti OR pleasant*:ab,ti OR unpleasant*:ab,ti OR ((preferred OR preference OR chill*) W/3 music):ab,ti OR ‘peak experience’:ab,ti OR arousal:ab,ti) AND (control:ab,ti OR random*:ab,ti OR experiment*:ab,ti OR condition*:ab,ti OR compared:ab,ti OR comparison:ab,ti OR 'cross over':ab,ti)

#3 #1 NOT #2

#2 coping:ab,ti AND mechamism*:ab,ti OR protocol:ab,ti

#1 (music*:ab,ti OR pitch:ab,ti OR melod*:ab,ti OR tempo:ab,ti OR timbre:ab,ti OR tonalit*:ab,ti OR singing:ab,ti OR song*:ab,ti) AND (pain:ab,ti OR nocicept*:ab,ti OR noxious:ab,ti OR analges*:ab,ti OR hypoalges*:ab,ti)

PsycInfo

Search #1

(TI (mechanis* or mediat* or moderator* or moderates or moderating or regression* or predict* or modulat*)) OR AB (mechanis* or mediat* or moderator* or moderates or moderating or regression* or predict* or modulat*)) AND (TI (control or random* or experiment* or condition* or compared or comparison or cross-over) OR AB (control or random* or experiment* or condition* or compared or comparison or cross-over)) AND (TI (pain or nocicept* or noxious or analges* or hypoalges*) OR AB (pain or nocicept* or noxious or analges* or hypoalges*)) AND ((MA (music)) OR (MA (music therapy)) OR (TI (music* or pitch or melod* or tempo or timbre or tonalit*) OR AB (music* or pitch or melod* or tempo or timbre or tonalit*)) OR (TI (singing or song*) OR AB (singing or song*))) not ((TI (coping mechanism*) OR AB (coping mechanism*)) OR (TI (protocol)))

Search #2
((MA (music)) OR (MA (music therapy)) OR (AB (music$ or pitch or melod$ or tempo or timbre or tonalit$) OR TI (music$ or pitch or melod$ or tempo or timbre or tonalit$)) OR (AB ((singing or song*)) OR TI ((singing or song*)))) AND (TI (pain or nocicept* or noxious or analges* or hypoalges*) OR AB (pain or nocicept* or noxious or analges* or hypoalges*)) AND (TI (control or random* or experiment* or condition* or compared or comparison or cross-over) OR AB (control or random* or experiment* or condition* or compared or comparison or cross-over)) AND ((TI (Neuro-imaging or neuroimaging or magnetic resonance imaging or MRI or positron emission tomography or PET or electroencephalogram or EEG or neurophysiolog* or connectivity)) OR AB (Neuro-imaging or neuroimaging or magnetic resonance imaging or MRI or positron emission tomography or PET or electroencephalogram or EEG or neurophysiolog* or connectivity))) OR (TI (reward or limbic or mesolimbic or dopamin* or valence or pleasur* or pleasant* or unpleasant*) OR AB (reward or limbic or mesolimbic or dopamin* or valence or pleasur* or pleasant* or unpleasant*)) OR (AB (((preferred or preference or chill$) N3 music) or peak experience or arousal)))

Pubmed
((((((music[MeSH Terms]) OR (music therapy[MeSH Subheading])) OR (music*[Title/Abstract] OR pitch[Title/Abstract] OR melod*[Title/Abstract] OR tempo[Title/Abstract] OR timbre[Title/Abstract] OR tonalit*[Title/Abstract] OR singing[Title/Abstract] OR song*[Title/Abstract])) AND (mechanis*[Title/Abstract] OR mediat*[Title/Abstract] OR moderator*[Title/Abstract] OR moderates[Title/Abstract] OR moderating[Title/Abstract] OR regression*[Title/Abstract] OR predict*[Title/Abstract] OR modulat*[Title/Abstract])) AND (control[Title/Abstract] OR random*[Title/Abstract] OR experiment*[Title/Abstract] OR condition*[Title/Abstract] OR compared[Title/Abstract] OR comparison[Title/Abstract] OR cross-over[Title/Abstract])) AND (pain[Title/Abstract] OR nocicept*[Title/Abstract] OR noxious[Title/Abstract] OR analges*[Title/Abstract] OR hypoalges*[Title/Abstract])) NOT (coping mechanism*[Title/Abstract]) OR protocol[Title/Abstract]) Filters: Humans, English

SCOPUS
Search #1
TITLE-ABS ((music* OR pitch OR melod* OR tempo OR timbre OR tonalit* OR singing OR song*) AND (mechanis* OR mediat* OR moderator* OR moderates OR moderating OR regression* OR predict* OR modulat*) AND (control OR random* OR experiment* OR condition* OR compared OR comparison OR cross-over) AND (pain OR nocicept* OR noxious OR analges* OR hypoalges*) AND NOT (coping AND mechanism* OR protocol)) AND (LIMIT-TO (DOCTYPE , "ar") OR LIMIT-TO (DOCTYPE , "re")) AND (LIMIT-TO (EXACTKEYWORD , "Humans")) AND (LIMIT-TO (LANGUAGE , "English"))

Search #2

TITLE-ABS ((music* OR pitch OR melod* OR tempo OR timbre OR tonalit* OR singing OR song*) AND (neuro-imaging OR neurostimulating OR (magnetic AND resonance AND imaging) OR mri OR (positron AND emission AND tomography) OR pet OR electroencephalogram OR eeg OR neurophysiolog* OR connectivity OR reward OR limbic OR mesolimbic OR dopamin* OR valence OR pleasur* OR pleasant* OR unpleasant* OR ((preferred OR preference OR chill*) W/3 music) OR peak AND experience OR arousal) AND (control OR random* OR experiment* OR condition* OR compared OR comparison OR cross-over) AND (pain OR nocicept* OR noxious OR analges* OR hypoalges*))

Covidence Data Extraction Template

Covidence #	
Study ID	
Title	
Reviewer Name	
**GENERAL INFORMATION**	
Some studies may have made it through to data extraction stage that you believe should actually be excluded. If after your review of this study, you believe this study should be excluded, indicate here	☐ Exclude (do not proceed with data extraction but please offer brief rationale below)<br>☐ Include
If you decided to exclude this study, please offer brief rationale here:	
Country/countries in which the study was conducted (please select all)	☐ USA<br>☐ Canada<br>☐ UK<br>☐ Germany<br>☐ China<br>☐ Not clear/not reported<br>☐ Other (enter)
**STUDY AIMS**	
Did the investigators aim to study mechanisms, moderators, or predictors, as indicated by mention of any one or more of the following words in the Introduction or Method section when referring to the study aims: mechanism, mediator/mediation, moderator/moderation, predictor/prediction?	☐ Yes<br>☐ No
If yes, please enter the specific aim(s) related to these issues, in the words of the authors:	
If yes, please select type(s) of study aim(s) (check all that apply)	☐ Mechanism/mediation<br>☐ Moderation<br>☐ Prediction<br>☐ Other

If the authors did not explicitly state study aim(s) related to mechanism(s), mediator(s), moderator(s), or predictors of pain-related outcome(s), do you believe this study was in fact testing a potential mechanism, mediator, moderator, or predictor?	☐ Yes<br>☐ No<br>☐ N/A
Please explain and state if they were in fact testing a mechanism/mediator, moderator, or predictor.	
**METHODS**	
Pain Model	
Select the pain model used in this study	☐ Experimentally induced pain model<br>☐ Clinical pain model
For studies that use clinical pain model, please specify:	☐ Pain associated with a health condition (e.g. chronic pain, cancer pain)<br>☐ Procedural pain/surgical pain
For studies that use experimentally induced pain, please select the nociceptive stimulus (select all that apply):	☐ Mechanical pressure<br>☐ Heat presented by an aversive stimulation device<br>☐ Cold stimulation presented by an aversive stimulation device<br>☐ Cold pressor pain (placing a hand or forearm in ice water)<br>☐ Electrical stimulation<br>☐ Chemical stimulation
Optional comments for pain model related coding	
Study Design	
Select the study design:	☐ Parallel study design<br>☐ Cross-over/repeated measures
Randomization	☐ Randomized<br>☐ Non-randomized (including quasi-experimental)<br>☐ Other
How many treatment arms (parallel study design) or conditions (cross-over/repeated measures) were included?	
Label each treatment arm/condition, including control condition	
Optional comments for study design related coding	
Participants	
Study population	☐ Healthy volunteers<br>☐ Clinical population
If healthy volunteers:	☐ Healthy volunteers, not otherwise specified<br>☐ Special volunteer group (e.g., musicians)

If special volunteer group, please specify here:	
If clinical population, please provide diagnosis:	
Age range for total sample, if provided	
Age means for total sample, if provided	
Age SD for total sample, if provided	
Percentage of male participants	
Percentage of female participants	
Total N analyzed:	
N analyzed per treatment arm:	
Music condition/intervention	
Enter description(s) of the music condition(s) used	
For clinical trials ONLY, select the interventionist/person who offered the music or music intervention (select all that apply)	☐ Therapist described as a trained or certified music therapist ("Music therapy")<br>☐ Clinical provider, musician, or volunteer who is not a music therapist or whose status as a trained or certified music therapist is not known (“Music Medicine”)<br>☐ Research staff<br>☐ N/A (patient was instructed to listen to their own music on their own, without the presence of music therapist or research staff person)<br>☐ Not a clinical trial<br>☐ Other
For clinical trials ONLY, select the session format:	☐ Individual<br>☐ Group<br>☐ N/A
For clinical studies ONLY, number of music intervention sessions:	
Type of music engagement (select all that apply):	☐ Active music making<br>☐ Receptive (music listening)
For receptive music conditions, specify playback mode (select all that apply):	☐ Recorded music, headset/pillow<br>☐ Recorded music, free field<br>☐ Recorded music, playback mode not specified<br>☐ Live music<br>☐ Other:
Music attributes manipulated by researcher(s) (select all that apply)	☐ Valence<br>☐ Arousal<br>☐ Music preference<br>☐ N/A or none<br>☐ Other:

Music choice (select all that apply)	☐ Selected by researcher or clinical provider<br>☐ Selected by participant from limited set of options provided by researcher or clinical provider (including AI-generated music based on input regarding participant's preferences)<br>☐ Selected by participant from the participant's own music collection (including participant's playist on streaming service or music app).<br>☐ N/A (e.g., music improvisation, live music)<br>☐ Not reported/unclear<br>☐ Other:
For participant selection from limited set, was participant's familiarity with the music assessed?	☐ Yes<br>☐ No<br>☐ Unclear<br>☐ Other:
If you selected unclear, please offer brief explanation	
Optional comments for music-related coding:	
Control condition	
Were one or more no-music control conditions used?	☐ Yes<br>☐ No
If yes, enter brief description(s):	
Optional comments for control condition-related coding:	
**RESULTS**	
Mechanisms/moderators/predictors	
Please list here the MECHANISMS/MEDIATORS that were included in the study and specify for each how they were operationalized or measured (e.g. Beck Depression Inventory; salivary cortisol; or briefly describe the manipulation if it was a variable that was manipulated by the investigator):	
Please list here the MODERATORS that were included in the study and specify for each how they were operationalized or measured :	
Please list here the PREDICTORS that were included in the study and specify for each how they were operationalized or measured:	

Optional comments for mechanism/moderator/predictor related coding:	
Pain outcomes	
What pain outcomes were included? (select all that apply)	☐ Pain intensity<br>☐ Pain threshold (i.e. the point at which a stimulus becomes painful)<br>☐ Pain tolerance (i.e. how long or how much pain a person can withstand)<br>☐ Pain unpleasantness (i.e. the affective dimension of pain)<br>☐ Temporal summation pf pain (i.e. the amount that pain increases when identical stimuli are presented repeatedly in rapid succession)<br>☐ Conditioned pain modulation (i.e. the extent to which one type of pain stimulus is affected by a second painful stimulus)<br>☐ Painful after-sensation (i.e. painful sensations that persist after a stimulus has been removed)<br>☐ Pain quality (i.e. the description of how pain feels such as sharp, dull, burning, or throbbing)<br>☐ Pain interference (i.e. how much pain limits a person's ability to participate in daily activities)<br>☐ Pain medication use<br>☐ Other:
Please describe how the pain outcomes were measured	
Optional comments for pain-related coding	
Select the type(s) of analysis that were used in the study (check all that apply)	☐ Mediation<br>☐ Moderation<br>☐ Predictor<br>☐ Comparison of central tendencies between groups or conditions<br>☐ Other:
Enter description of analysis	
Was this analysis congruent with the study aims?	☐ Yes<br>☐ No<br>☐ Other:
If "no" or "other" please briefly explain here	
Did the investigators present a power analysis to ensure that the PRIMARY AIM was statistically powered?	☐ Yes<br>☐ No<br>☐ N/A (e.g., pilot study)<br>☐ Other:
Optional comments for analysis-related coding:	
**RESULTS**	

If the investigators conducted mediation analysis/es, for each mediation path tested, list the a*b coefficient sizes (if mediation analyses were conducted) with the (1) treatment condition, (2) mediator variable, and (3) outcome variable.	
If they did not conduct mediation analysis/es, describe the results they reported:	
If the investigators conducted moderator test(s), for each moderator test, list the Treatment X Moderator term effect size statistic with the (1) treatment conditions, (2) moderator variable, and (3) outcome variable.	
If they did not conduct moderation test(s), describe the results they reported:	
If the investigators conducted prediction analysis/es, for each prediction analysis conducted, list the association statistic effect size with the (1) treatment condition and (2) outcome variable.	
If they did not conduct predictor analysis test(s), describe the results they reported:	
**SUMMARY OF FINDINGS**	
List the author(s)' conclusions	
Whether or not the study has explicit mechanism, moderation, or prediction aim, did authors discuss implications of their findings with regards to potential mechanisms, moderators, or predictors?	☐ Yes<br>☐ No
If yes, please briefly describe here	
If yes, are those interpretations justified by the data in your opinion?	☐ Yes<br>☐ No<br>☐ Some but not all
Please enter a brief rationale for your assessment	
**STRENGTHS AND WEAKNESSES**	
Summarize study key strengths	
Summarize study key weaknesses	

Physiological candidate variables

Mediation studies

Author (year)	Comparison	Mediator	Outcome	a-path[1]	b-path[1]	a*b[1]	c-path[1]
Becker (2025)[1]	Self-chosen music (SM) vs. researcher-chosen music (RM) vs. podcast (P)	HRV	PT	cvSDNN:<br>SM vs. P: p = 0.096<br>RM vs. P: p = 0.32<br>cvRMSSD<br>SM vs. P: p = 0.37<br>RM vs. P: p = 0.009<br>LF (ms^2)<br>SM vs. P: p = 0.025<br>RM vs. P: p = 0.455<br>HF (ms^2)<br>SM vs. P: p =0.468<br>RM vs. P: p >.001<br>LF/HF-ratio (%)<br>SM vs. P: p = 0.357<br>RM vs. P: p = 0.339	NR	NR	SM vs. P: p = 0.003<br>RM vs. P: p = 0.274<br>SM vs. RM: p = 0.154<br>SM ↑ PT > P, no difference between other conditions
			Pth	cvSDNN:<br>SM vs. P: p = 0.096<br>RM vs. P: p = 0.32<br>cvRMSSD<br>SM vs. P: p = 0.37<br>RM vs. P: p = 0.009<br>LF (ms^2)<br>SM vs. P: p = 0.025<br>RM vs. P: p = 0.455<br>HF (ms^2)<br>SM vs. P: p =0.468<br>RM vs. P: p >.001<br>LF/HF-ratio (%)<br>SM vs. P: p = 0.357<br>RM vs. P: p = 0.339	NR	NR	SM vs. P: p = 0.034<br>RM vs. P: p = 0.018<br>SM vs. RM: p = 0.947<br>SM, RM ↑ PTh > P, no difference between SM and RM

Author (year)	Comparison	Mediator	Outcome	a-path[1]	b-path[1]	a*b[1]	c-path[1]
			PI	cvSDNN:<br>SM vs. P: p = 0.096<br>RM vs. P: p = 0.32<br>cvRMSSD<br>SM vs. P: p = 0.37<br>RM vs. P: p = 0.009<br>LF (ms^2)<br>SM vs. P: p = 0.025<br>RM vs. P: p = 0.455<br>HF (ms^2)<br>SM vs. P: p =0.468<br>RM vs. P: p >.001<br>LF/HF-ratio (%)<br>SM vs. P: p = 0.357<br>RM vs. P: p = 0.339	NR	NR	SM vs. P: p <.001<br>RM vs. P: p = 0.803<br>SM vs. RM: p = 0.002<br>SM ↓ PI > P, RM
			PU	cvSDNN:<br>SM vs. P: p = 0.096<br>RM vs. P: p = 0.32<br>cvRMSSD<br>SM vs. P: p = 0.37<br>RM vs. P: p = 0.009<br>LF (ms^2)<br>SM vs. P: p = 0.025<br>RM vs. P: p = 0.455<br>HF (ms^2)<br>SM vs. P: p =0.468<br>RM vs. P: p >.001<br>LF/HF-ratio (%)<br>SM vs. P: p = 0.357<br>RM vs. P: p = 0.339	NR	NR	SM vs. P: p = 0.007<br>RM vs. P: p = 0.595<br>SM vs. RM: p = 0.075<br>SM ↓ PI > P, no difference between other conditions
Deng (2022)[2]	Music listening (ML) vs. TAU	IL-6	PI	p = 0.001<br>ML ↓ IL-6 > TAU	r = 0.69, p < .01<br>↑ IL-6 → ↑ PI	NT	p < .001<br>ML ↓ PI > TAU
		HMGB-1	PI	p = 0.001<br>ML ↓ HMGB-1 > TAU	r = 0.58 (p < .01)	NT	p < .001<br>ML ↓ PI > TAU
Du (2022)[3]	Music (8 - 150 Hz) vs. TAU	HR	PI	η2 = 0.41 (p = 0.02)<br>Music ↓ HR > TAU	NT	NT	η^2 = 0.43 (p = 0.01)<br>Music ↓ PI > TAU
		HRV	PI	P<.05	NT	NT	η^2 = 0.43 (p = 0.01)<br>Music ↓ PI > TAU

Author (year)	Comparison	Mediator	Outcome	a-path[1]	b-path[1]	a*b[1]	c-path[1]
Garcia (2016)[4]	Relaxing, self-chosen music (RM) vs. motivating, self-chosen music (MM) vs. silence (S)	Pulse rate	PI	NS	NT	NT	NS
			PU	NS	NT	NT	F (2,56) = 3.60, p = .034<br>S vs. RM: d = 0.49 (p = .007)<br>S ↑ PU > RM
		SBP	PI	NS	NT	NT	NS
			PU	NS	NT	NT	F (2,56) = 3.60, p = .034<br>S vs. RM: d = 0.49 (p = .007)<br>S ↑ PU > RM
		DBP	PI	NS	NT	NT	NS
			PU	NS	NT	NT	F (2,56) = 3.60, p = .034<br>S vs. RM: d = 0.49 (p = .007)<br>S ↑ PU > RM
Kavak Akelma (2020)[5]	Favorite music (FM) vs. TAU	HR	PI	p = .001<br>FM ↓ HR > TAU	NT	NT	NS
		SBP	PI	p = .001<br>FM ↓ SBP > TAU	NT	NT	NS
		DBP	PI	p = .001<br>FM ↓ DBP > TAU	NT	NT	NS
		MAP	PI	p = .001	NT	NT	NS
Kenntner-Mabiala (2007)[6]	Fast vs. medium vs. slow tempo	HR	PI	F(2, 50) = 2.5, p = .097	NT	NT	F(2, 32) = 4.8 (p = .016) for female participants only. Slow tempo ↓ PI > medium, fast
		HR	PU	F(2, 50) = 2.5, p = .097	NT	NT	F(2, 32) = 5.1 (p = .02) for female participants only. Slow tempo ↓ PU > medium, fast
		RR	PI	F(2, 46) = 5.6, p = .011<br>Fast tempo: largest ↑	NT	NT	F(2, 32) = 4.8 (p = .016) for female participants only. Slow tempo ↓ PI > medium, fast
		RR	PU	F(2, 46) = 5.6, p = .011<br>Fast tempo: largest ↑	NT	NT	F(2, 32) = 5.1 (p = .02) for female participants only. Slow tempo ↓ PU > medium, fast

Author (year)	Comparison	Mediator	Outcome	a-path[1]	b-path[1]	a*b[1]	c-path[1]
		End-tidal PC02	PI	NS	NT	NT	F(2, 32) = 4.8 (p = .016) for female participants only. Slow tempo ↓ PI > medium, fast
		End-tidal PC02	PU	NS	NT	NT	F(2, 32) = 5.1 (p = .02) for female participants only. Slow tempo ↓ PU > medium, fast
Linnemann (2015)[7]	Music listening (ML) vs. no music	sCort	PI	UC = -0.06 (p = .488)	NT	NT	UC = 1.64 (p = .317)
		sAA	PI	UC = -0.01 (p = .938)	NT	NT	UC = 1.64 (p = .317)
		sCort	Perceived control over pain	UC = -0.06 (p = .488)	NT	NT	UC = 0.30 (p < .001)<br>ML ↑ perceived control
		sAA	Perceived control over pain	UC = -0.01 (p = .938)	NT	NT	UC = 0.30 (p < .001)<br>ML ↑ perceived control
Ortega (2019)[8]	Slow-paced music vs. no music	HR	PI	p = .132	NT	NT	p < .001<br>Music ↓ PI > no music
		SBP	PI	p < .0001<br>Music ↓ SBP > no music	NT	NT	p < .001<br>Music ↓ PI > no music
		DBP	PI	p = .119	NT	NT	p < .001<br>Music ↓ PI > no music
Roy (2012)[9]	Pleasant-stimulating music (PS) vs. pleasant-relaxing music (PR) vs. unpleasant-stimulating music (US) vs. silence (S)	Spinal cord nociception	PI	n^2 = 0.22, p < .001<br>US vs. PS and PR combined: d = 0.76, p = .038<br>PS vs PR: d = 0.20, p = 0.631<br>S vs. musical conditions: *d* = 1.42, p <.001	NT	NT	n^2 = 0.40, p < 0.001<br>US vs. PS: *d* = 1.75, p < 0.001<br>US vs PR: *d = 2.16*, p < 0.001<br>PS vs. PR: *d = .35,* p = .35<br>US ↑ PI > PS, PR<br>S vs. PS, p > .05<br>S vs. PR, p = 0.09
		SCR	PI	n^2 = 0.13, p = 0.20<br>US vs. PS and PR combined: d =0.44, p = .197<br>PS vs. PR: d = 0.38, p = .349<br>S vs. musical conditions: *d* = 0.87, p <.05	NT	NT	n^2 = 0.40, p < 0.001<br>US vs. PS: *d* = 1.75, p < 0.001<br>US vs PR: *d = 2.16*, p < 0.001<br>PS vs. PR: *d = .35,* p = .35<br>US ↑ PI > PS, PR<br>S vs. PS, p > .05<br>S vs. PR, p = 0.09

Author (year)	Comparison	Mediator	Outcome	a-path[1]	b-path[1]	a*b[1]	c-path[1]
Silvestrini (2011)[10]	Pleasant music (PM) vs. unpleasant music (UM) vs. silence vs. auditory attention task (AAT)	PI threshold	NFR	$F(2.29, 41.28) = 1.46$, $P > .20$	NT	NT	$F(2.03, 36.53) = 6.38$, $p < .004$<br>PM vs. S: $p < .005$<br>UM vs. S: no difference<br>AAT vs. S: $p < .005$<br>PM and AAT ↑ PI threshold > UM, S
Tollabzadeh (2023)[11]	Music listening (ML) vs. counseling control	sCort	PI, PU	$p < .001$<br>ML↓ sCort > control	NT	NT	$p < .001$<br>ML↓ PI, PU > control
Van der Valk Bouman (2026)[12]	Silence (S) vs 1-min music listening (ML) vs. 5-min ML vs. 20-min ML	HRV	PT	<u>cvSDNN</u>:<br>1 vs. S: p = 0.014<br>5 vs. S: p = 0.706<br>20 vs. S: p = 0.004<br>1, 20 ↓cvSDNN > S<br><u>cvRMSSD</u>:<br>1 vs. S: p = 0.001<br>5 vs. S: p = 0.05<br>20 vs. S: p = 0.001<br>1, 20 ↓cvRMSSD > S<br><u>LF (ms^2):</u><br>1 vs. S: p <.001<br>5 vs. S: p = 0.03<br>20 vs. S: p <.001<br>1, 5, 20 ↓ LF > S<br><u>HF (ms^2):</u><br>1 vs. S: p = 0.032<br>5 vs. S: p = 0.891<br>20 vs. S: p = 0.009<br>1, 20 ↓ LF > S<br><u>LF/HF-ratio (%):</u><br>1 vs. S: p = 0.174<br>5 vs. S: p = 0.929<br>20 vs. S: p = 0.334<br>No sign differences	NT	NT	ML vs S: p<.001<br>No dif.ferences between music group<br>PT ↑ during all music conditions

Author (year)	Comparison	Mediator	Outcome	a-path[1]	b-path[1]	a*b[1]	c-path[1]
			PTh	cvSDNN:<br>1 vs. S: p = 0.014<br>5 vs. S: p = 0.706<br>20 vs S: p = 0.004<br>1, 20 ↓cvSDNN > S<br>cvRMSSD:<br>1 vs. S: p = 0.001<br>5 vs. S: p = 0.05<br>20 vs S: p = 0.001<br>1, 20 ↓cvRMSSD > S<br>LF (ms^2):<br>1 vs. S: p <.001<br>5 vs. S: p = 0.03<br>20 vs S: p <.001<br>1, 5, 20 ↓ LF > S<br>HF (ms^2):<br>1 vs. S: p = 0.032<br>5 vs. S: p = 0.891<br>20 vs S: p = 0.009<br>1, 20 ↓ LF > S<br>LF/HF-ratio (%):<br>1 vs. S: p = 0.174<br>5 vs. S: p = 0.929<br>20 vs S: p = 0.334<br>No sign differences	NT		1 vs S: p = 0.108<br>5 vs S: p = 0.245<br>20 vs S: p = 0.038<br>20 ↑ PT > S
			PI	cvSDNN:<br>1 vs. S: p = 0.014<br>5 vs. S: p = 0.706<br>20 vs S: p = 0.004<br>1, 20 ↓cvSDNN > S<br>cvRMSSD:<br>1 vs. S: p = 0.001<br>5 vs. S: p = 0.05<br>20 vs S: p = 0.001<br>1, 20 ↓cvRMSSD > S<br>LF (ms^2):<br>1 vs. S: p <.001<br>5 vs. S: p = 0.03<br>20 vs S: p <.001<br>1, 5, 20 ↓ LF > S	NT		1 vs S: p = 0.057<br>5 vs S: p = 0.08<br>20 vs S: p = 0.249<br>No sign differences between music and S

		HF (ms^2):<br>1 vs. S: p = 0.032<br>5 vs. S: p = 0.891<br>20 vs S: p = 0.009<br>1, 20 ↓ LF > S<br>LF/HF-ratio (%):<br>1 vs. S: p = 0.174<br>5 vs. S: p = 0.929<br>20 vs S: p = 0.334<br>No sign differences		
	PU	cvSDNN:<br>1 vs. S: p = 0.014<br>5 vs. S: p = 0.706<br>20 vs S: p = 0.004<br>1, 20 ↓cvSDNN > S<br>cvRMSSD:<br>1 vs. S: p = 0.001<br>5 vs. S: p = 0.05<br>20 vs S: p = 0.001<br>1, 20 ↓cvRMSSD > S<br>LF (ms^2):<br>1 vs. S: p <.001<br>5 vs. S: p = 0.03<br>20 vs S: p <.001<br>1, 5, 20 ↓ LF > S<br>HF (ms^2):<br>1 vs. S: p = 0.032<br>5 vs. S: p = 0.891<br>20 vs S: p = 0.009<br>1, 20 ↓ LF > S<br>LF/HF-ratio (%):<br>1 vs. S: p = 0.174<br>5 vs. S: p = 0.929<br>20 vs S: p = 0.334<br>No sign differences	NT	1 vs S: p = 0.115<br>5 vs S: p = .007<br>20 vs S: p = 0.305<br>5 ↓ PU > S

[1] Effect size, test statistic, and/or p-value (depending on what is reported in the study report)

cvSDNN: coefficient of variation of standard deviation of NN intervals, cvRMSSD: coefficient of root mean square of successive differences, DBP: Diastolic blood pressure, End-tidal PCO2: End-tidal pressure of carbon dioxide, HMGB-1: High Mobility Group Box 1, HR: heart rate, HF: high frequency , HRV: heart rate variability, IL-6: Interleukin-6, LF: low frequency, MAP: Mean arterial pressure, NR: not reported, NT: not tested, PI: pain intensity, PU: pain unpleasantness; RR: respiratory rate, sAA: salivary alpha-amylase, SBP: Systolic blood pressure, sCort: salivary cortisol, SCR: skin conductance response, TAU: treatment as usual

Psychological candidate variables

Experimental manipulation studies

Author/ year	Conditions	Mediator	Outcome	Means, SDs	Effect size/statistic, interpretation
Arican (2020)5[13]	Passive music listening (PM) vs. music-and-attention-to- music (MAM) vs. music-and-attention-to-pain (MAP) vs. silence (S)	Distraction	PT	NR	MAM vs. S: $p = 0.001$<br>MAP vs. S: $p = 0.013$<br>MAM vs PM: $p = 0.067$<br>MAP vs. PM: $p = 0.212$<br>MAM vs. MAP: $p = 0.907$<br>Engagement in task while listening to music ↑ PT
Howlin (2021)[14]	No choice/relaxing music vs. no choice/motivating music vs. perceived choice (2 songs)/relaxing music vs. perceived choice (2 songs)/ stimulating music vs. perceived choice (4 songs)/ relaxing music vs. perceived choice (4 songs)/ stimulating music	Perceived choice/ Cognitive agency	PI	NR	$F(2, 293) = 4.52$, $p = .01$<br>4C ↓ PI > 2C ($p < .01$)<br>4C ↓ PI > NC ($p < .05$)
			PT	NR	$F(2, 293) = 3.184$, $p = .04$<br>4C ↑ PT > 2C ($p < .05$)<br>2C vs. NC: NS
			PU	NR	$F(2, 293) = 1.85$; $p > 0.05$<br>Music choice did not affect PU
Howlin (2022)[15]	No choice/low complexity music vs. no choice/high complexity music vs. perceived choice/low complexity music vs. perceived choice/high complexity music	Perceived choice/Cognitive agency	PI	NR	$ß = -14.00$, 95% CI [-27.23, -.77]<br>Perceived choice: large effect on PI decrease
			PU	NR	$ß = 1.59$, 95% CI [-1.98, 5.15]<br>Perceived choice: no effect on PU
Pan (2025)[16]	Drumming, in-phase synchrony (in-S) vs. drumming, anti-phase synchrony (anti-S) vs. drumming, asynchrony (a-S)	Sensorimotor synchronization	PI	In-S vs. A-s: $p<.001$<br>Anti-S vs A-s: $p<.001$	$F(2,63) = 31.68$, $P < 0.001$, η2 p = 0.50<br>Drumming in-phase or anti-phase synchrony reduced PI the most

			PU	In-S vs. A-s: p = .002<br>Anti-S vs A-s: p = 0.089	F(2,63) = 6.82, P = 0.002, η2 p = 0.18<br>Only in-phase synchrony drumming reduced PU
Werner (2023)[17]	Music active (music with tapping) (MA) vs. music passive (MP) vs. silence active (silence with tapping) (SA) vs. silence passive (SP)	Sensorimotor synchronization	PI	MA vs. MP: -0.28 (SE: 0.08), d = 0.46, p <. 001<br>MA vs. SA: -0.81 (SE 0.12), d = 0.91, p < .001<br>MA vs. SP: -0.93 (0.13), d = 0.93, p < .001<br>MP vs. SA: -0.52 (0.14), d = 0.49, p < .001<br>MP vs. SP: -0.64 (0.15), d = 0.57, p < .001<br>SA vs. SP: -0.2 (0.09), d = 0.17, p = .098	MA vs. SA: t(58) = -3.50, one-sided p < .001.<br>MA vs. SP: t-test, t(58) = -7.17, one-sided p < .001, d = 0.93<br>Music with tapping (i.e. sensorimotor synchronization) ↓ PI most

NR: not reported, PI: pain intensity, PT: pain tolerance, PU: pain unpleasantness

Mediation studies

Author (year)	Comparison	Mediator	Outcome	a-path[1]	b-path[1]	a*b [95% CI]	c-path[1]
Becker (2025)[1]	Self-chosen music (SM) vs. researcher-chosen music (RM) vs. podcast (P)	Anxiety	PT	SM vs. P: p < 0.001<br>RM vs. P: p = 0.028<br>SM vs. RM: p = 0.235	NT	NT	SM vs. P: p = 0.003<br>RM vs. P: p = 0.274<br>SM vs. RM: p = 0.154<br>SM ↑ PT > P, no difference between other conditions
			PTh	SM vs. P: p < 0.001<br>RM vs. P: p = 0.028<br>SM vs. RM: p = 0.235	NT	NT	SM vs. P: p = 0.034<br>RM vs. P: p = 0.018<br>SM vs. RM: p = 0.947<br>SM, RM ↑ PTh > P, no difference between SM and RM
			PI	SM vs. P: p < 0.001<br>RM vs. P: p = 0.028<br>SM vs. RM: p = 0.235	NT	NT	SM vs. P: p <.001<br>RM vs. P: p = 0.803<br>SM vs. RM: p = 0.002<br>SM ↓ PI > P, RM
			PU	SM vs. P: p < 0.001<br>RM vs. P: p = 0.028<br>SM vs. RM: p = 0.235	NT	NT	SM vs. P: p = 0.007<br>RM vs. P: p = 0.595<br>SM vs. RM: p = 0.075<br>SM ↓ PI > P, no difference between other conditions
Bradt (2024)[2]	Music therapy (MT) vs. social attention control (SAC)	Anxiety	Pain interference	NR	NR	NS[2]	
			PI	NR	NR	NS[2]	
			Participant perception of change (PGIC)	NR	NR	NS[2]	
		Mood	Pain interference	NR	NR	NS[2]	
			PI	NR	NR	NS[2]	
			PGIC	NR	NR	NS[2]	

Author (year)	Comparison	Mediator	Outcome	a-path[1]	b-path[1]	a*b [95% CI]	c-path[1]
		Pain-related self-efficacy (SE)	Pain interference	NR	NR	1.16 [0.02 to 2.51]<br>SE = significant mediator for pain interference	
			PI	NS[1]	NR	0.79 [0.01 to 1.82]<br>SE = significant mediator for PI	
			PGIC	NR	NR	−0.30 [0.66 to 0.00]<br>SE ≠ significant mediator for PGIC	
		Emotional support	Pain interference	NR	NR	NS[2]	
			PI	NR	NR	NS[2]	
			PGIC	NR	NR	NS[2]	
Çift 2020[18]	Turkish art music (TAM) vs. Western classical music (WCM) vs. patient-preferred music (PPM) vs. silence with headphones (SH) vs. silence without headphones (S)	Anxiety	PI	S vs. TAM: p < .01<br>S vs. WCM: p < .05<br>S vs. PPM: p < .001<br>SH vs. TAM: p < .05<br>SH vs. WCM: p < .05<br>SH vs. PPM: p < .001<br>TAM vs. PPM: p < .05<br>WCM vs. PPM: p < .01<br>TM, WCM, PPM ↓ anx > S<br>TM and WCM: no difference<br>PPM ↓ anx > TM or WCM	NT	NT	S vs. TAM: p < .01<br>S vs. WCM: p < .05<br>S vs. PPM: p < .001<br>SH vs. TAM: p < .05<br>SH vs. WCM: p < .05<br>SH vs. PPM: p < .001<br>TAM vs. PPM: p < .05<br>WCM vs. PPM: p < .01<br>Music conditions ↓ PI > silence conditions<br>No significant difference between the 3 music conditions

Author (year)	Comparison	Mediator	Outcome	a-path[1]	b-path[1]	a*b [95% CI]	c-path[1]
Colebaugh (2023)[19]	Favorite music (FM) vs. relaxing music app Unwind (RM) vs. white noise (WN)	Pain catastro-phizing (PC)	Forearm Pressure PTh	p < .001<br>FM vs. RM: effect size r = .28, p < .05<br>FM vs. WN: r = .21, p < .05<br>RM vs. WN: r = .15, NS<br>FM ↓ PC > RM or WN	NS	NT	FM vs. RM: effect size r = .43, p < .05<br>FM vs. WN: r = .39, p < .05<br>RM vs. WN: r = .11, NS<br>FM ↑ PTh > RM or WN
			Forearm Pressure PT	p < .001<br>FM vs. RM: effect size r = .28, p < .05<br>FM vs. WN: r = .21, p < .05<br>RM vs. WN: r = .15, NS<br>FM ↓ PC > RM or WN	NS	NT	FM vs. RM: r = .46, p < .05<br>FM vs. WN: r = .40, p < .05<br>RM vs. WN: r = .15, NS<br>FM ↑ PT > RM or WN
			Trapezius Pressure PTh	p < .001<br>FM vs. RM: effect size r = .28, p < .05<br>FM vs. WN: r = .21, p < .05<br>RM vs. WN: r = .15, NS<br>FM ↓ PC > RM or WN	NS[2]	NT	FM vs. RM: r = .14, NS<br>FM vs. WN: r = .22, p < .05<br>RM vs. WN: r = .07, NS<br>FM ↑ PTh > RM or WN
			Trapezius Pressure PT	p < .001<br>FM vs. RM: effect size r = .28, p < .05<br>FM vs. WN: r = .21, p < .05<br>RM vs. WN: r = .15, NS<br>FM ↓ PC > RM or WN	NS[2]	NT	FM vs. RM: r = .28, p < .05<br>FM vs. WN: r = .27, p < .05<br>RM vs. WN: r = .02, NS<br>FM ↑ PT > RM or WN

Heat PTh	$p < .001$<br>FM vs. RM: effect size $r = .28$, $p < .05$<br>FM vs. WN: $r = .21$, $p < .05$<br>RM vs. WN: $r = .15$, NS<br>FM ↓ PC > RM or WN	NS[2]	NT	FM vs. RM: $r = .19$, $p < .05$<br>FM vs. WN: $r = .20$, $p < .05$<br>RM vs. WN: $r = .00$, NS<br>FM ↑ PTh > RM or WN
Offset analgesia	$p < .001$<br>FM vs. RM: effect size $r = .28$, $p < .05$<br>FM vs. WN: $r = .21$, $p < .05$<br>RM vs. WN: $r = .15$, NS<br>FM ↓ PC > RM or WN	NS[2]	NT	FM vs. RM: $r = .24$, $p < .05$<br>FM vs. WN: $r = .26$, $p < .05$<br>RM vs. WN: $r = .00$, NS<br>FM ↑ Offset analgesia > RM or WN
CPM	$p < .001$<br>FM vs. RM: effect size $r = .28$, $p < .05$<br>FM vs. WN: $r = .21$, $p < .05$<br>RM vs. WN: $r = .15$, NS<br>FM ↓ PC > RM or WN	NS[2]	NT	FM vs. RM: $r = .03$, NS<br>FM vs. WN: $r = .09$, NS<br>RM vs. WN: $r = .04$, NS<br>FM vs. RM vs. WN: No significant difference ($p > .05$)
TSP	$p < .001$<br>FM vs. RM: effect size $r = .28$, $p < .05$<br>FM vs. WN: $r = .21$, $p < .05$<br>RM vs. WN: $r = .15$, NS<br>FM ↓ PC > RM or WN	NS[2]	NT	FM vs. RM: $r = .07$, NS<br>FM vs. WN: $r = .13$, NS<br>RM vs. WN: $r = .06$, NS<br>FM vs. RM vs. WN: No significant difference ($p > .05$)

Author (year)	Comparison	Mediator	Outcome	a-path[1]	b-path[1]	a*b [95% CI]	c-path[1]
Ernberg (2020)[20]	Black metal (BM) vs. classical (Cl) vs. no music (NM)	Anxiety	Peak PI	NS[2]	BM: r = .642, p = .002<br>CM: r = .642, p = .002<br>NM: r = .530, p = .016	NT	NS[2]
			Pain quality	NS[2]	NT	NT	NS[2]
			PD	NS[2]	NT	NT	NS[2]
Finlay (2016)[21]	(1) Happy music (HM), (2) sad music (SM), (3) relaxing music (RM), (4) no music (NM)	Anxiety	PI	F(3, 120) = 8.28, p < .001, η_p^2 = .17<br>HM vs. NM: p = .074<br>SM vs. NM: p = 1.00<br>RM vs. NM: p = .001<br>HM vs. SM: p = .320<br>HM vs. RM: p = .285<br>SM vs. RM: p = .002<br>RM ↓ anx > SM, NM	NM: r = .532, NS<br>HM: r = .385, p < .01<br>SM: r = .466, p < .01<br>RM: r = .255, NS		F(3, 120) = 3.09, p =.030, η_p^2 = .07<br>HM vs. NM: p = .096<br>SM vs. NM: p = .1.00<br>RM vs. NM: p = .042<br>HM vs. SM: p = 1.00<br>HM vs. RM: p = 1.00<br>SM vs. RM: p = .745<br>RM ↓ > NM; no difference between other conditions
			PT	F(3, 120) = 8.28, p < .001, η_p^2 = .17<br>HM vs. NM: p = .074<br>SM vs. NM: p = 1.00<br>RM vs. NM: p = .001<br>HM vs. SM: p = .320<br>HM vs. RM: p = .285<br>SM vs. RM: p = .002<br>RM ↓ anx > SM, NM	NM: r = -.367, p < .05<br>HM: r = -.506, p < .01<br>SM: r = -.245, NS<br>RM: r = -.280, NS		F(3, 120) = 8.36, p < .001, η_p^2 = .17<br>HM vs. NM: p = .001<br>SM vs. NM: p = .004<br>RM vs. NM: p = .001<br>HM vs. SM: p = .233<br>HM vs. RM: p = .961<br>SM vs. RM: p = .133<br>all music conditions ↑PT > NM
		Distraction	PI	F(2.56, 102.54) = 9.43, p < .001, η_p^2 = .19<br>HM vs. NM: p = .001<br>SM vs. NM: p = .055<br>RM vs. NM: p = .001<br>HM vs. SM: p = .002	NM: r = -.214, NS<br>HM: r =-.157, NS<br>SM: r = -.276, NS<br>RM: r = -.223, NS		F(3, 120) = 3.09, p =.030, η_p^2 = .07<br>HM vs. NM: p= .096<br>SM vs. NM: p= .1.00<br>RM vs. NM: p= .042<br>HM vs. SM: p= 1.00<br>HM vs. RM: p= 1.00

		HM vs. RM: p = .512<br>SM vs. RM: p = .013<br>HM and RM ↑ distr > SM, S			SM vs. RM: p= .745<br>RM ↓ > NM; no difference between other conditions
	PT	F(2.56, 102.54) = 9.43, p < .001, η_p^2 = .19<br>HM vs. NM: p = .001<br>SM vs. NM: p = .055<br>RM vs. NM: p = .001<br>HM vs. SM: p = .002<br>HM vs. RM: p = .512<br>SM vs. RM: p = .013<br>HM and RM ↑ distr > SM, S	NM: r = .189, NS<br>HM: r = .411, p < .01<br>SM: r = .416, p < .01<br>RM: r = .429, p < .01		F(3, 120) = 8.36, p < .001, η_p^2 =.17<br>HM vs. NM: p = .001<br>SM vs. NM: p = .004<br>RM vs. NM: p = .001<br>HM vs. SM: p = .233<br>HM vs. RM: p = .961<br>SM vs. RM: p = .133<br>all music conditions ↑PT > NM
Perceived control over pain	PI	F(3, 120) = 4.76, p = .004, η_p^2 = .106<br>HM vs. NM: p = .033<br>SM vs. NM: p = 1.00<br>RM vs. NM: p = .049<br>HM vs. SM: p = .024<br>HM vs. RM: p = 1.00<br>SM vs. RM: p = .134<br>HM and RM ↑ control > NM or SM	NM: r = -.339, p < .05<br>HM: r = -.415, p < .01<br>SM: r = -.447, p < .01<br>RM: r = -.344, p < .05		F(3, 120) = 3.09, p = .030, η_p^2 = .07<br>HM vs. NM: p = .096<br>SM vs. NM: p = .1.00<br>RM vs. NM: p = .042<br>HM vs. SM: p = 1.00<br>HM vs. RM: p = 1.00<br>SM vs. RM: p = .745<br>RM ↓ > NM; no difference between other conditions
	PT	F(3, 120) = 4.76, p = .004, η_p^2 = .106<br>HM vs. NM: p = .033<br>SM vs. NM: p = 1.00<br>RM vs. NM: p = .049<br>HM vs. SM: p = .024<br>HM vs. RM: p = 1.00<br>SM vs. RM: p = .134<br>HM and RM ↑ control > NM or SM	NM: r = .328, p < .05<br>HM: r = .618, p < .001<br>SM: r = .577, p < .001<br>RM: r = .480, p < .05		F(3, 120) = 8.36, p < .001, η_p^2 = .17<br>HM vs. NM: p = .001<br>SM vs. NM: p = .004<br>RM vs. NM: p = .001<br>HM vs. SM: p = .233<br>HM vs. RM: p = .961<br>SM vs. RM: p = .133<br>all music conditions ↑PT > NM

Author (year)	Comparison	Mediator	Outcome	a-path[1]	b-path[1]	a*b [95% CI]	c-path[1]
Garcia (2016)[4]	Motivating music (MM) vs. relaxing music (RM) vs. silence (S)	Mood: depressed-elated	PI	NS[2]	NT	NT	NS[1]
			PU	NS[2]	NT	NT	F(2,56) = 3.60, $p = .034$<br>RM ↓ PU > S, d = 0.49
		Mood: tired-energetic	PI	F(2, 56) = 3.37, $p = .04$<br>MM vs. RM: $p = .09$<br>RM vs. S: $p = .48$<br>MM vs. S: $p = .54$	NT	NT	NS[1]
			PU	F(2, 56) = 3.37, $p = .04$<br>MM vs. RM: $p = .09$<br>RM vs. S: $p = .48$<br>MM vs. S: $p = .54$	NT	NT	F(2,56) = 3.60, $p = .034$<br>RM ↓ PU > S, d = 0.49
Goldfine (2025)[22]	Self-selected music (M) vs. silence (S) (noise-canceling headphones)	Post-intervention anxiety	PI	F(1,36) = 6.40, $p = 0.016$, η_p^2=.15<br>M ↓ anxiety > S	r = 0.54, $p <0.001$	-0.6 [-1.22, -0.09]	F (1,37) = 4.69, $p = 0.037$, η_p^2=.11<br>M ↓ PI > S
Johnson (2020)[23]	Music with relaxation suggestions (MR) vs. music alone (M) vs. silence (S)	Anxiety	PU	NR	NS[2]	NT	MR vs. M vs. S: $p = .14$)
			PI	NR	NS[2]	NT	MR vs. M vs. S: $p = .47$)
			PTh	NR	NS[2]	NT	MR vs. M vs. S: $p = .28$)
			PT	NR	NS[2]	NT	F(2,64) = 3.69, $p < .05$<br>M ↑ PT > S ($p < .05$)<br>MR vs. M: NS[1]
		Relaxation	PU	NR	NS[2]	NT	MR vs. M vs. S: $p = .14$
			PI	NR	NS[2]	NT	MR vs. M vs. S: $p = .47$
			PTh	NR	NS[2]	NT	MR vs. M vs. S: $p = .28$
			PT	NR	MR: $r_p = .61$, $p < .05$<br>↑ relax → ↑ PT	NT	F(2,64) = 3.69, $p < .05$<br>M ↑ PT > S ($p < .05$)<br>MR vs. M: NS[1]

Author (year)	Comparison	Mediator	Outcome	a-path[1]	b-path[1]	a*b [95% CI]	c-path[1]
		Mood (positive affect (PA) and negative affect (NA))	PU	NR	PA: MR: r_p = -.60, p < .05<br>↑ PA → ↓ PU<br>NA: MR: r_p = .58, p < .06<br>↑ NA → ↑ PU	NT	MR vs. M vs. S: p = .14
			PI	NR	NS[2]	NT	MR vs. M vs. S: p = .47
			PTh	NR	NS[2]	NT	MR vs. M vs. S: p = .28
			PT	NR	NS[2]	NT	F(2,64) = 3.69, p < .05<br>M ↑ PT > S (p<.05)<br>MR vs. M: NS[1]
		Perceived control over pain	PU	NR	M: r_p = -.63, p < .05<br>MR: r_p = -.69, p < .01	NT	MR vs. M vs. S: p = .14
			PI	NR	NS[2]	NT	MR vs. M vs. S: p = .47
			PTh	NR	NS[2]	NT	MR vs. M vs. S: p = .28
			PT	NR	NS[2]	NT	F(2,64) = 3.69, p < .05<br>M ↑ PT > S (p < .05)<br>MR vs. M: NS[1]
		Distraction	PU	NR	MR: r_p = − .58, p < .05<br>↑ distr → ↓ PU	NT	MR vs. M vs. S: p = .14
			PI	NR	NS[2]	NT	MR vs. M vs. S: p = .47
			PTh	NR	NS[2]	NT	MR vs. M vs. S: p = .28
			PT	NR	MR: r_p = .62, p < .05<br>↑ distr→ ↑ PT	NT	F(2,64) = 3.69, p < .05<br>M ↑ PT > S (p < .05)<br>MR vs. M: NS[1]
		Response expectancy	PU	NR	NS[2]	NT	MR vs. M vs. S: p = .14
			PI	NR	NS[2]	NT	MR vs. M vs. S: p = .47
			PTh	NR	NS[2]	NT	MR vs. M vs. S: p = .28

			PT	NR	NS[2]	NT	F(2, 64) = 3.69, $p < .05$<br>M ↑ PT > S ($p < .05$)<br>MR vs. M: NS[1]
		Absorption	PU	NR	NS[2]	NT	MR vs. M vs. S: $p = .14$
			PI	NR	NS[2]	NT	MR vs. M vs. S: $p = .47$
			PTh	NR	NS[2]	NT	MR vs. M vs. S: $p = .28$
			PT	NR	NS[2]	NT	F(2, 64) = 3.69, $p < .05$<br>M ↑ PT > S ($p < .05$)<br>MR vs. M: NS[1]
		Hypnotizab i-lity	PU	NR	NS[2]	NT	MR vs. M vs. S: $p = .14$
			PI	NR	NS[2]	NT	MR vs. M vs. S: $p = .47$
			PTh	NR	M: $r_p = .73$, $p < .05$<br>↑ distr→ ↑ PTh	NT	MR vs. M vs. S: $p = .28$
			PT	NR	NS[2]	NT	F(2, 64) = 3.69, $p < .05$<br>M ↑ PT > S ($p < .05$)<br>MR vs. M: NS[1]
Kavak Akelma (2020)[5]	Favorite music (FM) vs. silence (S)	Anxiety-state (anx)	PI	$p = .001$[1]<br>FM ↓ anx > S	NT	NT	NS[1]
LiKamWa (2022)[24]	Singing (SG) vs. music listening (ML) vs. silence (S)	Distraction	PI at threshold point	NR (82.5% found SG the most distracting)[1]	NT	NT	NS[1]
			PI at tolerance point	NR (82.5% found SG the most distracting)[1]	NT	NT	$x^2(2) = 5.73$, $p = 0.057$<br>sG vs. S: $p = 0.002$<br>SG vs. ML: $p = .05$<br>ML vs. S: NS[1]<br>SG ↓PI > ML or S
			PTh	NR (82.5% found SG the most distracting)[1]	NT	NT	$x^2 = 10.2$, $p = .0061$<br>SG vs. S: $p = .002$<br>SG vs. ML: $p = .05$<br>ML vs. S: $p = .264$<br>SG ↑ PTh > ML or S

Author (year)	Comparison	Mediator	Outcome	a-path[1]	b-path[1]	a*b [95% CI]	c-path[1]
			PT	NR (82.5% found SG the most distracting)[1]	NT	NT	x^2 = 24.47, p < .0001<br>SG vs. S: p = .0001<br>SG vs. ML: p = .0008<br>ML vs. S: p = .61<br>SG ↑ PT > ML or S
		Positive/Negative affect	PI at threshold point	NS[2]	NT	NT	NS[1]
			PI at tolerance point	NS[2]	NT	NT	$x^2(2)$ = 5.73, p = 0.057<br>sG vs. S: p = 0.002<br>SG vs. ML: p = .05<br>ML vs. S: NS[1]<br>SG ↓PI > ML or S
			PTh	NS[2]	NT	NT	x^2 = 10.2, p = .006<br>SG vs. S: p = .002<br>SG vs. ML: p = .05<br>ML vs. S: p = .26<br>SG ↑ PTh > ML or S
			PT	NS[2]	NT	NT	x^2 = 24.47, p < .0001<br>SG vs. S: p = .0001<br>SG vs. ML: p = .0008<br>ML vs. S: p = .61<br>SG ↑ PT > ML or S ↑ PT
Mitchell (2008)[25]	Preferred music (PM) vs. Art viewing (AV) vs. Silence (S)	Anxiety-state	PT	F(2, 67) = 9.12, p < .001<br>ML vs. S: p < .001<br>ML vs. AV: p < .001<br>PM ↓ anx > S or AV	NT	NT	F(2,77) = 11.42, p < .001<br>PM vs. S: p < .001<br>PM vs. AV: p < .001<br>PM ↑ PT > S or AV
			PI	F(2, 67) = 9.12, p < .001<br>ML vs. S: p < .001<br>ML vs. AV: p < .001<br>PM ↓ anx > S or AV	NT	NT	F(2,76) = 4.43, p < .05<br>PM vs. S: p < .05<br>PM vs. AV: NR<br>PM ↓ PI > S

		Perceived control over pain	PT	F(2, 72) = 10.76, p < .001<br>ML vs. S: p < .001<br>ML vs. AV: p < .001<br>PM ↑ control < S or AV	NT	NT	F(2,77)= 11.42, p <.001<br>PM vs. S: p<.001<br>PM vs. AV: p<.001<br>PM ↑ PT > S or AV
			PI	F(2, 72) = 10.76, p < .001<br>ML vs. S: p < .001<br>ML vs. AV: p < .001<br>PM ↑ control < S or AV	NT	NT	F(2,76) = 4.43, p < .05<br>PM vs. S: p < .05<br>PM vs. AV: NR<br>PM ↓ PI > S
		Distraction	PT	F(2, 75) = 19.30, p < .001<br>ML vs. S: p < .001<br>ML vs. AV: p < .001<br>PM ↑ dist > S or AV	NT	NT	F(2,77) = 11.42, p < .001<br>PM vs. S: p < .001<br>PM vs. AV: p < .001<br>PM ↑ PT > S or AV
			PI	F(2, 75) = 19.30, p < .001<br>ML vs. S: p < .001<br>ML vs. AV: p < .001<br>PM ↑ dist > S or AV	NT	NT	F(2,76) = 4.43, p < .05<br>PM vs. S: p < .05<br>PM vs. AV: NR<br>PM ↓ PI > S
Ortega (2019)[8]	Music (M) vs. silence (S)	Anxiety-state	PT	p = .0001[1]<br>M ↓ anx > S	NT	NT	P = .0004[1]<br>M ↓ PI > S
			PI	p = .0001[1]<br>M ↓ anx > S	NT	NT	NT
Silvestrini (2011)[10]	Pleasant music (PM) vs. unpleasant music (UM) vs. silence (S) vs. auditory attention task (AAT)	Emotions	PI threshold	F(30, 133) = 3.71, p < .001<br>PM ↑ positive emotions > UM, S, AAT<br>UM ↑ negative emotions > PM or S	NT	NT	F(2.03, 36.53) = 6.38, p < .004<br>PM vs. S: p < .005<br>UM vs. S: no difference<br>AAT vs. S: p < .005<br>PM and AAT ↑ PI threshold > UM, S

Author (year)	Comparison	Mediator	Outcome	a-path[1]	b-path[1]	a*b [95% CI]	c-path[1]
			Sensory threshold	F(30, 133) = 3.71, p < .001<br>PM ↑ positive emotions > UM, S, AAT<br>UM ↑ negative emotions > PM or S	NT	NT	F(2.57, 46.33) = 7.40, p < .001<br>PM vs. S: p < .002<br>UM vs. S: no difference<br>AAT vs. S: p < .001<br>PM and AAT ↑ Sensory threshold > UM, S
			Affective threshold	F(30, 133) = 3.71, p < .001<br>PM ↑ positive emotions > UM, S, AAT<br>UM ↑ negative emotions > PM or S	NT	NT	F(1.74, 31.29) = 3.82, p < .02<br>PM vs. S: p < .02<br>UM vs. S: no difference<br>AAT vs. S: p < .04<br>PM and AAT ↑ affective threshold > UM, S
			PT	F(30, 133) = 3.71, p < .001<br>PM ↑ positive emotions > UM, S, AAT<br>UM ↑ negative emotions > PM or S	NT	NT	F(1.86, 33.43) = 4.05, p < .02<br>PM vs. S: p < .02<br>UM vs. S: no difference<br>AAT vs. S: p < .05<br>PM and AAT ↑ PT > UM, S
		Attention	PI threshold	t > 2.22, p < .02<br>PM, UM, AT ↑Att > S<br>AAT vs. PM: p < .05<br>AAT ↑ att > PM<br>PM vs. UM: NS[1]	NT	NT	F(2.03, 36.53) = 6.38, p < .004<br>PM vs. S: p < .005<br>UM vs. S: no difference<br>AAT vs. S: p < .005<br>PM and AAT ↑ PI threshold > UM, S
			Sensory threshold	t > 2.22, p < .02<br>PM, UM, AT ↑ att > S<br>AAT vs. PM: p < .05<br>AAT ↑ att > PM<br>PM vs. UM: NS[1]	NT	NT	F(2.57, 46.33) = 7.40, p < .001<br>PM vs. S: p < .002<br>UM vs. S: no difference<br>AAT vs. S: p < .001<br>PM and AAT ↑ sensory threshold > UM, S

	Affective threshold	t > 2.22, p < .02<br>PM, UM, AT ↑att > S<br>AAT vs. PM: p < .05<br>AAT ↑ att > PM<br>PM vs. UM: NS[1]	NT	NT	F(1.74, 31.29) = 3.82, p < .02<br>PM vs. S: p < .02<br>UM vs. S: no difference<br>AAT vs. S: p < .04<br>PM and AAT ↑ affective threshold > UM, S
	PT	t > 2.22, p < .02<br>PM, UM, AT ↑att > S<br>AAT vs. PM: p < .05<br>AAT ↑ att > PM<br>PM vs. UM: NS[1]	NT	NT	F(1.86, 33.43) = 4.05, p < .02<br>PM vs. S: p < .02<br>UM vs. S: no difference<br>AAT vs. S: p < .05<br>PM and AAT ↑ PT > UM, S
Distraction from pain	PI threshold	t > 3.90, p < .001<br>PM ↑ distr > S or AAT<br>PM vs. UM: no difference	NT	NT	F(2.03, 36.53) = 6.38, p < .004<br>PM vs. S: p < .005<br>UM vs. S: no difference<br>AAT vs. S: p < .005<br>PM and AAT ↑ PI threshold > UM, S
	Sensory threshold	t > 3.90, p < .001<br>PM ↑ distr > S or AAT<br>PM vs. UM: no difference	NT	NT	F(2.57, 46.33) = 7.40, p < .001<br>PM vs. S: p < .002<br>UM vs. S: no difference<br>AAT vs. S: p < .001<br>PM and AAT ↑ sensory threshold > UM, S
	Affective threshold	t > 3.90, p < .001<br>PM ↑ distr > S or AAT<br>PM vs. UM: no difference	NT	NT	F(1.74, 31.29) = 3.82, p < .02<br>PM vs. S: p < .02<br>UM vs. S: no difference<br>AAT vs. S: p < .04<br>PM and AAT ↑ affective threshold > UM, S
	PT	t > 3.90, p < .001<br>PM ↑ distr > S or AAT<br>PM vs. UM: no difference	NT	NT	F(1.86, 33.43) = 4.05, p < .02<br>PM vs. S: p < .02<br>UM vs. S: no difference<br>AAT vs. S: p < .05<br>PM and AAT ↑ PT > UM, S

Author (year)	Comparison	Mediator	Outcome	a-path[1]	b-path[1]	a*b [95% CI]	c-path[1]
Tollabzadeh (2023)[11]	Preferred music (PM) vs. Counseling (C)	Anxiety	Pain (MPQ total score)	p < .001[1]<br>PM ↓ anx > C	NT	NT	p < .001[1]<br>PM ↓ Pain > C
		Stress	Pain (MPQ total score)	p = .397[1]<br>PM ↓ stress > C	NT	NT	p < .001[1]<br>PM ↓ Pain > C
van der Valk Bouman (2026)[12]	Silence (S) vs 1-min music listening (ML) vs. 5-min ML vs. 20-min ML	Anxiety	PT	1 vs. S: p = 0.7<br>5 vs. S: p = 0.392<br>20 vs S: p = 0.088<br>No effect on anxiety	NT	NT	ML vs S: p<.001<br>No differences between music group<br>PT ↑ during all music conditions
			PTh	1 vs. S: p = 0.7<br>5 vs. S: p = 0.392<br>20 vs S: p = 0.088<br>No effect on anxiety	NT	NT	1 vs S: p = 0.108<br>5 vs S: p = 0.245<br>20 vs S: p = 0.038<br>20 ↑ PT > S
			PI	1 vs. S: p = 0.7<br>5 vs. S: p = 0.392<br>20 vs S: p = 0.088<br>No effect on anxiety	NT	NT	1 vs S: p = 0.057<br>5 vs S: p = 0.08<br>20 vs S: p = 0.249<br>No sign differences between music and S
			PU	1 vs. S: p = 0.7<br>5 vs. S: p = 0.392<br>20 vs S: p = 0.088<br>No effect on anxiety	NT	NT	1 vs S: p = 0.115<br>5 vs S: p = .007<br>20 vs S: p = 0.305<br>5 ↓ PU > S
Weinstein (2016)[26]	Singing in small choir (SSC) vs. Singing in large choir (SLC)	Positive affect (PA)	PTh	U = 3965, Z = −3.1, p = .002, r = .21<br>SSC ↑ PA > SLC	NT	NT	F(1, 105) = 1.5, p = .23<br>PTh ↑ in both SSC and SLC
		Negative affect (NA)	PTh	U = 4603.5, Z = −1.9, p = .061<br>No difference between SSC and SLC	NT	NT	F(1, 105) = 1.5, p = .23<br>PTh ↑ in both SSC and SLC

[1] Effect size, test statistic, and/or p-value (depending on what is reported in the study report)
[2] No test statistic or p-value reported

CPM: conditioned pain modulation, MPQ: McGill Pain Questionnaire, NR: not reported; NS: non-significant; NT: not tested; PD: Pain duration, PI: pain intensity; PT: Pain tolerance; PTh: Pain threshold; PU: pain unpleasantness, TSP: temporal summation of pain

Moderated mediation studies

Author/ Year	Comparison	Mediator	Moderator	Outcome	Effect size, p	Interpretation
Bradt 2024[2]	Music therapy (MT) vs. social attention control (SAC)	Pain-related self-efficacy (SE)	Music reward	PI, Pain interference	NS	
			Adult playfulness	PI, Pain interference	NS	
			Treatment expectancy	PI, Pain interference	NS	
			Baseline pain intensity	PI	NS	
				Pain interference	NS	
			Baseline pain interference	PI	ab = 0.12, 95% [CI 0.001 to 0.30]	The difference between the SAC and MT participants in improving self-efficacy from baseline to session 4 was larger for participants with higher baseline pain interference, which was in turn associated with a larger difference in pain intensity
				Pain interference	ab = 0.21, 95% [CI 0.02 to 0.47]	The difference between the SAC and MT participants in improving self-efficacy from baseline to session 4 was larger for participants with higher baseline pain interference, which was in turn associated with a larger difference in pain interference

NS: non-significant, PI: Pain intensity

Moderation studies

Author/ year	Interaction (Treatment conditions x Moderator)	Outcome	Treatment x moderator explanation and statistics (p)
Choi 2018[27]	Music listening (ML) vs. news listening (NL) vs. silence (S) X high anxiety sensitivity (HAS) vs. normal anxiety sensitivity (NAS)	PI	NAS: ML ↓ PI > NL (p = .017) and S (p < .002)[1]<br>HAS: ML vs. NL (p = .408) and S (p = .291)[1]
		PT	NAS: ML ↑ PT > NL (p = .004) and S (p < .001)[1]<br>HAS: ML vs. NL (p = .834) and S (p = .061)[1]
		PU	NAS: ML ↓ PU > NL (p = .030) and S (p < .001)[1]<br>HAS: ML vs. Nl (p = .819) and S (p = .092)[1]
		PI	NPA: ML ↓ PI > S (p < .001) but not NL (p = .115)[1]<br>HPA: ML vs. NL (p = .096) and S (p = .572)[1]
		PT	NPA:ML ↑ PT > NL (p < .001) and S (p < .001)[1]<br>HPA: ML ↑ PT > NL (p = .004) and S (p < .001)[1]
		PU	NPA: ML ↓ PU > S (p < .001) but not NL (p = .180)[1]<br>HPA: ML ↓ PU > S (p = .033) but not NL (p = .226)[1]
Choi 2022[28]	Music listening (ML) vs. news listening (NL) vs. silence (S) X high anxiety sensitivity (HAS) vs. normal anxiety sensitivity (NAS)	PI	NAS: ML ↓ PI > NL (p = 0.190) and C (p = .003)[1]<br>HAS: ML ↓ PI > C (p = .0516) but not NL (p = .816)[1]
		PT	NAS:ML ↑ PT > NL (p < .0001) and C (p < .0001)[1]<br>HAS: ML vs. NL (p = 1.0000) and C (p = .0612)
		PU	NAS: ML ↓ PU > NL (p = .0295) and C (p = .0007)[1]<br>HAS: ML vs. NL (p = 1.0000) and C (p = .294)
		PI	NSTA: ML ↓ PI > NL (p = .0499) and C (p = .02)[1]<br>HSTA: ML ↓ PI > C (p = .0431) but not NL (p = .110579)[1]
		PT	NSTA: ML ↑ PT > NL (p = .0001) and C (p < .0001)[1]<br>HSTA: ML ↑ PT > C (p = .0010) but not NL (p = .13)[1]
		PU	NSTA: ML ↓ PU > C (p < .0007) but not NL (p = .07)[1]<br>HSTA: ML vs. NL (p = .3538) and C (p = .195)[1]
		PI	NTA: ML ↓ PI > C (p = .0047) but not NL (p = .12)[1]<br>HTA: ML vs. NL (p = .1616) and C (p = .554)[1]
		PT	NTA: ML ↑ PT > NL (p = .0003) and C (p < .0001)[1]<br>HTA: ML ↑ PT > C (p = .0165) but not NL (p = .057)[1]
		PU	NTA: ML ↓ PU > C (p < .0001) but not NL (p = .086)[1]<br>HTA: ML vs. NL (p = .4017) and C (p = .291)[1]

		PI	NPA: ML ↓ PI > NL (p = .0340) and C (p = .0004)[1]<br>HPA: ML vs. NL (p = .8215) and C (p = .375)[1]
		PT	NPA: ML ↑ PT > NL (p = .0001) and C (p < .0001)[1]<br>HPA: ML ↑ PT > C (p = .0011) but not NL (p = .260)[1]
		PU	NPA: ML↓ PU > NL (p = .0379) and C (p = .001)[1]<br>HPA: ML vs. NL (p = 1.0000) and C (p = .135)[1]
Garza Villarreal 2012[29]	Music (M) vs. nature sounds (NS) vs. pink noise (PN) vs. active distraction (AD) x cognitive style (empathizer vs. systemizer vs. balanced)	PI	$F(10, 225) = 2.04$, $p = .05$<br>Systemizers ↓ PI > empathizers or balanced
Hsieh 2014[30]	Music conditioning (MC) vs. non-musical sound conditioning (SC) vs. no conditioning (NC) x expectancy-based placebo (EBP)	PI	NS[2]
		PU	NS[2]
Johnson 2020[23]	Music with relaxation suggestions vs. music alone vs. silence x hypnotizability	PTh	$r_p = .73$, $p < .05$, music only condition
		PT	NS[2]
		PI	NS[2]
		PU	NS[2]
Pando-Naude 2019[31]	Music vs. pink noise x age	rs-FC in fibromyalgia patients	NS[2]

[1]Reported only comparison of central tendencies; no treatment x moderator analysis, [2]Interaction statistic not reported

NS: non-significant, PI: pain intensity, PT: pain tolerance, PTh: pain threshold, PU: pain unpleasantness, rs-FC: resting state functional connectivity

Prediction studies

Author/year	Predictor	Pain outcome	Results
Chai 2020[32]	Baseline pain catastrophizing	PT	r = -0.273 (p = .035): ↑ BL pain catastrophizing, ↓ improvements in PT with music
Perlini 1996[33]	Pain catastrophizing	PI	r = 0.122, NS
	Perceived control	PI	r = -.278, p < .05: ↑ control, ↓ PI during music
Wilson 2024[34]	Baseline negative affect	Forearm PTh	Fibromyalgia[1]: r = -0.44, p < .01: ↑ BL negative affect, ↓ PTh<br>HC: r = -0.14, NS
		Forearm PT	Fibromyalgia: r = -.0.39, p < .05 (Fibromyalgia): ↑ BL negative affect, ↓ PT<br>HC: r = -0.16, NS
		Trapezius PTh	Fibromyalgia: r = -0.42, p < .01: ↑ BL negative affect, ↓ PTh<br>HC: r = -0.17, NS
		Trapezius PT	Fibromyalgia: r = -0.33, p < .05: ↑ BL negative affect, ↓ PT<br>HC: r = -0.08, NS
		Heat PTh	Fibromyalgia: r = -0.45, p < .01; ↑ BL negative affect, ↓ PTh<br>HC: r = -0.07, NS
		CPM	Fibromyalgia: r = -0.09, NS<br>HC: r = -0.19, NS
		TSP	Fibromyalgia: r = 0.21, NS<br>HC: r = 0.01, NS
	Baseline positive affect	PTh, PT, CPM, TSP	NS for all, except for forearm PT in HC: r = 0.24, p < .05: ↑ BL positive affect, ↑ PT
	Depression	PTh, PT, CPM, TSP	NS for all
	Anxiety	PTh, PT, CPM, TSP	NS for all, except for trapezius PTh in HC: r = -0.26 (p < .05). ↑ anxiety, ↓ PTh
	Sleep disturbance	PTh, PT, CPM, TSP	NS for all, except for CPM in Fibromyalgia:<br>r = 0.40, (p < .05): ↑ Sleep disturbance, ↑ CPM; TSP in HC: r = 0.29, (p < .05): ↑ Sleep disturbance, ↑ TSP
	Pain catastrophizing	PTh, PT, CPM, TSP	NS for all

[1] All correlational analyses in this study were limited to the favorite music condition.
CPM: Conditioned pain modulation, HC: healthy controls, NS: non-significant, PI: pain intensity, PT: pain tolerance, PTh: pain threshold, TSP: temporal summation of pain

Brain imaging candidate variables

Mediation studies

Author (year)	Comparison	Mediator	Outcome	a-path[1]	b-path[1]	a*b [95% CI] [1]	c-path[1]
Dobek (2014)[35]	Preferred music vs. no music	Neural activity of brain	PI	BA13: 4.2, p < .005<br>BA32: 3.7, p < .005<br>Parahippocampal gyrus: 7.9, p < .005<br>DLPFC (BA9): 3.6 - 4.0, p < .005<br>SI (BA3): 4.6, p < .005	NT		P<.01<br>PM ↓ PI > No music
		Neural activity of brain stem	PI	PAG: 0.9 ± 0.4 (↑), p < .05<br>RVM: 1.4 ± 0.3 (↑), p < .05	NT<br>NT		
		Neural activity of spinal cord	PI	Dorsal horn (C6): -3.4 ± 1.8<br>Ventral horn (C6): 1.4 ± 1.8	NT		
Du (2022)[3]	Music (8 - 150 Hz) vs. usual care	Brain connectivity (prefrontal cortex HbO)	PI	RBA9 vs. RBA10: r = 0.83, p < .05<br>RBA9 vs. RBA46: r = 0.90, p < .05<br>RBA10 vs. RBA46: r = 0.99, p < .01<br>RBA10 vs. LBA10 > r = 0.92, p < .01<br>Connectivity ↑ with music	RBA9 vs. RBA10: r = 0.89, p < .05<br>RBA9 vs. RBA46: r = 0.67, p < .05<br>RBA10 vs. RBA46: r = 0.86, p < .05<br>RBA10 vs. LBA10 > r = 0.78 (p < .05)		F(1, 31) = 31.50, p < .001<br>M ↓ PI > TAU

Author (year)	Comparison	Mediator	Outcome	a-path[1]	b-path[1]	a*b [95% CI]	c-path[1]
Garza-Villarreal (2015)[36]	Preferred relaxing pleasant music vs. pink noise (PN)	BOLD signal amplitude in left angular gyrus	PI	Mean vs. T = 5.05 (music > control), p = 0.008	r = −.56, p = .03	NT	p = .008<br>PM ↓ PI > PN
			PU				P = .006<br>PM ↓ PU > PN
Lu (2019)[37]	Preferred music vs. white noise (WN) vs. silence	Pre-stimulus alpha oscillations in central electrodes	PI	11.32, p = .003	1.89, p = .16	NR	p < .16<br>NS
			PU	11.32, p = .003	5.79, p < .005	NR	p < .005<br>PM ↓ PU > WN or silence
Lu (2023)[38]	Liked music (LM) vs. disliked music (DM) vs. silence	Right preCG/PoCG	PI	11.533, p < .001	0.039, p < .05	0.447 [0.095, 0.841]<br>R preCG/PoCG = mediating role	p < .001<br>LM ↓ PI > DM or silence
			PU	11.533, p < .001	0.046, NS	0.533 [-0.027, 1.128]<br>R preCG/PoCG ≠ mediating role	p < .001<br>LM ↓ PU > DM or silence
		Left Putamen	PI	9.975, p < .001	0.026, NS	0.262 [-0.052, 0.610]<br>L Putamen ≠ mediating role	p < .001<br>LM ↓ PI > DM or silence
			PU	9.975, p < .001	0.039, NS	0.390 [-0.092, 0.990]<br>L Putamen ≠ mediating role	p < .001<br>LM ↓ PI > DM or silence
		Left cerebellum	PI	10.476, p < .001	0.035, p < .01	0.364 [0.126, 0.689]<br>L cerebellum = mediating role	p < .001<br>LM ↓ PI > DM or silence
			PU	10.476, p < .001	0.044, NS	0.466 [0.110, 1.1034]<br>L cerebellum = mediating role	p < .001<br>LM ↓ PI > DM or silence

Author (year)	Comparison	Mediator	Outcome	a-path[1]	b-path[1]	a*b [95% CI]	c-path[1]
Pan (2025)[16]	Drumming, in-phase synchrony (in-S) vs. drumming, anti-phase synchrony (anti-S) vs. drumming, asynchrony (a-S)	N2-amplitudes	PI		ρ = –0.38, p < 0.001	In-S vs. a-S: –0.20 [–0.40, –0.02]<br>Anti-S vs. a-S: –0.01 [–0.20, 0.13]	c1′ = –0.44[–0.81, –0.07]<br>c2′ = –0.26[–0.72, 0.22],
			PU		ρ = –0.18, p = 0.12	NT	NT
		P2-amplitudes	PI		ρ = 0.20, p = 0.08	NT	NT
			PU		ρ = 0.20, p = 0.074	NT	NT
Pando-Naude (2019)[31]	Preferred relaxing pleasant music vs. pink noise	Angular Gyrus	PI	β = -.18, p < .05	r = −.28, p = .04	NT	p = .002<br>PM ↓ PI > PN
			PU				P = .004<br>PM ↓ PU > PN
		Posterior Cingulate Cortex	PI	β = -.17, p < .05		NT	p = .002<br>PM ↓ PI > PN
			PU				P = .004<br>PM ↓ PU > PN
		Precuneus	PI	β = -.18, p < .05		NT	p = .002<br>PM ↓ PI > PN
			PU				P = .004<br>PM ↓ PU > PN
		Amygdala	PI	β = .16, p < .05	r = .56	NT	p = .002<br>PM ↓ PI > PN
			PU				P = .004<br>PM ↓ PU > PN
		Middle Frontal Gyrus	PI	β = .18, p < .05			p = .002<br>PM ↓ PI > PN
			PU				P = .004<br>PM ↓ PU > PN

Author (year)	Comparison	Mediator	Outcome	a-path[1]	b-path[1]	a*b [95% CI]	c-path[1]
Seminowicz (2019)[39]	Uninterrupted sleep vs. forced awakenings with favorite rewarding music (FM) or neutral music NM)	Nucleus accumbens (NAc) activation	PI	Activation during pain onset in NM, pFWE = .008	β = 0.69, p < .001)	NT	β = 0.69, p < .001 FM ↓ PI > NM
			PU	right NAc ↓ activation under FM (NS)	NT	NT	β = 1.05, p < .001 FM ↓ PU > NM
		Nucleus accumbens connectivity with anterior midcingulate cortex	PI	↑ to OFC, ↓ to aMCC (only in normal sleep)	NT	NT	β = 0.69, p < .001 FM ↓ PI > NM
			PU	pFWE = .022 (↑ to OFC), ↓ to aMCC under FM (only in normal sleep)	NT	NT	β = 1.05, p < .001 FM ↓ PU > NM
Zhang (2023)[40]	Music listening vs. silence	Cerebral blood oxygen changes in PFC and motor cortex	PI	BA9: p = 0.37<br>BA46: p = .049<br>BA10: p = .047<br>BA6: p = .025	NT	NT	P < .05 Music ↓ PI > silence

[1] Effect size, test statistic, and/or p-value (depending on what is reported in the study report)
[2] No test statistic or p-value reported

ACC: Anterior Cingulate Cortex, aMCC: anterior midcingulate cortex, ANS: Autonomic nervous system, BA6: Brodmann area 6, BA9: Brodmann Area 9, BA10: Brodmann Area 10, BA46: Brodmann Area 46, NR: not reported, NS: non-significant, NT: not tested, OFC: orbitofrontal cortex, PI: pain intensity, PTh: pain threshold, PU: pain unpleasantness, Right PreCG/PoCG: Right precentral gyrus and postcentral gyrus

Social candidate variables

Mediation studies

Author (year)	Comparison	Mediator	Outcome	a-path[1]	b-path[1]	a*b [95% CI][1]	c-path[1]
Bradt (2024)[2]	Music therapy (MT) vs. social attention control (SAC)	Emotional support	Pain interference	NR	NR	NS[2]	NR
		Emotional support	PI	NR	NR	NS[2]	NR
		Emotional support	PGIC	NR	NR	NS[2]	NR
Weinstein (2016)[26]	Singing in small choir (SSC) vs. singing in large choir (SLC)	Feelings of inclusion	PTh	SSC: Z = -7.6, p < .01, r = .47<br>SLC: Z = -6.0, p < .001, r = .48<br>Singing → ↑ IOS score	NT	NT	F(1, 105) = 14.2, p < .001<br>Singing → ↑ pain threshold
		Social connectedness	PTh	SSC: Z = −7.1, p <.01, r = .44<br>SLC: Z = −7.4, p <.001, r = .50<br>Singing → ↑ social	NT	NT	F(1, 105) = 14.2, p < .001<br>Singing → ↑ pain threshold

[1] Effect size, test statistic, and/or p-value (depending on what is reported in the study report)
[2] No test statistic or p-value reported

NR: not reported, NS: non-significant, NT: not tested, PGIC: Participant perception of change, PI: pain intensity, PTh: pain threshold

Moderation studies

Author/ year	Interaction (Treatment conditions x Moderator)	Outcome	Treatment x moderator explanation and statistics (p)
Becker (2025)[1]	Self-chosen (SM) vs researcher-chosen music (RM) vs. podcast (P) X	PT, Pth, PI, PU	No significant interaction effects

Music-specific candidate variables

Experimental manipulation studies

Author/ year	Conditions	Music factor	Outcome	Means, SDs	Effect size, mean comparison statistic, p	Interpretation
Abrahan (2025)[41]	Pleasant-activating (P) vs. Unpleasant-activating (U) vs. neutral-non-activating (N)	Valence (SAM)	PI	P: 3.1 (1.74)<br>U: 3.58 (1.6)<br>N: 3.28 (1.51)	$F(2, 54)=6.93$, $p<0.01$, $\eta 2p = 0.20$.<br>P vs U: p <.05)<br>P vs N: p >.05<br>U vs N: p >.05<br>r = -0.68, p<.01	As valence ↑, PI ↓
Çift (2020)[18]	Turkish art music (TAM) vs. Western classical music (WCM) vs. patient-preferred music (PPM) vs. silence with headphones (SH) vs. silence without headphones (S)	Music preference	PI		S vs. TAM: p < .01<br>S vs. WCM: p < .05<br>S vs. PPM: p < .001<br>SH vs. TAM: p < .05<br>SH vs. WCM: p < .05<br>SH vs. PPM: p < .001<br>TAM vs. PPM: p < .05<br>WCM vs. PPM: p < .01	Music conditions ↓ PI > silence conditions, no significant difference between the 3 music conditions
		Music genre	PI	TAM: 4.5 (4 - 6)[1]<br>WCM: 5 (3 - 5.25)[1]<br>PPM: 4 (3 - 5)[1]<br>SH: 6 (5 - 7.25)[1]<br>S: 7 (6 - 7.25)[1]	S vs. TAM: p < .01<br>S vs. WCM: p < .05<br>S vs. PPM: p < .001<br>SH vs. TAM: p < .05<br>SH vs. WCM: p < .05<br>SH vs. PPM: p < .001<br>TAM vs. PPM: p < .05<br>WCM vs. PPM: p < .01	Music conditions ↓ PI > silence conditions.<br>No significant difference between the 3 music conditions.
Dunbar (2021)[42]	Music listening without movement (ML no mov) vs. music listening with head nods (MLH)	Rhythmic engagement with music (head nodding with music)	PT	ML no mov: MD = -12.2 (31.0)<br>MLH: +12.64 (32.1)	F1,31 = 5.01, p = 0.033 1-tailed	rhythmic nodding ↑ PT > no movement

Author/ year	Conditions	Music factor	Outcome	Means, SDs	Effect size, mean comparison statistic, p	Interpretation
Evers (2024)[43]	Pleasant music (PM) vs. unpleasant music (UM) vs. silence vs. baseline	Valence	Cold detection threshold (CDT)	Baseline: −1.9 ± 2.4<br>PM: −1.4 ± 0.7<br>UM: −1.9 ± 0.9<br>Silence: −1.9 ± 1.0	Omnibus: p = 0.043<br>PM vs. UM: p = 0.013	PM ↑ CDT > UM
			Warm detection threshold (WDT)	Baseline: 1.9 ± 0.5<br>PM: 2.2 ± 1.0<br>UM: 1.9 ± 0.6<br>Silence: 2.1 ± 0.7	Omnibus: p = 0.538	No difference
			Cold Pain threshold (CPT)	Baseline: 19.1 ± 8.1<br>PM: 22.2 ± 7.2<br>UM: 21.7 ± 7.3<br>Silence: 20.7 ± 7.1	Omnibus: p = 0.023<br>Baseline vs. PM: p = 0.021	PM ↑ CPT > Baseline
			Heat pain threshold (HPT)	Baseline: 41.2 ± 3.4<br>PM: 41.6 ± 3.2<br>UM: 41.2 ± 3.4<br>Silence: 41.0 ± 2.8	Omnibus: p = 0.377	No difference
			Mechanic detection threshold (MDT)	Baseline: 3.3 ± 3.0<br>PM: 2.4 ± 2.4<br>UM: 3.3 ± 2.6<br>Silence: 3.4 ± 2.6	Omnibus: p = 0.165<br>PM vs. UM: p = 0.03	PM ↓ MDT > UM
			Mechanic pain threshold (MPT)	Baseline: 30.3 ± 38.2<br>PM: 33.9 ± 39.2<br>UM: 28.1 ± 24.5<br>Silence: 28.1 ± 21.5	Omnibus: p = 0.311	No difference
			Windup ratio (WUR)	Baseline: 2.8 ± 1.2<br>PM: 2.8 ± 1.3<br>UM: 3.4 ± 1.3<br>Silence: 3.0 ± 1.7	Omnibus: p = 0.022<br>Baseline vs. UM: p = 0.021	UM ↑ WUR > Baseline

			Vibration detection threshold (VDT)	Baseline: 7.2 ± 0.5<br>PM: 7.1 ± 0.8<br>UM: 7.2 ± 0.8<br>Silence: 7.3 ± 0.9	Omnibus: p = 0.408	No difference
			Pain pressure threshold (PPT)	Baseline: 4.4 ± 1.3<br>PM: 4.3 ± 1.3<br>UM: 4.2 ± 1.3<br>Silence: 4.3 ± 1.1	Omnibus: p = 0.428	No difference
Finlay (2016)[21]	Happy music (HM) vs. sad music (SM) vs. relaxing music (RM) vs. no music (NM)	Happy-sad	PI	NR	NM vs. HM: MD = .683, SE = .271, p = .096<br>NM vs. SM: MD = .439, SE = .376, p = 1.00<br>NM vs. RM: MD = .878, SE = .309, p = .042<br>SM vs. HM: MD = .244, SE = .304, p = 1.000<br>HM vs. RM: MD = .195, SE = .275, p = 1.00<br>SM vs. RM: MD = .439, SE = .279, p = .745	Only RM was more effective than no music in ↓ PI
		Happy-sad	PT	NR	HM vs. NM: MD = 47.14, SE =11.18, p = .001<br>SM vs. NM: MD = 33.37, SE = 11.02, p = .004<br>RM vs. NM: MD = 46.58, SE =11.087, p = .001<br>HM vs. SM: MD = 13.78, SE = 1.372, p = .233<br>HM vs. RM: MD = .561, SE = 11.36, p = .961<br>SM vs. RM: MD = 13.22, SE = 8.61, p = .133	Music ↑ PT > silence. HM and SM equally effective

Author/ year	Conditions	Music factor	Outcome	Means, SDs	Effect size, mean comparison statistic, p	Interpretation
Hekmat (1993)[44]	Preferred music (PM) vs. non-preferred music (NPM) vs. silence, experimenter present (SP) vs. silence, experimenter absent (SA)	Music preference	PI		Time x group: $F(3, 76) = 0.53$, $p > .10$	No difference between groups
			PT		Time x group: $F(3,76) = 5.02$, $p < .01$	PM ↑ PT; NPM and SP no improvements; SA ↓ PT
Lu (2023)[38]	Liked music (LM) vs. disliked music (DM) vs. silence	Music preference	PI	LM: 4.81 ± 1.50<br>DM: 5.60 ± 1.52<br>Silence: 5.58 ± 1.47	$\eta_p^2 = .31$, $p < .001$	LM ↓ PI > DM or silence<br>DM vs. silence: no difference
			PU	LM: 3.57 ± 1.76<br>DM: 5.49 ± 1.81<br>Silence: 5.06 ± 1.92	$\eta_p^2 = .44$, $p < .001$	LM ↓ PU > DM or silence<br>DM - silence: no difference
Roy (2012)[9]	Pleasant-stimulating music (PSM) vs. pleasant-relaxing music (PRM) vs. unpleasant-stimulating music (USM) vs. silence	Valence	Pain rating	PSM: 43.04 (22.67)<br>PRM: 42.13 (23.49)<br>USM: 49.67 (24.24)<br>Silence: 43.80 (22.98)	$\eta^2 = 0.40$, $p < .001$<br>PSM vs. silence: NS<br>PRM vs. silence: $d = 0.46$, $p = 0.09$<br>USM vs. PSM: $d = 1.75$, $p < .001$<br>USM vs. PRM: $d = 2.16$, $p < .001$<br>PSM vs. PRM: $d = 0.35$, $p = 0.344$	PSM vs. silence: no difference<br>PRM ↓ PI > silence<br>USM ↑ PI > PSM or PRM<br>PSM vs. PRM: no difference
Seminowicz (2019)[39]	Rewarding music vs. neutral music	Valence	PI		$\beta = 0.69$ [SE = 0.10], $t = 6.83$, $p < .001$	PI ↑ during neutral music
			PU		$\beta = 1.05$ [SE = 0.12], $t = 9.03$, $p < .001$	PU ↑ during neutral music
Silvestrini (2011)[10]	Pleasant music (PM) vs. unpleasant music (UM) vs. silence vs. auditory attention task (AAT)	Valence	PI threshold	PM: 37.38<br>UM: NR<br>Silence: 30.09<br>AAT: 34.72	PM vs. silence: $t(19) = 2.94$, $p < .005$<br>UM vs. silence: $t < 1.57$, $p > .65$<br>AAT vs. silence: $t(19) = 2.91$, $p < .005$<br>PM or UM vs. AAT: $t < 1.27$, $p > .10$	PM and AAT ↑ PITh > silence<br>UM vs. silence: no difference<br>PM or UM vs. AAT: no difference

			Affective pain threshold	PM: 34.91<br>UM: NR<br>Silence: 29.18<br>AAT: 31.86	PM vs. silence: t(19) = 2.26, p < .02<br>UM vs. silence: t < 1.57, p > .65<br>AAT vs. silence: t(19) = 1.95, p < .04<br>PM or UM vs. AAT: t < 1.27, p > .10	PM and AAT ↑ affective Th > silence<br>UM vs. silence: no difference<br>PM or UM - AAT: no difference
			Sensory pain threshold	PM: 23.92<br>UM: NR<br>Silence: 20.18<br>AAT: 24.45	PM vs. silence: t(19) = 3.35, p < .002<br>UM vs. silence: t < 1.57, p > .65<br>AAT vs. silence: t(19) = 4.80, p < .001<br>PM or UM vs. AAT: t < 1.27, p > .10	PM and AAT ↑ STh > silence<br>UM vs. silence: no difference<br>PM or UM vs. AAT: no difference
			Nociceptive flexion reflex (in results they call this pain to cold pressor test	PM: 27.05<br>UM: NR<br>Silence: 22.58<br>AAT: 27.75	PM vs. silence: t(19) = 2.38, p < .02<br>UM vs. silence: t < 1.57, p > .65<br>AAT vs. silence: t(19) = 1.77, p < .05<br>PM or UM vs. AAT: t < 1.27, p > .10	PM and AAT ↑ NFR > silence<br>UM vs. silence: no difference<br>PM or UM vs. AAT: no difference
Van der Valk Bouman (2024)[45]	Urban (U) vs. electronic (E) vs. classical (C) vs. rock (R) vs. pop (P)	Music genre	PI	U: 6.1 (1.8)<br>E: 6.3 (1.6)<br>C: 6.1 (1.8)<br>R: 6.3 (1.7)<br>P: 6.0 (1.9)	p = 0.90	Music genre did not influence PI
			PU	U: 6.4 (2.0)<br>E: 6.4 (2.0)<br>C: 6.3 (2.1)<br>R: 6.4 (2.0)<br>P: 6.4 (2.2)	p = 0.99	Music genre did not influence PU
			PT	U: 118.0 (66.8)<br>E: 114.5 (67.0)<br>C: 114.3 (66.7)<br>R: 123.4 (65.1)<br>P: 108.4 (68.7)	p = 0.67	Music genre did not influence PT

Author/ year	Conditions	Music factor	Outcome	Means, SDs	Effect size, mean comparison statistic, p	Interpretation
van der Valk Bouman (2026)[12]	1 min music listening (ML) vs. 5-min ML vs. 20-min ML vs silence (S)	Music duration	PT	NR	1 vs S: -0.4 (-2.2, 1.3), p = 0.621<br>5 vs S: 0.6 (-1.2, 2.3), p = 0.524<br>20 vs S: 0.1 (-1.6, 1.9), p = 0.871<br>ML (all) vs S: p<.001	No differences between music groups<br>PT ↑ during all music conditions
			PTh	NR	1 vs S: 1.2 (-0.3, 2.7), p = 0.108<br>5 vs S: 0.9 (-0.6, 2.4), p = 0.245<br>20 vs S: 1.5 (0.1, 2.9), p = 0.038	20 ↑ PT > S
			PI	NR	1 vs S: -0.6 (-1.2, 0.0), p = 0.057<br>5 vs S: -0.6 (-1.2, 0.1), p = 0.08<br>20 vs S: -0.4 (-1.0, 0.3), p = 0.249	No sign differences between music and S
			PU	NR	1 vs S: -0.5 (-1.2, 0.1), p = 0.115<br>5 vs S: -0.9 (-1.6, -0.3), p = .007<br>20 vs S: -0.3 (-1.0, 0.3), p = 0.305	5 ↓ PU > S

Author/ year	Conditions	Music factor	Outcome	Means, SDs	Effect size, mean comparison statistic, p	Interpretation
Wilson (2024)[34]	Favorite music (FM) vs. meditative music (MM) vs. white noise (WN)	Music preference (favorite music)	Forearm PTh	Fibromyalgia:<br>FM: 3.71 ± 2.11<br>MM: 3.61 ± 1.81<br>WN: 3.44 ± 2.15	FM vs. WN: p < .05<br>MM vs. WN: p < .05<br>FM vs. MM: p > .05	FM and MM ↑ Forearm Th > WN<br>FM vs. MM: no difference
			Forearm PT	Fibromyalgia:<br>FM: 6.73 ± 4.49<br>MM: 6.11 ± 3.63<br>WN: 5.55 ± 3.45	FM vs. WN: p < .05<br>MM vs. WN: p < .05<br>FM vs. MM: p > .05	FM and MM ↑ Forearm PT > WN<br>FM vs. MM: no difference
			Trapezius PTh	Fibromyalgia:<br>FM: 6.06 ± 4.17<br>MM: 5.46 ± 3.55<br>WN: 5.18 ± 3.29	Omnibus: p = .156	No significant difference between the 3 conditions
			Trapezius PT	Fibromyalgia:<br>FM: 9.04 ± 5.54<br>MM: 8.42 ± 4.87<br>WN: 8.03 ± 5.06	FM vs. WN: p < .05<br>MM vs. WN: p > .05<br>FM vs. MM: p > .05	FM ↑ Trapezius PT > WN<br>MM vs. WN: no difference<br>FM vs. MM: no difference
			Heat PTh	Fibromyalgia:<br>FM: 39.77 ± 2.71<br>MM: 39.00 ± 2.55<br>WN: 39.58 ± 2.94	Omnibus: p = .214	No significant difference between the 3 conditions
			CPM	Fibromyalgia:<br>FM: −30.10 ± 65.19<br>MM: −12.46 ± 41.64<br>WN: −12.20 ± 33.54	Omnibus: p = .459	No significant difference between the 3 conditions
			TSP	Fibromyalgia:<br>FM: 2.39 ± 1.73<br>MM: 2.52 ± 1.95<br>WN: 2.82 ± 1.72	Omnibus: p = .096	No significant difference between the 3 conditions

Author/ year	Conditions	Music factor	Outcome	Means, SDs	Effect size, mean comparison statistic, p	Interpretation
Wright (2010)[46]	Adventure video + classical (AV+C) vs. adventure video + heavy metal (AV+HM) vs. romantic video + classical (RV + C) vs. romantic video + heavy metal (RV + HM) vs. classical music (CM) vs. heavy metal music (HM)	Music genre	PI	AV + C = 9.40 (.69)<br>AV + HM = 9.18 (.51)<br>RV + C = 7.28 (.86)<br>RV + HM = 9.15 (.64)<br>CM = 8.37 (.43)<br>HM = 9.08 (.32)		RV + CM ↓ PI the most<br>CM ↓ PI > HM
Yi (2025)[47]	Listening to preferred music at spontaneous production rate (SPR) vs. SPR +15% vs. SPR -15%	Tempo (entrainment with biological rhythm)	PI		$F[2, 118] = 6.09$, $p = 0.003$, $\eta^2 = 0.094$<br>SPR vs SPR-15%: $d = 0.27$, $p = 0.01$<br>SPR vs. SPR +15%: $d = 0.23$, $p = 0.02$<br>SPR-15% vs SPR + 15%: $p = 1.00$	PI was reduced most during listening at SPR
Zhao (2009)[48]	Happy music (HM) vs. sad music (SM) vs. lecture (non-music auditory control) vs. silence	Happy-sad	PI	HM: 4.54, 1.21<br>SM: 4.47, 1.28<br>Lecture: 5.08, 1.47<br>Silence: 4.95, 1.14	$X^2 = 16.5$, $p <.001$	HM and SM ↓ PI > lecture and silence
		Happy-sad	Pain distress	HM: 2.95, 1.52<br>SM: 3.11, 1.38<br>Lecture: 3.68, 1.66<br>Silence: 3.55, 1.56	$F(3.57) = 4.05$, $p = .011$	HM ↓ distress > Lecture or silence; SM did not ↓ distress
		Happy-sad	PT		NS[2]	No differences between conditions
		Valence	PI		$r = 0.31$, $p = 0.06$	No significant association between valence and PI
		Valence	Pain distress		$r = 0.51$, $p < .001$	↑ negative valence, ↑ distress

[1] Median (Q1-Q3), [2] No statistics reported

CPM: Conditioned pain modulation, NFR: Nociceptive flexion reflex, NR: not reported, NS: non-significant, PI: pain intensity, PT: pain tolerance, PTh: pain threshold, PU: pain unpleasantness, TSP: temporal summation of pain

Mediation studies

Author/ year	Comparison	Mediator	Outcome	a-path[1]	b-path[1]	a*b [95% CI] [1]	c-path[1]
Becker (2025)[1]	Self-chosen (SM) vs researcher-chosen music (RM) vs. podcast (P)	Valence	PT	SM vs P: $p = .002$<br>RM vs P: $p = 0.36$<br>SM vs. RM: $p = 0.085$<br>SM resulted in largest valence scores	NT	NT	SM vs. P: $p = 0.003$<br>RM vs. P: $p = 0.274$<br>SM vs. RM: $p = 0.154$<br>SM ↑ PT > P, no difference between other conditions
			PTh				SM vs. P: $p = 0.034$<br>RM vs. P: $p = 0.018$<br>SM vs. RM: $p = 0.947$<br>SM, RM ↑ PTh > P, no difference between SM and RM
			PI				SM vs. P: $p < .001$<br>RM vs. P: $p = 0.803$<br>SM vs. RM: $p = 0.002$<br>SM ↓ PI > P, RM
			PU				SM vs. P: $p = 0.007$<br>RM vs. P: $p = 0.595$<br>SM vs. RM: $p = 0.075$<br>SM ↓ PI > P, no difference between other conditions
Garza-Villarreal (2012)[29]	Mozart 1 (M1) vs. Mozart 2 (M2) vs. rain (R) vs. water (W) vs. active distraction task (AD) vs. pink noise (P)	Valence	PI	$F(3.82, 171.85) = 50.06$, $p < .001$, $\eta_p^2 = .53$<br>Valence was highest for M1 and M2	$r = -.16$, $p = .006$. ↑ pleasantness ↓ PI	NT	$\eta_p^2 = .33$<br>AD ↓ PI > R, W, M1, or M2
			PU		NS	NT	$\eta_p^2 = .22$<br>M1 ↓ PU = AD ↓ PU<br>M1 and AD ↓ PU > R, W, or M2
		Arousal	PI	$F(2.80, 125.75) = 22.93$, $p = .001$, $\eta_p^2 = .34$<br>Arousal: lowest for M1 and M2	$r = .26$, $p < .001$. ↓ arousal, ↓ PI	NT	$\eta_p^2 = .33$<br>AD ↓ PI > R, W, M1, or M2
			PU		$r = .18$, $p < .005$. ↓ arousal, ↓ PU	NT	$\eta_p^2 = .22$<br>M1 ↓ PU = AD ↓ PU<br>M1 and AD ↓ PU > R, W, or M2

Author/ year	Comparison	Mediator	Outcome	a-path[1]	b-path[1]	a*b [95% CI] [1]	c-path[1]
Roy (2008)[49]	Pleasant music (PM) vs. unpleasant music (UM) vs. silence	Valence	PI	PM vs. UM: *d* = 3.02, p<.001<br>PM vs. silence: *d* = 1.22, p<.01<br>PM ↑ pleasant > silence<br>UM vs. silence: *d* = 2.58, p<.001<br>UM ↑ unpleasant > silence	r = 0.58, p < 0.05<br>↑pleasantness, ↓PI		$F(2, 34) = 6.15$, $p < 0.001$, $\eta^2 = 0.266$<br>PM ↓ PI> UM and silence<br>UM vs silence: NS
			PU	PM vs. UM: *d* = 3.02, p<.001<br>PM vs. silence: *d* = 1.22, p<.01<br>PM ↑ pleasant > silence<br>UM vs. silence: *d* = 2.58, p<.001<br>UM ↑ unpleasant > silence	r = 0.63, p < 0.05<br>↑pleasantness, ↓PU		$F(2, 34) = 6.11$, $p < 0.01$, $\eta^2 = 0.264$<br>PM ↓ PU> UM and silence<br>UM vs silence: NS
		Arousal	PI	PM vs UM: *d* = 0.41, p>.05<br>PM vs. silence: *d* =1.33, p<.001<br>PM↑arousal > silence<br>UM vs. silence: *d* = 1.58, p<.001<br>UM↑arousal > silence	r = 0.59, p < 0.05<br>PM: ↓arousal, ↓PI<br>UM: no effect		$F(2, 34) = 6.15$, $p < 0.001$, $\eta^2 = 0.266$<br>PM ↓ PI> UM and silence<br>UM vs silence: NS
			PU	PM vs UM: *d* = 0.41, p>.05<br>PM vs. silence: *d* =1.33, p<.001<br>PM↑arousal > silence<br>UM vs. silence: *d* = 1.58, p<.001<br>UM↑arousal > silence	r = 0.49, p < 0.05<br>PM: ↓arousal, ↓PU<br>UM: no effect		$F(2, 34) = 6.11$, $p < 0.01$, $\eta^2 = 0.264$<br>PM ↓ PU> UM and silence<br>UM vs silence: NS

Author/ year	Comparison	Mediator	Outcome	a-path[1]	b-path[1]	a*b [95% CI] [1]	c-path[1]
van der Valk Bouman (2026)[12]	1 min music listening (ML) vs. 5-min ML vs. 20-min ML vs silence (S)	Valence	PT	1 vs S: p = 0.027<br>5 vs S: p = 0.065<br>20 vs S: p = 0.132	NT	NT	ML vs S: p<.001<br>No differences between music groups<br>PT ↑ during all music conditions
			PTh	1 vs S: p = 0.027<br>5 vs S: p = 0.065<br>20 vs S: p = 0.132	NT	NT	1 vs S: p = 0.108<br>5 vs S: p = 0.245<br>20 vs S: p = 0.038<br>20 ↑ PT > S
			PI	1 vs S: p = 0.027<br>5 vs S: p = 0.065<br>20 vs S: p = 0.132	NT	NT	1 vs S: p = 0.057<br>5 vs S: p = 0.08<br>20 vs S: p = 0.249<br>No sign differences between music and S
			PU	1 vs S: p = 0.027<br>5 vs S: p = 0.065<br>20 vs S: p = 0.132	NT	NT	1 vs S: p = 0.115<br>5 vs S: p = .007<br>20 vs S: p = 0.305<br>5 ↓ PU > S
		Arousal	PT	1 vs S: p = 0.788<br>5 vs S: p = 0.372<br>20 vs S: p = 0.54<br>No effect on arousal	NT	NT	ML vs S: p<.001<br>No differences between music groups<br>PT ↑ during all music conditions
			PTh	1 vs S: p = 0.788<br>5 vs S: p = 0.372<br>20 vs S: p = 0.54<br>No effect on arousal	NT	NT	1 vs S: p = 0.108<br>5 vs S: p = 0.245<br>20 vs S: p = 0.038<br>20 ↑ PT > S
			PI	1 vs S: p = 0.788<br>5 vs S: p = 0.372<br>20 vs S: p = 0.54<br>No effect on arousal	NT	NT	1 vs S: p = 0.057<br>5 vs S: p = 0.08<br>20 vs S: p = 0.249<br>No sign differences between music and S
			PU	1 vs S: p = 0.788<br>5 vs S: p = 0.372<br>20 vs S: p = 0.54<br>No effect on arousal	NT	NT	1 vs S: p = 0.115<br>5 vs S: p = .007<br>20 vs S: p = 0.305<br>5 ↓ PU > S

Effect size, test statistic, and/or p-value (depending on what is reported in the study report)
NT: not tested, PI: pain intensity, PU: pain unpleasantness

Moderation studies

Author/ year	Interaction (Treatment conditions x Moderator)	Outcome	Treatment x moderator statistics, p-value Interpretation
Lad (2022)[50]	Auditory only vs. tactile only vs auditory-tactile x music preference (liked vs. disliked)	Mean force at moderate pain	Interaction effect: $F(2,66) = 4.02$, $p = .023$, $\eta^2 p = .109$<br>Auditory-tactile, liked vs. disliked: $t(33) = 4.72$, $p = .0006$<br>Force at moderate pain: ↑ for liked song > disliked song<br>No effect of song preference for auditory only and tactile only conditions.

Prediction studies

Author/ year)	Predictor	Pain outcome	Results	Interpretation
Basiński (2021)[51]	Music preference	Average PI	$\beta = -0.31$, SE = 0.03, $p = 0.002$	Preferred music ↓ average pain
		Maximal PI	$\beta = -0.09$, SE = 0.03, $p = 0.001$	Preferred music ↓ maximal pain
		PT	$\beta = 1.93$, SE = 0.44, $p = 0.0001$	Preferred music ↑ PT
		Pain controllability	$\beta = -0.06$, SE = 0.05, $p = 0.193$	Pain controllability: not predicted by music preference
		PTh	$\beta = 0.41$, SE = 0.36, $p = 0.257$	PTh: not predicted by music preference
Dunbar (2021)[42]	Frequency of listening to music	PT	$r = -.14$, $p = 0.454$	Frequency of listening to music: not associated with changes in PT
	Frequency of active music engagement	PT	$r = -0.24$, $p = 0.178$	Frequency of active music engagement: not associated with changes in PT
	Perceived musicality	PT	$r = -0.25$, $p = 0.168$	Perceived musicality: not associated with changes in PT
	Familiarity with music	PT	$r = -0.081$, $p = 0.655$	Familiarity with music: not associated with changes in PT
	Enjoyment of music	PT	$r = 0.01$, $p = 0.941$	Enjoyment of music: not associated with changes in PT

Ernberg (2020)[20]	Music preference (like-dislike)	PI peak	NS[1]	
Howlin (2021)[14]	Musicality - active engagement	PI	β = 0.65, NS	
		PU	β = 0.05, NS	
		PT	β = -1.41, NS	
	Musicality - perceptual abilities	PI	β = -0.33, NS	
		PU	β = 0.22, NS	
		PT	β = -1.22, NS	
	Musicality - musical training	PI	β = -0.53, NS	
		PU	β = -0.01, NS	
		PT	β = 0.28, NS	
	Musicality - emotional engagement	PI	β = 0.11, NS	
		PU	β = -0.17, NS	
		PT	β = 3.82, p < .05	
	Musicality - singing abilities	PI	β = -0.04, NS	
		PU	β = 0.12, NS	
		PT	β = -0.43, NS	
	Music enjoyment	PI	β = -0.13, p < .001	↑ enjoyment, ↓ PI
		PU	β = -0.18, p < .001	↑ enjoyment, ↓ PU
		PT	β = 0.65, p < .001	↑ enjoyment, ↑ PT
LiKamWa (2022)[24]	Music training	Time elapse between Pth and PT	r = 0.32, p = 0.05	↑ music training, ↑ time to reach PT
	Perceived singing proficiency	Time elapse between Pth and PT	r = 0.4, p = 0.015	↑ singing proficiency, ↑ time to reach PT
	Level of comfort singing	Time elapse between Pth and PT	r = 0.44, p < 0.01	↑ singing comfort, ↑ time to reach PT
Linnemann (2015)[7]	Happy-sad	PI	UC = −0.03, t(384) = −0.328, p = 0.743	Happy-sad: not associated with changes in PI
		Perceived control over pain	UC = 0.01, t(292) = 2.719, p = 0.007	Happier music, ↑ pain tolerance
	Arousal	PI	UC = −0.09, t(384) = −1.221, p = 0.223	Arousal: not associated with changes in PI

	Frequency of music listening	Perceived control over pain	UC = 0.01, t(1458) = 2.047, p = 0.041	↑frequency, ↑ control over pain
Mitchell (2008)[25]	Knowledge of lyrics	PT	ρ = .253, p <.05	↑ knowledge, ↑ PT
	Importance of music in daily life	PT	NS[1]	Importance of music in daily life was not related to effect of music on PT
	Reasons for listening to music	PT	NS[1]	Reasons for listening to music were not related to effect of music on PT
	Music-evoked feelings: uplifting vs. non-uplifting, thoughtful vs. non-thoughtful, cheerful vs. noncheerful	Perceived control over pain	Uplifting vs. non-uplifting: F(1, 75) = 6.15, p < .05<br>Thoughtful vs. non-thoughtful: F(1, 75) = 5.746, p < .05<br>Cheerful vs. non-cheerful: F(1, 75) = 6.2, p < .05	Uplifting ↑ perceived control > non-uplifting music<br>Thoughtful ↑ perceived control > non-thoughtful music<br>Cheerful music↑ perceived control > non-cheerful music
	Cheerful vs. noncheerful	PI and anxiety (mediator)	Anxiety: F(1, 68) = 4.518, p < .05<br>PI: F(1, 77) = 6.27, p < .05	Cheerful music ↓ anxiety and PI > non-cheerful music
	Frequency of listening to specific song used during pain experiment	Anxiety (mediator)	ρ = .238, p < .05	↑ Frequency of listening, ↓ anxiety
Werner (2023)[17]	Music preference	PI	β = -0.14, p < .001	↑ Preference, ↓ PI
	Familiarity with music	PI	β = 0.03, p = .222	Familiarity did not affect PI

[1] No statistics reported

NS: non-significant, PI: pain intensity, PT: pain tolerance, PTh: pain threshold, PU: pain unpleasantness

Demographic candidate variables

Moderation studies

Author/ year	Interaction (Treatment conditions x Moderator)	Outcome	Treatment x moderator statistics and explanation
Anglin (2021)[52]	Music vs. no-music x time x "gender"[1]	PI	F(1,87) =5.85, p=0.018<br>Music ↓ PI for women, but not for men
Kenntner-Mabiala (2007)[6]	Slow vs. moderate vs. fast tempo x sex	Arousal	F(2, 68) = 4.3, p = .034<br>Tempo ↑ arousal in females > males
		PI	F(2, 70) = 3.1, p = .051<br>Faster tempo ↑ PI for females; no effect of tempo for males
		PU	F(2, 70) = 3.2, p < .05<br>Faster tempo ↑ PU for females; no effect of tempo for males
Mitchell (2006)[53]	Preferred music vs. relaxing music vs. white noise x "gender"	PI	F(2, 51) = 6.90, p < .01, d = .21<br>Preferred music ↓ PI more than other conditions for females, no differences for males
		PT	NS[2]
		Sensory aspect of pain	NS[2]
		Affective/ evaluative aspect of pain	F(2, 51) = 4.68, p < .05, d = .16<br>Preferred and relaxing music ↓ affective aspect > white noise for females, no differences for males
		Perceived control over pain	NS
Pando-Naude (2019)[31]	Music vs. pink noise x age	PI	NS[2]
		PU	NS[2]
Shim (2017)[54]	Music vs. no-music x "gender"	PI	p = .728, no significant difference between males or females in the non-music or the music group[3]
Silvestrini (2011)[10]		PI	NS[2]

	Pleasant vs. unpleasant vs. silence vs. auditory attention control x "gender"	PU	NS[2]
Wright (2010)[46]	Music type (classical, heavy metal, no music) x time x sex	PI	Classical music ↓ PI most for females; Heavy metal ↓ PI most for men (first 30 sec); Classical music ↓ PI most for men after 30 sec[4]

[1] Unclear if gender identity or birth sex, [2] No statistics reported, [3] Did not conduct interaction analyses, [4] Interpretation based on graphs and narrative; no statistics were reported

NS: non-significant, PI: pain intensity, PT: pain tolerance, PU: pain unpleasantness

Prediction studies

Author/year	Predictor	Pain outcome	Results
Howlin (2021)[14]	Age	PI	β = 0.08 [-0.43, 0.58], Age ≠ predictor
		Pain duration	β = 0.70 [-0.93, 2.32], Age ≠ predictor
		PU	β = -0.29 [-0.84, 0.26], Age ≠ predictor
Howlin (2022)[15]	Age	PI	β = -0.09 [-0.21, 0.02], Age ≠ predictor
		PU	β = -0.12 [-0.25, 0.02], Age ≠ predictor

PI: pain intensity, PTh: pain tolerance, PU: pain unpleasantness

References

1. Becker AS, van der Valk Bouman ES, Schaap J, van Eijck K, Bierman Z, Saat C, et al. Self-chosen music as a contributor to music-induced analgesia across diverse socio-cultural backgrounds: A crossover randomised controlled trial. Eur J Pain. 2025;29(8):e70095.10.1002/ejp.70095
2. Bradt J, Leader A, Worster B, Myers-Coffman K, Bryl K, Biondo J, et al. Music therapy for pain management for people with advanced cancer: a randomized controlled trial. Psychooncology. 2024;33(10):e70005.10.1002/pon.70005
3. Du J, Shi P, Fang F, Yu H. Effect of music intervention on subjective scores, heart rate variability, and prefrontal hemodynamics in patients with chronic pain. Front Hum Neurosci. 2022;16:1057290.https://dx.doi.org/10.3389/fnhum.2022.1057290
4. Garcia RL, Hand CJ. Analgesic effects of self-chosen music type on cold pressor-induced pain: motivating vs. relaxing music. Psychol Music. 2016;44(5):967-83.10.1177/0305735615602144
5. Kavak Akelma F, Altinsoy S, Arslan MT, Ergil J. Effect of favorite music on postoperative anxiety and pain. Anaesthesist. 2020;69(3):198-204.https://dx.doi.org/10.1007/s00101-020-00731-8
6. Kenntner-Mabiala R, Gorges S, Alpers GW, Lehmann AC, Pauli P. Musically induced arousal affects pain perception in females but not in males: a psychophysiological examination. Biol Psychol. 2007;75(1):19-23.10.1016/j.biopsycho.2006.10.005
7. Linnemann A, Kappert MB, Fischer S, Doerr JM, Strahler J, Nater UM. The effects of music listening on pain and stress in the daily life of patients with fibromyalgia syndrome. Front Hum Neurosci. 2015;9:434.https://dx.doi.org/10.3389/fnhum.2015.00434
8. Ortega A, Gauna F, Munoz D, Oberreuter G, Breinbauer HA, Carrasco L. Music therapy for pain and anxiety management in nasal bone fracture reduction: randomized controlled clinical trial. Otolaryngol Head Neck Surg. 2019;161(4):613-9.10.1177/0194599819856604
9. Roy M, Lebuis A, Hugueville L, Peretz I, Rainville P. Spinal modulation of nociception by music. Eur J Pain. 2012;16(6):870-7.https://dx.doi.org/10.1002/j.1532-2149.2011.00030.x
10. Silvestrini N, Piguet V, Cedraschi C, Zentner MR. Music and auditory distraction reduce pain: emotional or attentional effects? Music Med. 2011;3(4):264-70.10.1177/1943862111414433
11. Tollabzadeh M, Rezvani AR, Behzadipour S. The effect of music therapy on pain, anxiety, perceived stress, and biochemical parameters in hospitals among patients with cancer. Shiraz E Med J. 2023;24(10).10.5812/semj-137504
12. van der Valk Bouman ES, Becker AS, Smienk M, Horstink MMB, de Vos CC, Jeekel H, et al. Duration of music listening in music-induced analgesia: A pilot randomized controlled trial. Complement Ther Med. 2026;96:103311.10.1016/j.ctim.2025.103311
13. Arıcan NB, Soyman E. A between-subjects investigation of whether distraction is the main mechanism behind music-induced analgesia. Scientific Reports. 2025;15(1):2053.10.1038/s41598-025-86445-6
14. Howlin C, Rooney B. Cognitive agency in music interventions: increased perceived control of music predicts increased pain tolerance. Eur J Pain. 2021;25(8):1712-22.https://dx.doi.org/10.1002/ejp.1780

15. Howlin C, Stapleton A, Rooney B. Tune out pain: agency and active engagement predict decreases in pain intensity after music listening. PloS one. 2022;17(8):e0271329.https://dx.doi.org/10.1371/journal.pone.0271329
16. Pan X, Xiao Y, Hu L, Lu X. Perceived sensorimotor synchrony enhances pain modulation and attenuates laser-evoked potentials. Commun Biol. 2025;8(1):1674.10.1038/s42003-025-09076-7
17. Werner LM, Skouras S, Bechtold L, Pallesen S, Koelsch S. Sensorimotor synchronization to music reduces pain. PLOS ONE. 2023;18(7):e0289302.10.1371/journal.pone.0289302
18. Çift A, Benlioğlu C. Effect of different musical types on patient's relaxation, anxiety and pain perception during shock wave lithotripsy: a randomized controlled study. Urol J. 2020;17(1):19-23.10.22037/uj.v0i0.5333
19. Colebaugh CA, Wilson JM, Flowers KM, Overstreet D, Wang D, Edwards RR, et al. The impact of varied music applications on pain perception and situational pain catastrophizing. J Pain. 2023;24(7):1181-92.10.1016/j.jpain.2023.01.006
20. Ernberg M, Al-Khdhairi D, Shkola K, Louca Jounger S, Christidis N. Experimental muscle pain and music, do they interact? J Oral Pathol Med. 2020;49(6):522-8.10.1111/jop.13067
21. Finlay KA, Anil K. Passing the time when in pain: Investigating the role of musical valence. Psychomusicology: Music, Mind, and Brain. 2016;26(1):56-66.10.1037/pmu0000119
22. Goldfine CE, Wilson JM, Kaithamattam J, Hasdianda MA, Mancey K, Rehding A, et al. Randomized trial of self-selected music intervention on pain and anxiety in emergency department patients with musculoskeletal back pain. West J Emerg Med. 2025;26(4):1112-9.10.5811/westjem.34871
23. Johnson AJ, Elkins GR. Effects of Music and Relaxation Suggestions on Experimental Pain. The International journal of clinical and experimental hypnosis. 2020;68(2):225-45.https://dx.doi.org/10.1080/00207144.2020.1719842
24. LiKamWa A, Cardoso J, Sonke J, Fillingim RB, Booker SQ. The effect of music on pain sensitivity in healthy adults. Arts & Health. 2022;14(1):66-84.https://dx.doi.org/10.1080/17533015.2020.1827278
25. Mitchell LA, MacDonald RAR, Knussen C. An investigation of the effects of music and art on pain perception. Psychol Aesthet Creat Arts. 2008;2(3):162-70.10.1037/1931-3896.2.3.162
26. Weinstein D, Launay J, Pearce E, Dunbar RIM, Stewart L. Singing and social bonding: changes in connectivity and pain threshold as a function of group size. Evol Hum Behav. 2016;37(2):152-8.10.1016/j.evolhumbehav.2015.10.002
27. Choi S, Park S-G, Lee H-H. The analgesic effect of music on cold pressor pain responses: The influence of anxiety and attitude toward pain. PloS ONE. 2018;13(8):e0201897.https://dx.doi.org/10.1371/journal.pone.0201897
28. Choi S, Park SG. Effects of anxiety-related psychological states on music-induced analgesia in cold pressor pain responses. Explore (NY). 2022;18(1):25-30.10.1016/j.explore.2021.03.003
29. Garza-Villarreal EA, Brattico E, Vase L, Ostergaard L, Vuust P. Superior analgesic effect of an active distraction versus pleasant unfamiliar sounds and music: the influence of emotion and cognitive style. PLoS One. 2012;7(1):e29397.10.1371/journal.pone.0029397

30. Hsieh C, Kong J, Kirsch I, Edwards RR, Jensen KB, Kaptchuk TJ, et al. Well-loved music robustly relieves pain: a randomized, controlled trial. PloS one. 2014;9(9):e107390.https://dx.doi.org/10.1371/journal.pone.0107390
31. Pando-Naude V, Barrios FA, Alcauter S, Pasaye EH, Vase L, Brattico E, et al. Functional connectivity of music-induced analgesia in fibromyalgia. Scient reports. 2019;9(1):15486.https://dx.doi.org/10.1038/s41598-019-51990-4
32. Chai PR, Gale JY, Patton ME, Schwartz E, Jambaulikar GD, Wade Taylor S, et al. The impact of music on nociceptive processing. Pain Med. 2020;21(11):3047-54.10.1093/pm/pnaa070
33. Perlini AH, Viita KA. Audioanalgesia in the control of experimental pain. Can J Behav Sci. 1996;28(4):292-301.10.1037/0008-400x.28.4.292
34. Wilson JM, Franqueiro AR, Edwards RR, Chai PR, Schreiber KL. Individuals with fibromyalgia report greater pain sensitivity than healthy adults while listening to their favorite music: the contribution of negative affect. Pain Med. 2024;25(5):352-61.10.1093/pm/pnae005
35. Dobek CE, Beynon ME, Bosma RL, Stroman PW. Music modulation of pain perception and pain-related activity in the brain, brain stem, and spinal cord: a functional magnetic resonance imaging study. J Pain. 2014;15(10):1057-68.https://dx.doi.org/10.1016/j.jpain.2014.07.006
36. Garza-Villarreal EA, Jiang Z, Vuust P, Alcauter S, Vase L, Pasaye EH, et al. Music reduces pain and increases resting state fMRI BOLD signal amplitude in the left angular gyrus in fibromyalgia patients. Front Psychol. 2015;6:1051.https://dx.doi.org/10.3389/fpsyg.2015.01051
37. Lu X, Thompson WF, Zhang L, Hu L. Music reduces pain unpleasantness: evidence from an EEG study. J Pain Res. 2019;12:3331-42.10.2147/JPR.S212080
38. Lu X, Hou X, Zhang L, Li H, Tu Y, Shi H, et al. The effect of background liked music on acute pain perception and its neural correlates. Hum Brain Mapp. 2023;44(9):3493-505.10.1002/hbm.26293
39. Seminowicz DA, Remeniuk B, Krimmel SR, Smith MT, Barrett FS, Wulff AB, et al. Pain-related nucleus accumbens function: modulation by reward and sleep disruption. Pain. 2019;160(5):1196-207.https://dx.doi.org/10.1097/j.pain.0000000000001498
40. Zhang J, Shi P, Du J, Yu H. A study based on functional near-infrared spectroscopy: cortical responses to music interventions in patients with myofascial pain syndrome. Front Hum Neurosci. 2023;17:1119098.https://dx.doi.org/10.3389/fnhum.2023.1119098
41. Abrahan VD, Alba G, Justel N, Muñoz MA. The predominant role of musical valence over arousal in pain modulation: A psychophysiological study. Int J Psychol. 2026;61(1):e70142.10.1002/ijop.70142
42. Dunbar RIM, Pearce E, Tarr B, Makdani A, Bamford J, Smith S, et al. Cochlear SGN neurons elevate pain thresholds in response to music. Sci Rep. 2021;11(1):14547.https://dx.doi.org/10.1038/s41598-021-93969-0
43. Evers S, Brameyer H, Pogatzki-Zahn E. The impact of music perception on quantitative sensory testing (QST). J Clin Med. 2024;13(9).10.3390/jcm13092471
44. Hekmat HM, Hertel JB. Pain attenuating effects of preferred versus non-preferred music interventions. Psychol Music. 1993;21(2):163-73.10.1177/030573569302100205

45. Van der Valk Bouman ES, Becker AS, Schaap J, Berghman M, Oude Groeniger J, Van Groeningen M, et al. The impact of different music genres on pain tolerance: emphasizing the significance of individual music genre preferences. Sci Rep. 2024;14(1):21798.10.1038/s41598-024-72882-2
46. Wright T, Raudenbush B. Interaction effects of visual distractions, auditory distractions and age on pain threshold and tolerance. North Am J Psychology. 2010;12(1):145-58
47. Yi W, Palmer C, Serian A, Roy M. Individualizing musical tempo to spontaneous rates maximizes music-induced hypoalgesia. PAIN. 2025;166(8):1761-8.10.1097/j.pain.0000000000003513
48. Zhao H, Chen ACN. Both happy and sad melodies modulate tonic human heat pain. J Pain. 2009;10(9):953-60.https://dx.doi.org/10.1016/j.jpain.2009.03.006
49. Roy M, Peretz I, Rainville P. Emotional valence contributes to music-induced analgesia. Pain. 2008;134(1-2):140-7.10.1016/j.pain.2007.04.003
50. Lad D, Wilkins A, Johnstone E, Vuong QC. Feeling the music: The feel and sound of songs attenuate pain. Brit J Pain. 2022;16(5):518-27.https://dx.doi.org/10.1177/20494637221097786
51. Basiński K, Zdun-Ryżewska A, Greenberg DM, Majkowicz M. Preferred musical attribute dimensions underlie individual differences in music-induced analgesia. Sci Rep. 2021;11(1):8622.10.1038/s41598-021-87943-z
52. Anglin C, Knoll P, Mudd B, Ziegler C, Choi K. Music's effect on pain relief during outpatient urological procedures: a single center, randomized control trial focusing on gender differences. Transl Androl Urol. 2021;10(6):2332-9.10.21037/tau-20-1311
53. Mitchell LA, MacDonald RA. An experimental investigation of the effects of preferred and relaxing music listening on pain perception. J Music Ther. 2006;43(4):295-316.10.1093/jmt/43.4.295
54. Shim JS, Chae JY, Kang SG, Park JY, Bae JH, Kang SH, et al. Can listening to music decrease pain, anxiety, and stress during a urodynamic study? a randomized prospective trial focusing on gender differences. Urology. 2017;104:59-63.10.1016/j.urology.2017.02.035
